# Bifunctional glycolipids targeting TLR4·MD-2 and short pentraxins

**DOI:** 10.1039/d5cb00324e

**Published:** 2026-02-24

**Authors:** Daniele Zucchetta, Lena Nuschy, Simon Gumpelmair, Peter Steinberger, Iain Wilson, Holger Heine, Alla Zamyatina

**Affiliations:** a Department of Natural Sciences and Sustainable Resources, Institute of Organic Chemistry, BOKU University Vienna Austria alla.zamyatina@boku.ac.at; b Department of Natural Sciences and Sustainable Resources, Institute of Biochemistry, BOKU University Vienna Austria; c Institute of Immunology, Center for Pathophysiology, Infectiology and Immunology, Medical University of Vienna Vienna Austria; d Research Group Innate Immunity, Research Center Borstel—Leibniz Lung Center, Airway Research Center North (ARCN), German Center for Lung Research (DZL) Borstel Germany

## Abstract

Innate immune detection of pathogen- and danger-associated molecular patterns (PAMPs/DAMPs) centres on pattern-recognition receptors, with the TLR4/MD-2 complex being uniquely sensitive to trace levels of lipopolysaccharide (LPS) as well as infection-triggered endogenous ligands. While this axis rapidly induces protective cytokine production and upregulation of co-stimulatory molecules, its malfunction can cause pathological hyperinflammation culminating in systemic inflammatory response syndrome (SIRS), highlighting the importance of the development of TLR4 antagonists for the management of immunopathological disorders. Cationic antimicrobial peptides (CAMPs) naturally neutralise LPS by engaging the anionic phosphate groups of lipid A; however, many bacteria evade CAMPs by masking these phosphates with phosphoethanolamine (PE), thereby attenuating electrostatic recognition. In parallel, the PE motif on pathogenic glycans is recognised by the mammalian pentraxins C-reactive protein (CRP) and serum amyloid P component (SAP), which activate complement cascade and play central roles in innate immunity. Building on this paradigm, and analogous to bacterial lipid A remodeling, we synthesised PE-decorated, diglucosamine-based TLR4 antagonists in a highly convergent manner using phosphoramidite and H-phosphonate approaches and evaluated their immunomodulatory activity, biophysical behaviour, and pentraxin recognition. In primary human mononuclear cells, PE-decorated glycolipids attenuated cytokine secretion at micromolar levels, while biophysical analyses showed that they assemble into large, polydisperse aggregates. Zwitterionic glycolipids were recognised and bound by the human pentraxins CRP and SAP, in contrast to their ethanolamine-lacking, negatively charged bis-phosphorylated counterparts. We show that PE modification reprogrammes aggregation behaviour of glycolipids while preserving functional antagonism at TLR4 – albeit with reduced potency – and confers selective recognition by human pentraxins. These results inform the design of next-generation TLR4 antagonists aimed at minimising CAMP sequestration while maintaining efficacy against TLR4-mediated inflammation, with the added potential to engage acute-phase pentraxins.

## Abstract 2

Synthetic zwitterionic PE-modified glycolipids, prepared *via* phosphoramidite and H-phosphonate chemistry, bind acute-phase proteins SAP and CRP and attenuate LPS-induced inflammation in a TLR4·MD-2-dependent manner.

## Graphical abstract

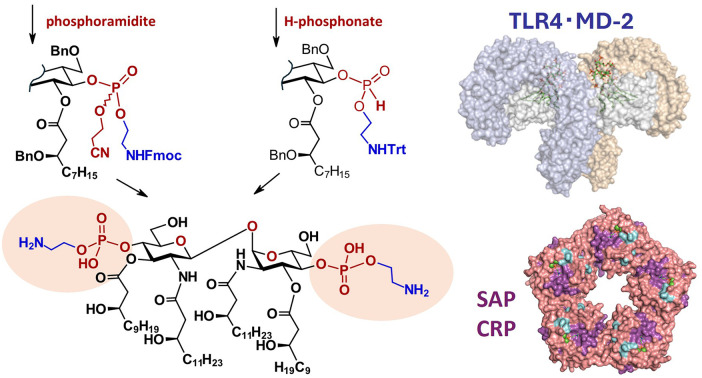

## Introduction

**Fig. 1:**
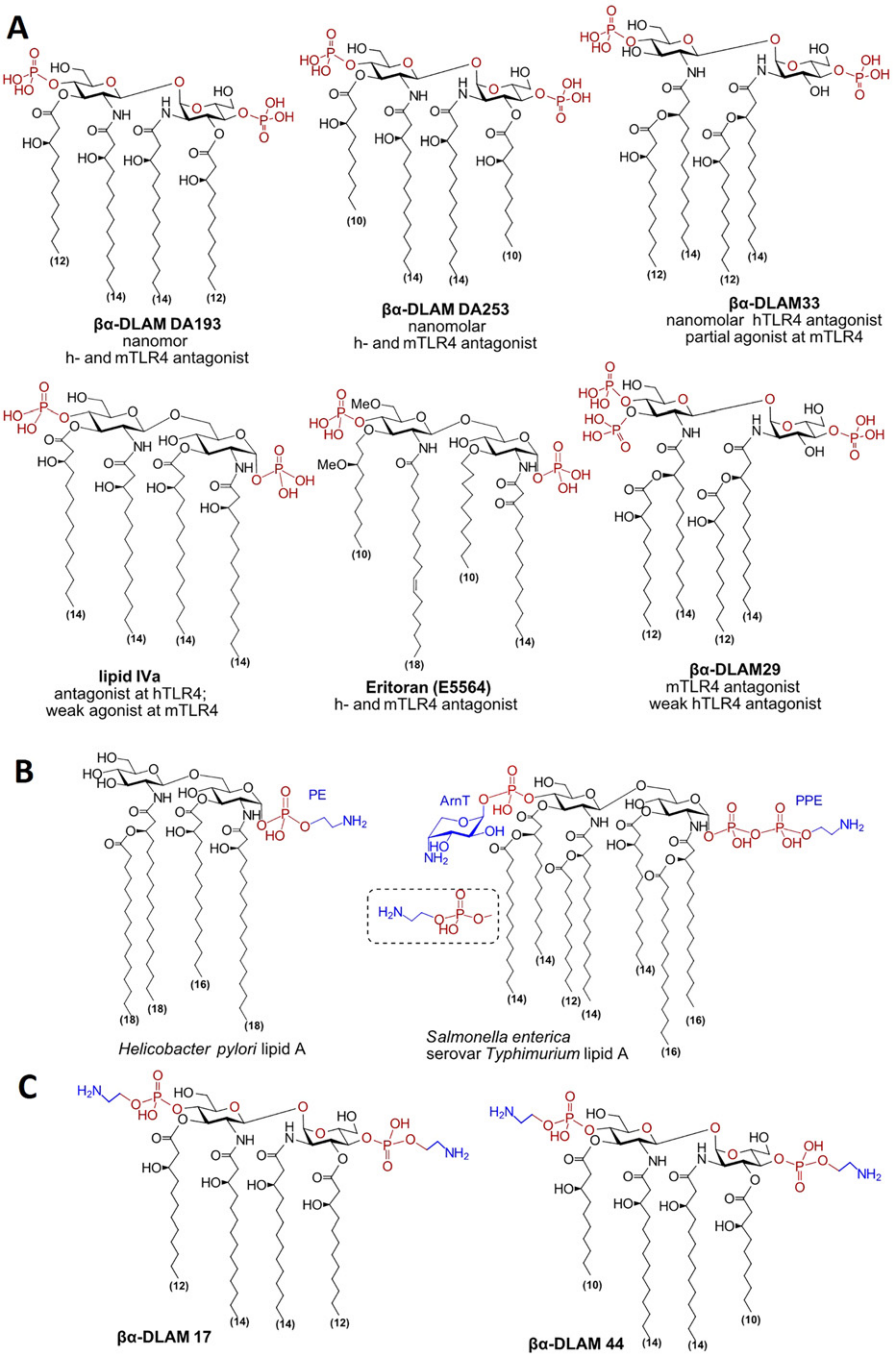
(A) Chemical structure of synthetic TLR4 antagonists; (B) examples of PE-modified lipid A variants; (C) target PE-glycolipids.

Protective inflammatory responses to pathogen- and danger-associated molecular patterns (PAMPs and DAMPs) are mediated by mammalian pattern-recognition receptors, with the TLR4/MD-2 (Toll-like receptor 4/myeloid differentiation factor 2) complex primarily responsible for sensing very low concentrations of Gram-negative lipopolysaccharide (LPS), as well as multiple endogenous ligands. Activation of TLR4/MD-2 triggers intracellular pro-inflammatory signaling cascades that induce cytokine and chemokine release and up-regulate co-stimulatory molecules, enabling a rapid host response. While indispensable for defence against infection, dysregulation of this signaling axis can unleash uncontrolled cytokine and chemokine production, culminating in systemic inflammatory response syndrome (SIRS) – a severe clinical state that can progress to multiple organ dysfunction. Accordingly, blocking uncontrolled TLR4 activation with specifically designed TLR4 antagonists has remained a prominent aim of academic and pharmaceutical research over the past decades. When considering where TLR4 antagonists might be most impactful, septic shock – responsible for roughly 11 million deaths in 2017 – typically tops the list. Sepsis – often arising from Gram-negative infections, in which LPS is a key driver of pathology – remains a serious public-health challenge.^1^ Additionally, viral infections (*e.g.*, RSV, influenza) and acute lung injury – conditions in which endogenous mediators activate TLR4 and drive inflammation – can contribute to the development of sepsis.^2–4^ Promising preclinical data and a Phase II trial advanced E5564 (Eritoran), a synthetic *R. sphaeroides* lipid A analogue and potent TLR4 antagonist, into Phase III. Despite strong activity in cell-based assays and animal models, Eritoran (Fig. 1A) did not improve survival or reduce 28-day mortality, potentially due to glycosidic phosphate loss (inactivation), HDL sequestration, and sepsis-related constraints on host immune responses.^5–7^ Despite lacking efficacy in sepsis, Eritoran has shown encouraging results in other settings for mitigating severe, virus-triggered complications.^3,4,8,9^ Dampening TLR4-mediated signaling has also shown benefit in chronic inflammation-related disorders, including arthritis,^10^ asthma,^11^ atherosclerosis,^12^ and neuroinflammation,^13^ with many other diseases potentially manageable through TLR4 modulation.

Structural studies of the TLR4/MD-2-bound natural and synthetic ligands have clarified the molecular requirements for TLR4 antagonism and activation.^14,15^ However, these studies also highlighted uncertainties arising from species-specific recognition and heterogeneity of naturally occurring ligands.^16^

Guided by these insights, we recently developed a new class of glycolipid TLR4 antagonists built on a non-reducing β,α-linked diglucosamine scaffold βGlcN(1 ↔ 1)αGlcN (‘Disaccharide Lipid A Mimetics’, βα-DLAMs) in which the rigid, specifically shaped disaccharide backbone is essential for biological activity, and the intrinsically labile glycosidic phosphate is replaced by a secondary phosphate group to improve metabolic stability (Fig. 1A).^17–20^ The number and position of phosphate groups on the nonreducing diglucosamine backbone of βα-DLAMs critically determine both inhibitory potency against LPS-induced inflammation and species-specific recognition.^21^

The phosphate groups at positions 4′ and 1 of the diglucosamine backbone in the lipid A motif of LPS can also be recognised by cationic antimicrobial peptides (CAMPs), which thereby neutralise and inactivate pathogenic LPS, constituting an early component of the innate defence against bacterial infection.^22^ However, bacterial adaptation mechanisms permit modification of the lipid A phosphate groups with positively charged appendages such as phosphoethanolamine/ethanolamine or aminosugars,^23^ thereby shielding the negative charge on the phosphates and limiting CAMP recognition, as found in the LPS of *H. pylori*,^24^*N. meningitidis*,^25,26^*S. enterica*,^23,27^*A. baumannii*,^28^*S. flexneri*^29^ and several *E. coli* strains^30,31^ (Fig. 1B). Although not yet experimentally proven, synthetic bis-phosphorylated TLR4 antagonists^18,19^ may likewise be sequestered and inactivated by CAMPs; therefore, decorating the phosphate groups of βα-DLAMs with positively charged appendages such as ethanolamine, analogous to the bacterial strategy, could render them less susceptible to neutralisation by endogenous CAMPs - an approach relevant to *in vivo* studies.

Besides sensing by CAMPs, the phosphoethanolamine (PE) moiety is known to be recognized and bound with high affinity by the soluble acute-phase proteins SAP (serum amyloid P component) and, to a lesser extent, by CRP (C-reactive protein), which has higher affinity for phosphocholine (PC)-containing headgroups.^32^ CRP is a highly conserved, pattern-recognition short pentraxin produced primarily by hepatocytes during inflammation. It circulates predominantly as a pentamer and binds a range of PC/PE-containing microbial surface ligands in a calcium-dependent manner, thereby recruiting C1q and activating the classical complement pathway to promote opsonization.^33^ Both classic LPS-driven TLR4-mediated responses and other pro-inflammatory pathways besides of TLR4 can lead to CRP production in inflammation and sepsis.^34,35^ The dual biological role of SAP – as protective in maintaining extracellular homeostasis, yet potentially pathogenic when overexpressed or mislocalised, makes it a promising therapeutic target in neurodegenerative, amyloid, and fibrotic diseases. In systemic and localised amyloidoses, SAP coats amyloid fibrils, protecting them from proteolytic clearance and promoting their persistence.^36^ In Alzheimer's disease, SAP binds amyloid-β and phosphorylated tau, stabilising neurotoxic aggregates, while SAP depletion reduces cerebrospinal SAP levels and may ameliorate neuropathology.^37^

## Results and discussion

### Rationale for the development of zwitterionically modified TLR4 antagonists.

Important consideration is that the impact of inherent PE modifications of lipid A/LPS on TLR4 activation remains largely unresolved, primarily because the relevant LPS fragments are scarce while enzymatic production or purification from natural sources is challenging. Thus, synthetic PE-analogues of nanomolar TLR4 antagonists may serve as valuable probes to interrogate TLR4 recognition and to assess how zwitterionic modifications affect the inhibition of TLR4-mediated signaling. Additionally, PE-modified bacterial glycan epitopes are expected to be recognized by the mammalian pentraxins SAP and CRP: SAP binds PE with high affinity, while CRP preferentially binds PC but can also accommodate PE in its relatively permissive binding pocket, while ligand specificity depends strongly on local conditions and on the presentation of the PE motif.^32,38^

On microbial surfaces, SAP can bind PE-modified glycan epitopes, including those on LPS/LOS, and can neutralize LPS toxicity, suggesting that SAP can engage PE-modified motifs within the core region and/or associated with the lipid A moiety of LPS.^39^ PC decoration of LPS epitopes in *H. influenzae*, of lipoteichoic acid and the CPS of *S. pneumoniae*, and of glycan epitopes in parasitic nematodes can promote pathogen persistence, while also increasing CRP/SAP recognition and thereby modulating complement activation.^40–44^

In this context, access to molecularly and structurally well-defined synthetic PE-modified glycolipids would enable a definitive assessment of their binding to the human pentraxins CRP and SAP. To this end, we chemically synthesised ethanolamine-analogues of the bisphosphorylated glycolipids – nanomolar TLR4 antagonists βα-DLAMs DA253 and DA193^17,18^ (Fig. 1C), evaluated their TLR4-mediated immunomodulatory potential in primary human immune cells, and examined whether these glycolipids are recognised by CRP and SAP.

### Chemical synthesis of PE-modified glycolipids

**Scheme 1:**
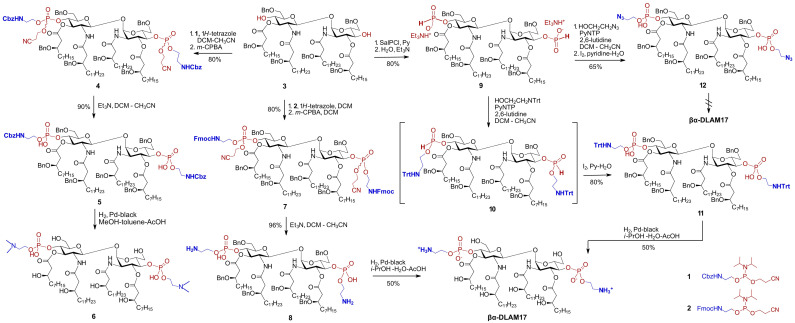
Synthesis of PE-modified glycolipid βα-DLAM17.

PE can be introduced onto carbohydrate hydroxyl groups using phosphoramidite approach^45^ with specifically designed amidite-type reagents bearing a suitably protected aminoethyl moiety, such as the *N*-Cbz-protected phosphoramidite 1 or *N*-Fmoc-protected reagent 2. Bis phosphorylated 4 was generated from the tetraacylated disaccharide precursor 3^17^ in a 1*H*-tetrazole-promoted coupling with 1 followed by oxidation with *m*-CPBA (Scheme 1). After removal of the cyanoethyl groups from the phosphates by β-elimination, the tetra-lipidated diphosphate 5 was subjected to hydrogenolysis over Pd-black (Table S1), whereby the rate and efficiency of cleaving its six benzyl and two Cbz groups were strongly dependent on the nature of the solvent. Hydrogenolytic deprotection of 5 to yield the zwitterionic βα-DLAM17 was hampered by solvent-driven side reactions and phase behaviour of highly amphiphilic, amphipatic glycolipid intermediates. Solvent mixtures that dissolved the hydrophobic substrate either gave incomplete conversion (*t*BuOH-toluene-AcOH) or promoted undesired *N*-alkylation (MeOH-toluene)^46^ with generation of *N*-methylated by-product 6, while MeOH-free variants remained sluggish. Efforts to suppress *N*-alkylation by supplementing acetic acid (MeOH-toluene-AcOH) were ineffective, and aqueous additives that typically prevent *N*-methylation were incompatible with the pronounced hydrophobicity of the substrate 5. Solvent choices constrained by *N*-methylation, together with the sharp polarity mismatch of an amphiphilic, zwitterionic target βα-DLAM17 and its partially deprotected intermediates *vs.* hydrophobic 5 likely accounted for the poor reaction outcome.

Therefore, we considered preparing an intermediate in which both PE groups could be deprotected prior to the hydrogenolysis step, which could be achieved by employing a phosphoramidite reagent bearing an *N*-Fmoc-protected aminoethyl moiety. Along these lines, 3 was phosphorylated using reagent 2 to afford 7 as a diastereomeric mixture at phosphorus (Scheme 1). Simultaneous removal of the cyanoethyl and Fmoc groups *via* base-promoted β-elimination yielded 8 in 77% yield over three steps. Hydrogenolysis of bis-PE derivative 8 achieved near-quantitative conversion to βα-DLAM17, giving a 50% isolated yield after purification on Sephadex LH-20; the increased hydrophilicity of zwitterionic intermediate 8 enabled the use of water-containing mixtures (iPrOH-H_2_O-AcOH) that completely suppressed *N*-alkylation. To expand the range of approaches for preparing PE-modified glycolipids and circumvent the challenging hydrogenolytic deprotection of the phosphate functionality, we also considered masking the aminoethyl moiety with either a temporary *N*-trityl (*N*-Trt) protecting group, which can be removed under mild acidic conditions, or an azide as a latent amine. As the phosphoramidite method is generally considered less suitable for incorporating azido group-modified functionalities due to the possibility of side reactions,^47^ and since the acid-labile *N*-trityl group is cleaved under 1*H*-tetrazole-promoted reaction conditions, we relied on the H-phosphonate approach.^48^

**Fig. 2:**
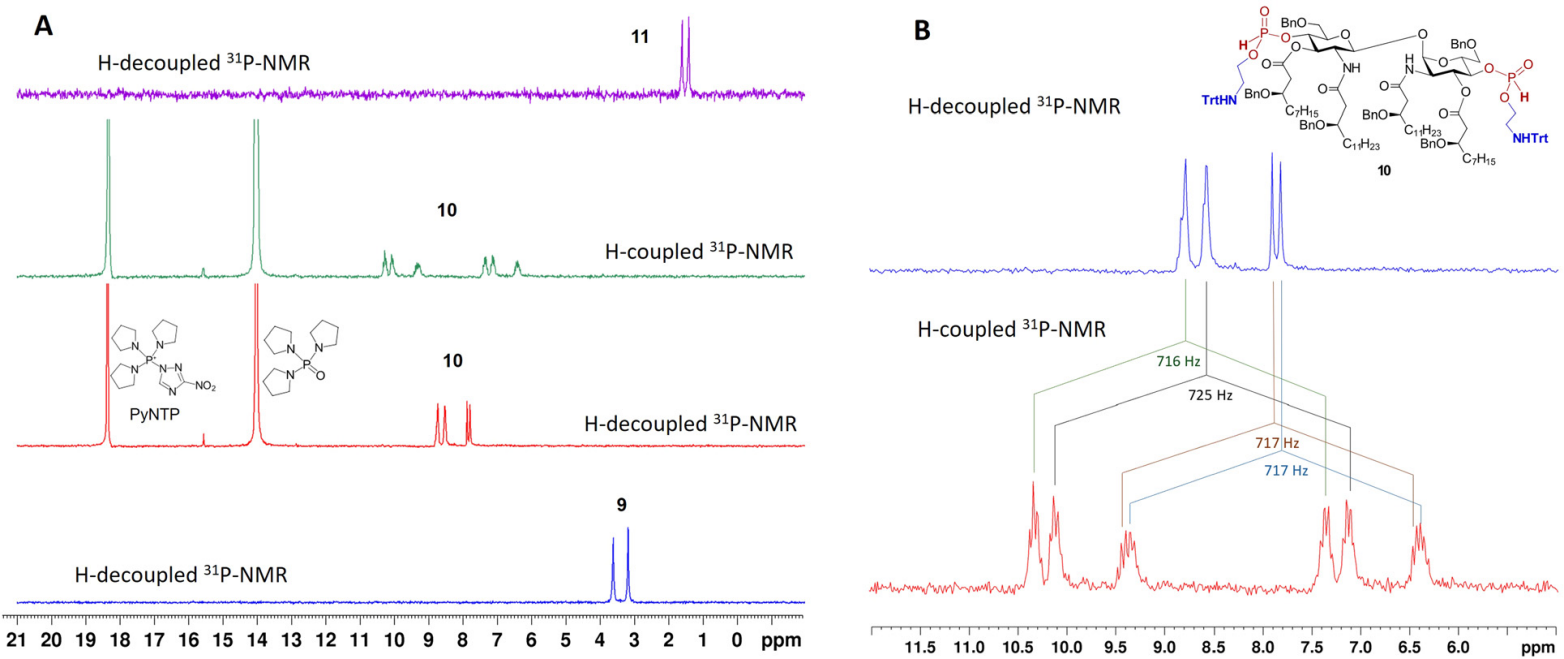
Analysis of the H-phosphonate coupling step by ^31^P-NMR. (A) The progress of the H-phosphonate coupling reaction 9 → 10 followed by ^31^P-NMR; (B) ^31^P-NMR spectrum of the H-phosphonate diester 10 prior to oxidation step.

Between the two H-phosphonate-based strategies for installing the PE substituent, *i.e.*: (i) preforming the PE-containing H-phosphonate reagent followed by coupling with a nucleophilic hydroxyl group of the sugar,^49–51^ or (ii) first preparing the glycolipid-derived H-phosphonate and then reacting it with a PE-containing nucleophile—we selected the latter approach. Phosphitylation of the free hydroxyl groups at positions 4-,4′- in 3 was performed using salicyl chlorophosphite (SalPCl)^52^ in pyridine, followed by basic hydrolysis of the cyclic intermediate to afford 9. The formation of the H-phosphonate was confirmed by ^31^P-NMR (*δ*: 3.6 and 3.2 ppm, ^2^*J*_PH_ = 650 Hz) and the appearance of a characteristic P-H-coupled signal in the ^1^H-NMR spectrum (*δ*: 6.69, 6.64 ppm, ^2^*J*_PH_ = 650 Hz) (Fig. 2).^53^

To optimize the coupling conditions of the H-phosphonate 9 with either N_3_- or *N*-Trt-protected ethanolamine derivatives, we evaluated several coupling reagents, including pivaloyl chloride (PivCl), diphenyl chlorophosphate (DPCP), and 3-nitro-1,2,4-triazol-1-yl-tris(pyrrolidin-1-yl)phosphonium hexafluorophos-phate (PyNTP),^54^ while the latter proved the most efficient. Next, the bis-H-phosphonate 9 was reacted with 2-(tritylamino)ethanol in the presence of PyNTP and 2,6-lutidine with formation of an intermediate H-phosphonate diester 10 displaying two sets of P–H-coupled signals with a characteristic coupling constants^53^ for each H-phosphonate diester group in the ^31^P-NMR spectrum (*δ*: 8.79 ppm, *J*_PH_ = 716 Hz; *δ*: 8.57 ppm, *J*_PH_ = 725 Hz; *δ*: 7.92 ppm, *J*_PH_ = 717 Hz; and *δ*: 7.83 ppm, *J*_PH_ = 717 Hz) (Fig. 2). Oxidation with aq. iodine afforded the bis-phosphodiester 11, which was globally deprotected by hydrogenolysis over Pd-black using iPrOH-H_2_O-AcOH as a solvent to afford a single product - the zwitterionic βα-DLAM17 in 50% isolated yield. The *N*-trityl protecting group on the aminoethyl moiety of 11 was cleaved within the first few minutes of the reaction in the presence of AcOH, which drastically improved solubility. Alternatively synthesised 12, in which the amino functionality was masked as an azido group, failed to afford the target zwitterionic βα-DLAM17 upon concurrent hydrogenolytic removal of the benzyl groups and catalytic reduction of the azide over Pd-black.

**Scheme 2:**
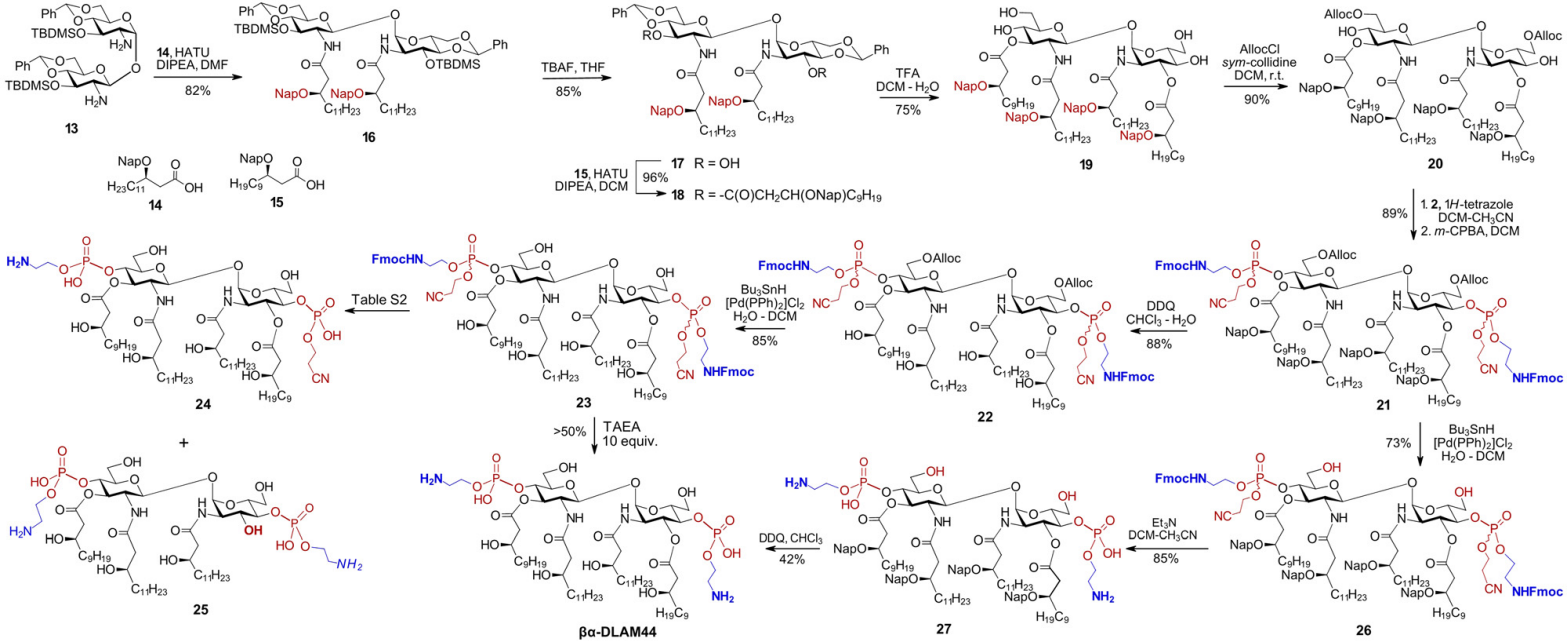
Synthesis of PE-modified glycolipid βα-DLAM44.

Benzyl ethers are the standard protecting groups for β-hydroxy functions on bacterial-type lipids,^55,56^ offering superior stability compared with ester or carbonate groups that are prone to elimination.^57^ Nonetheless, avoiding the laborious hydrogenolysis step in the synthesis of glycolipids would be advantageous, motivating replacement of all benzyl groups with temporary or semi-temporary alternatives. The 2-naphthylmethyl (Nap) group, which is highly resistant to many chemical transformations yet can be selectively removed under oxidative (DDQ) or acidic (TFA) conditions,^58^ appear to fulfil these requirements. To completely avoid the use of benzyl ethers, the C6/C6′-hydroxyl groups on the GlcN could alternatively be protected as allyloxycarbonyl (Alloc), which can be readily removed using a Pd-based catalyst in the presence of Bu_3_SnH.^59^ Along these lines, the diamine 13 was *N*-acylated with the Nap-protected long-chain β-hydroxy acid 14^60^ using HATU as the coupling reagent to afford 16 (Scheme 2).

The TBDMS-protected 3,3′-hydroxyl groups were liberated, followed by *O*-acylation with Nap-protected 15 to yield 18. After acidic hydrolysis of the 4,6-*O*-benzylidene acetal in 18, the primary C6/C6′OH groups in the resulting tetraol 19 were regioselectively protected with Alloc group to give 20. Phosphorylation of the C4/C4′-hydroxyl groups in 20 according to the phosphoramidite approach using the *N*-Fmoc-protected reagent 2 afforded the bisphosphate 21 as a diastereomeric mixture at phosphorus. Two possible options were considered for global deprotection: (i) first unmasking the β-hydroxyl groups of the lipid chains and the C6–OH groups of the sugar moiety in 21, followed by deprotection of the phosphate and aminoethyl groups or (ii) reversing this order by first removing the Alloc group from the sugar portion of 21, followed by β-elimination to cleave the cyanoethyl and *N*-Fmoc groups, and finally Nap-deprotecting the β–OH groups on the lipid chains. Initially releasing the six hydroxyl groups on both the lipids and GlcN in the first option would render the molecule moderately amphiphilic while keeping it uncharged, as the phosphate group would be deprotected last. According to the second variant, the initially released zwitterionic PE group and the two GlcN–C6–OH groups would greatly increase amphiphilicity, making the diglucosamine diphosphate “head group” highly polar/charged and keeping the lipid portion highly hydrophobic. Recognising that the chemical transformation of glycolipids is governed not only by intrinsic reactivity and chemical properties, but also by biophysical features such as amphiphilicity and aggregation state, we evaluated both deprotection strategies.

According to the first approach, the Nap groups were readily removed with DDQ to give 22, and the C6/C6′-*O*-Alloc groups were cleaved using the Bu_3_SnH/[Pd(PPh)_2_]Cl_2_ complex to afford 23. Further deprotection of the phosphate moiety in 23 by β-elimination proved extremely challenging and resulted in the formation of multiple by-products, such as 24, where one of the aminoethyl moieties is cleaved (Table S2). Application of the non-nucleophilic base DBU led to rapid and complete conversion, but promoted the formation of dibenzofulvene (DBF)-containing adducts, possibly arising from alkylation of a free amine by the released DBF.^61^ Alternatively, a combination of DBU and piperidine, with the latter serving as a DBF scavenger, afforded βα-DLAM44 within 1 h of reaction, although the product was again contaminated with inseparable DBF-derived impurities. Then, tris(2-aminoethyl)amine (TAEA) was chosen as a polar base to trap DBF,^62^ converting 23 to βα-DLAM44 without formation of detectable DBF adducts. However, TAEA basicity induced cleavage of at least one acyl chain with formation of underacylated 25; lowering TAEA concentration mitigated acyl chain cleavage but also slowed the reaction and prevented complete conversion (Table S2).

Therefore, an alternative deprotection sequence was considered, starting with the removal of the C6/6′-*O*-Alloc groups to give 26, followed by the deprotection of the Fmoc carbamate and the *O*-cyanoethyl group at the phosphate by β-elimination. The latter step proceeded smoothly under typical conditions (Et_3_N), affording zwitterionic intermediate 27. Final deprotection of the β-hydroxyl groups on the lipid chains using DDQ produced the target βα-DLAM44, isolated after multiple purification steps to remove persisting hydrophobic reagent adducts.

### Immunobiological evaluation of synthetic PE-glycolipids

**Fig. 3:**
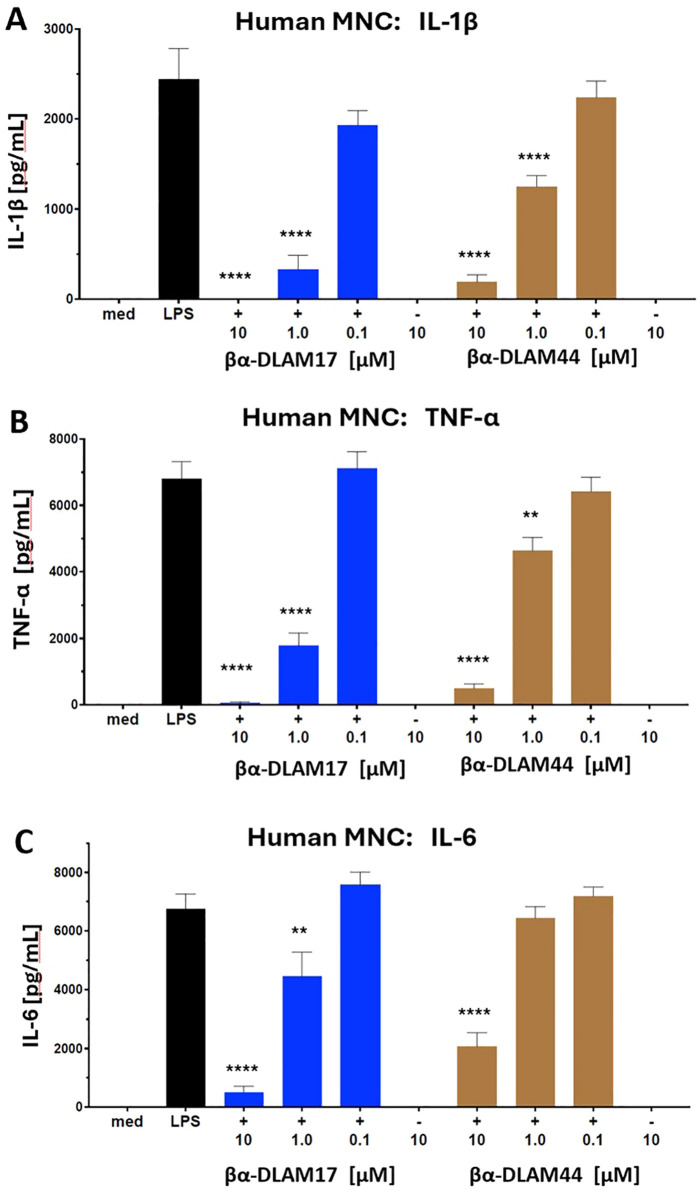
Antagonistic potential of βα-DLAM17 and βα-DLAM44 in human mononuclear cells (MNC). Inhibition of *E. coli* O111:B4 LPS [10 ng mL^−1^]-induced release of IL-1β (A), TNF-α (B), and IL-6 (C). Data shown are combined from *n* = 5 independent donors; error bars indicate standard error of the mean.

The variably acylated, zwitterionic PE-containing glycolipids βα-DLAM17 and βα-DLAM44 were initially evaluated for TLR4-antagonistic activity in reporter cell lines, including hTLR4/MD-2/CD14-transfected Jurkat E6.1 cells^63^ (Fig. S1) and mTLR4/MD-2 transfected HEK293 cells (Fig. S2), where they suppressed LPS-induced cell activation. Subsequently, the βα-DLAMs were tested for their ability to inhibit LPS-induced cytokine release in primary immune cells – human mononuclear cells (MNCs) (Fig. 3) and mouse macrophages (Fig. S2). Both compounds inhibited the LPS-induced release of IL-1β, IL-6, and TNF-α in MNC, although with different potency depending on lipid chain length. The shorter-chain molecule βα-DLAM17, bearing two *N*-linked C_14_-β-hydroxy lipids and two C_10_-acyloxy chains at positions 3-,3′-, exhibited greater potency in suppressing cytokine release in MNCs, with a concentration of 1 µM sufficient to fully block IL-1β and TNF-α production (Fig. 3A and B) and 10 µM required to antagonize LPS-induced IL-6 release (Fig. 3C).

However, the inhibitory effectiveness of βα-DLAM17 and βα-DLAM44 on LPS-induced inflammation was ≈100 fold lower than that of their PE-nonmodified, negatively charged counterparts DA193 and DA253.^17^ This attenuation of activity could reflect ineffective or missing ionic contacts between ethanolamine-masked phosphates and the Arg/Lys-residues lining the upper area of the MD-2 pocket. The lower potency of PE-modified βα-DLAMs may also reflect less efficient ligand delivery by CD14, which shuttles monomeric ligands into the MD-2 pocket and strongly enhances TLR4/MD-2 sensitivity.^64^ Although the LPS-CD14 binding mode remains unresolved, CD14 apparently presents positively charged surface patches that can engage ligand phosphate groups.^65^ Replacing both phosphates with PE could therefore weaken CD14-mediated recognition of PE-DLAMs, requiring higher concentrations to achieve antagonism. Alternatively, reduced potency may stem from limited glycolipid access to LBP, which extracts LPS/lipid A from membrane-like assemblies as the supramolecular organization of aggregates controls this accessibility and thus glycolipid-protein interactions.

**Fig. 4:**
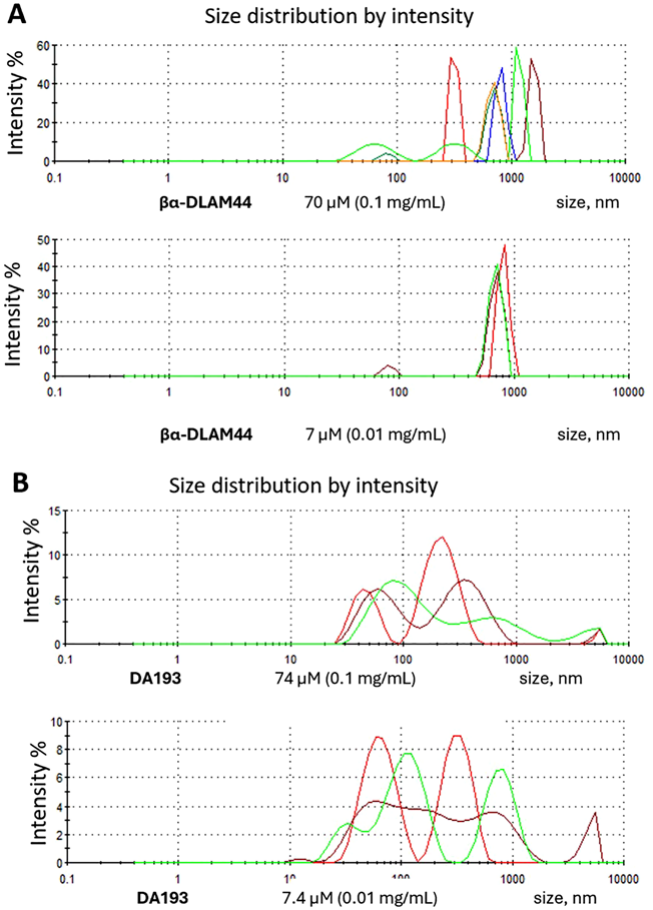
Analysis of aggregation properties of (A) PE-modified βα-DLAM44 and (B) unmodified diphosphate DA193 by DLS.

To clarify this issue, we investigated the aggregation behaviour of βα-DLAM44, as well as of liposomes composed of 50% βα-DLAM44 and 50% cholesterol at various concentrations using dynamic light scattering (DLS). βα-DLAM44 alone formed heterogeneous aggregates with a broad size distribution ranging from 500 to 1000 nm at a concentration of 0.1 mg mL^−1^ (70 µM). A tenfold dilution (0.01 mg mL^−1^, 7 µM) improved sample homogeneity, though the average particle size remained large (≈800 nm) (Fig. 4A). By contrast, the aggregate size recorded for the unmodified bisphosphorylated nanomolar TLR4/MD-2 antagonist DA193 was significantly smaller, albeit still broadly distributed (70–400 nm) (Fig. 4B).

**Fig. 5:**
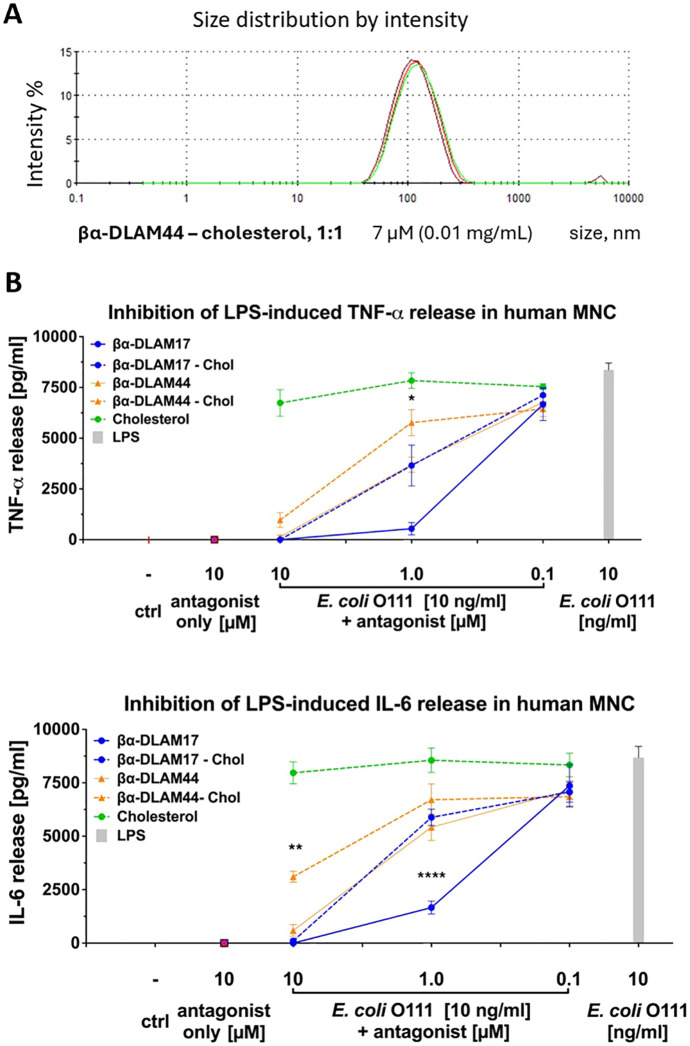
(A) Liposomal formulation of βα-DLAM44 analysed by DLS; (B) TLR4-antagonistic potential of PE-modified glycolipids included in cholesterol-containing liposomes. Data shown are combined from *n* = 5 independent donors; error bars indicate standard error of the mean.

Incorporation of cholesterol (βα-DLAM44 – cholesterol, 1 : 1) into the βα-DLAM44 formulation provided uniform liposomes with diameters of 100–200 nm at a concentration of 0.1 mg mL^−1^ (70 µM), which decreased to ≈120 nm upon dilution to 0.01 mg mL^−1^ (7 µM) (Fig. 5A). However, homogeneous small-size liposomal formulations of βα-DLAM44 and βα-DLAM17 showed lower TLR4-antagonistic activity than the corresponding glycolipids alone, despite their improved solubility (Fig. 5B). These findings suggest that large heterogeneous membrane-like aggregates present TLR4 ligands in a more accessible form to proteins of the LPS-transfer cascade, an insight that informs the design and optimisation of potential drug formulations.

**Fig. 6:**
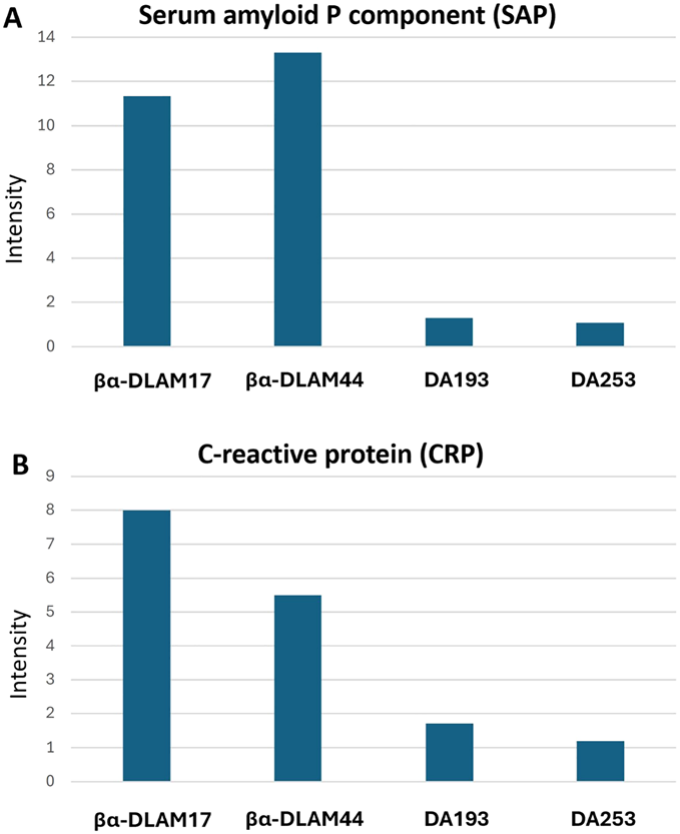
Interaction of βα-DLAMs with (A) SAP and (B) CRP by dot blot assay with quantification by densitometry using relative quantification by ImageJ.

To investigate whether the PE modification of phosphate groups in TLR4 antagonists is recognised by PE/PC-sensing proteins, we conducted a “dot blot” binding assay using nitrocellulose membranes. Synthetic glycolipids, both PE-modified and unmodified, were immobilised on the membrane and subsequently probed with human serum amyloid P component (SAP) and C-reactive protein (CRP) (Fig. 6).

Following incubation and washing, the bound proteins were visualized by immunodetection, and the signal intensities were quantified by densitometric analysis using ImageJ software (Fig. 6). The results revealed that the zwitterionic PE-modified glycolipids were well recognised and bound by SAP, in contrast to their unmodified counterparts. CRP also interacted with the PE-modified glycolipids, albeit moderately lower than SAP, whereas the unmodified, negatively charged TLR4 antagonists exhibited only negligible binding.

### Perspectives

Outside of a possible role in sepsis, where a CRP-binding TLR4 antagonist might, in principle, combine TLR4 blockade with local binding and sequestration of elevated CRP in early disease, other applications may also be worth considering. In atherosclerosis, endothelial TLR4 signaling has been linked to inflammation and plaque instability, and both pentameric CRP and monomeric CRP (mCRP) accumulate within lesions. Because mCRP can enhance TLR4-dependent NF-κB signaling,^66–69^ a CRP-targeting TLR4 antagonist could potentially accumulate at CRP-rich plaque sites and partially disrupt CRP-TLR4 crosstalk, thereby limiting endothelial activation and leukocyte recruitment. Similarly, immunohistochemical studies of arthritic synovium report elevated CRP, which may contribute to persistent local inflammation.^70,71^ Since dampening endogenous DAMP-driven, TLR4-dependent signaling in fibroblast-like synoviocytes, macrophages, and endothelium can reduce inflammatory outcomes in rheumatic disease,^72^ bifunctional molecule such as βα-DLAM17 could be explored as a means to modulate both TLR4- and CRP-associated inflammatory pathways.

Since SAP accumulates at amyloid deposits and in inflamed tissue, a zwitterionic TLR4 antagonist that can bind to SAP could, in principle, enrich locally and dampen cytokine production without broadly suppressing systemic immunity. This hypothesis is broadly consistent with reports that TLR4 inhibition reduces neuroinflammation in Alzheimer's disease models,^73^ as well as with ongoing efforts to explore SAP-targeting approaches for potential neuroprotection.^37^ An SAP-binding TLR4 antagonist might enable more targeted modulation of neuroimmune signaling, given evidence that microglial TLR4 contributes to neuroinflammation and that pharmacological TLR4 inhibition can reduce neuroinflammatory readouts *in vivo*.^74^

## Conclusions

Bifunctional synthetic glycolipids containing PE-substituted glucosamines and bacterial-type long-chain β-hydroxy lipids can selectively inhibit TLR4-mediated pro-inflammatory signaling *in vitro*, while the PE moieties are recognised and bound by CRP and SAP. We demonstrate that pentraxin family proteins specifically recognise artificial PE-containing GlcN-derived glycolipids that form heterogeneous membrane-like interfaces. We show that the TLR4 antagonistic activity and the capacity to bind SAP and CRP can be integrated within a single synthetic glycolipid molecule. These insights will inform the development of the next generation of TLR4 antagonists, which are designed to resist CAMP sequestration while retaining TLR4-specific anti-inflammatory efficacy and targeting acute-phase pentraxins.

## Author contributions

D. Z. investigation; methodology; data curation; validation; writing – original draft; writing – review & editing; L. N. investigation; methodology; data curation; validation; S. G. methodology; data curation; P. S. methodology; data curation; I. W. resources, methodology; writing – review & editing; H. H. investigation; methodology; data curation; writing – review & editing; A. Z. conceptualisation; funding acquisition; investigation; methodology; writing – original draft; writing – review & editing.

## Conflicts of interest

There are no conflicts to declare.

## Supplementary Material

CB-OLF-D5CB00324E-s001

## Data Availability

All relevant data underlying and used in the study have been made available in the article and in the supplementary information (SI) provided alongside it. Supplementary information is available. See DOI: https://doi.org/10.1039/d5cb00324e.
