# Supplementary material for: Bifunctional glycolipids targeting TLR4·MD-2 and short pentraxins
Source: RSC Chem Biol. 2026 Feb 24;7(5):820–9. doi: 10.1039/d5cb00324e (PMC12984062; doi:10.1039/d5cb00324e)

Supplementary Information (SI) for RSC Chemical Biology.
This journal is © The Royal Society of Chemistry 2026

Supporting Information

Bifunctional glycolipids targeting TLR4·MD-2 and short pentraxins

Daniele Zucchetta, Lena Nuschy, Simon Gumpelmair, Peter Steinberger, Iain Wilson, Holger Heine, Alla Zamyatina*

Content

Optimisation of reaction conditions for global deprotection of PE-decorated glycolipid intermediates 1
Inhibition of TLR4-mediated signaling by PE-modified glycolipids in reporter cell lines and mouse macrophages 4
Experimental procedures biology 6
Experimental procedures chemistry 8
DLS measurements 23
References 24
NMR spectra of synthetic compounds 25

Optimisation of reaction conditions for global deprotection of PE-decorated glycolipid intermediates.

Optimisation of reaction conditions for global deprotection of PE-glycolipid intermediates according to Scheme 1

After removal of the cyanoethyl groups from the phosphates in compound **4** by β-elimination, the tetra-lipidated diphosphate **5** was subjected to hydrogenolysis over Pd-black under various conditions (Table S1), whilst the rate and efficiency of cleaving its six benzyl and two Cbz groups were strongly dependent on the nature of the solvent. When using *t*-BuOH–toluene (2:1) only slow and incomplete conversion was observed, with at least one Cbz group remaining and no product formation detected (Table 1, entry 1). Replacing *t*-BuOH with MeOH led, as expected, to partial methylation of the liberated amino groups,[1] but also to a much faster conversion rate producing **6** in 60% yield (Table S1, entry 2). The addition of acetic acid, which is known to suppress undesired *N*-alkylation through protonation of the amino group, did not yield the expected results and still produced substantial amounts of methylated by-product **6** (Table S1, entry 3), along with a minor proportion of **βα-DLAM17**. An attempt to completely avoid the use of methanol (*t*-BuOH–toluene–AcOH = 1:1:0.4 as the solvent) slowed the reaction and failed to achieve complete conversion, regardless of the catalyst used (Pd-black vs. $Pd(OH)_2$) (Table S1, entries 4 and 5). The amphiphilic, partially deprotected intermediates aggregated and were poorly soluble in hydrophobic media, collectively limiting catalyst access and suppressing product formation.

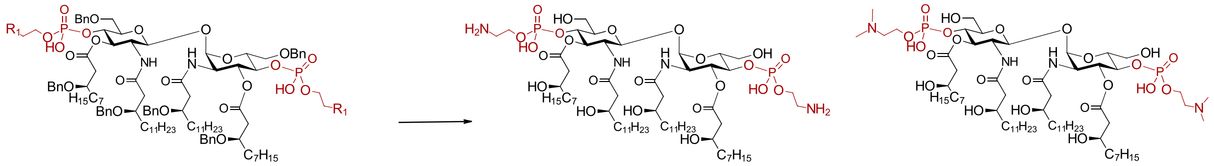

5 R_1 = NHCbz
8 R_1 = NH_2
11 R_1 = NHTr
12 R_1 = N_3

βα-DLAM17

6

Table S1. Conditions and outcome of the hydrogenolysis step towards **βα-DLAM17**.

Entry	Substrate	catalyst	Time	Solvent	Product
1	**5**	Pd Black	4 d	*t*BuOH-Tol (2:1)	0% **βα-DLAM17**[a]<br>50% Cbz-protected by-products
2	**5**	Pd Black	16 h	MeOH-Tol (2:1)	60% **6**[a]
3	**5**	Pd Black	24 h	MeOH-Tol-AcOH (1:1:0.1)	80% **6**[a]<br>10% **βα-DLAM17**
4	**5**	Pd black	4 d	*t*BuOH-Tol-AcOH (1:1:0.4)	25% **βα-DLAM17**[a]<br>15% mono-OBn, 25% di-OBn- protected by-products
5	**5**	Pd (OH)$_2$	4 d	*t*BuOH-Tol-AcOH (1:1:0.4)	30% **βα-DLAM17**[a]<br>20% mono-OBn, 20% di-OBn- protected by-products
6	**8**	Pd black	4 d	*t*BuOH-Tol-AcOH (1:1:0.4)	80% **βα-DLAM17**[a]<br>20% mono-OBn- protected by-products
7	**8**	Pd black	4 d	*i*PrOH-H_2O-AcOH (10:1:0.25)	50% **βα-DLAM17**[b]
8	**11**	Pd black	4 d	*t*BuOH-Tol-AcOH (1:1:0.4)	60% **βα-DLAM17**[a]<br>40% mono-OBn- protected by-products
9	**11**	Pd black	5 d	*t*BuOH-H_2O-AcOH (10:1:0.25)	35% **βα-DLAM17**[b]
10	**11**	Pd black	4 d	*i*PrOH-H_2O-AcOH (10:1:0.25)	50% **βα-DLAM17**[b]
11	**12**	Pd black	24 h	*t*BuOH -Tol-AcOH (1:1:0.4)	30% **βα-DLAM17**[a]<br>30% mono-OBn, 30% di-OBn-protected by-products[a]
12	**12**	Pd black	72 h	*t*BuOH -Tol-AcOH (1:1:0.8)	30% **βα-DLAM17**[a]<br>5% mono-OBn- protected by-products<br>30% hydrolysis

[a] According to LC-MS analysis
[b] Isolated yield

For global deprotection of a differently protected intermediate **7**, simultaneous removal of the cyanoethyl and Fmoc groups via base-promoted β-elimination gave **8** in 77% overall yield over three steps. Hydrogenolysis over Pd-black using *t*-BuOH–toluene–AcOH as the solvent gave a markedly improved outcome, with nearly full conversion to **βα-DLAM17** (Table S1, entry 6). The increased hydrophilicity of the zwitterionic intermediate **8** enabled the use of water-containing solvent mixtures (*i*-PrOH–H_2O–AcOH = 10:1:0.25), which completely suppressed *N*-alkylation and afforded **βα-DLAM17** in 50% isolated yield after purification on Sephadex LH-20 (Table S1, entry 7).

Alternatively, the *N*-Trt-protected (aminoethyl)-bis-phosphodiester **11** was globally deprotected by hydrogenolysis over Pd-black using *i*PrOH -H_2O-AcOH as a solvent (other solvent mixtures led to an uncomplete conversion and low yields: Table S1, entries 8-9) to afford a single product - the zwitterionic **βα-DLAM17** in 50% isolated yield (Table S1, entry 10). The *N*-Trt protecting group on the aminoethyl moiety of **11** was cleaved within several minutes in the presence of AcOH (according to LC-MS), resulting in an immediate solubility improvement in the water-containing system.

An alternatively synthesised bis-phosphodiester **12**, in which the amino functionality was masked as an azido group, failed to afford the target zwitterionic **βα-DLAM17** upon concurrent hydrogenolytic removal of the benzyl groups and catalytic reduction of the azide over Pd-black (Table 1, entries 11–12). This outcome was likely due to the use of an anhydrous solvent mixture, which is known to facilitate *N*-methylation, and to pronounced changes in amphiphilicity during the reaction that may have impeded access to the reactive centers. Solvent mixtures suitable for dissolving **5** were not adequate to fully solubilize the amphiphilic, partially deprotected intermediates, which tended to form liposome-like aggregates in hydrophobic solvents (*t*-BuOH–toluene), with the charged N-Cbz-protected PE-groups oriented toward the interior limiting accessibility.

Optimisation of reaction conditions for global deprotection of PE-decorated intermediate (23) according to Scheme 2.

Deprotection of the phosphate moiety in **23** by β-elimination of the *O*-cyanoethyl group and Fmoc carbamate proved extremely challenging, even though the analogous sequence proceeded smoothly for **7** → **8** (Table S2).

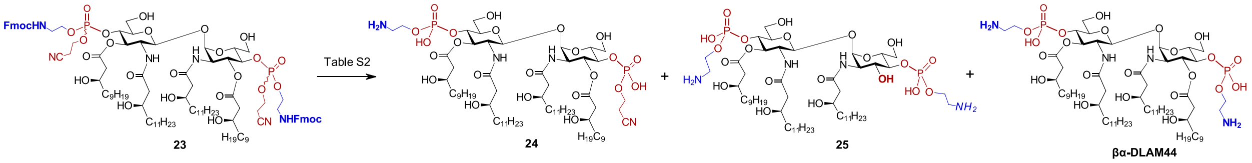

Employing 20% Et_3N in CH_2Cl_2-CH_3CN or DMSO led to cleavage of the aminoethyl moiety from one of the phosphate groups, resulting in the formation of **24** (Table S2, entries 1 and 2). Application of the strong, non-nucleophilic base 1,8-diazabicyclo[5.4.0]undec-7-ene (DBU) led to rapid and complete conversion to the desired **βα-DLAM44** but also promoted the formation of dibenzofulvene (DBF)-containing adducts, possibly arising from alkylation of a free amine by the released DBF[2] (as indicated by LC-MS and NMR analyses) (Table S2, entry 3). These adducts could not be separated despite extensive purification attempts using Bligh-Dyer lipid extraction[3] and gel-permeation chromatography. Use of bases such as piperidine or the more volatile diethylamine proved less efficient due to the formation of co-migrating by-products (Table S2, entries 4 and 5). Alternatively, a combination of DBU and the more nucleophilic piperidine, with the latter serving as a DBF scavenger, afforded **βα-DLAM44** within 1 h, which could still not be separated in pure form because of inseparable DBF-containing adducts (Table S2, entry 6). The use of alcohols to trap DBF was not considered due to the risk of partial cleavage of the phosphodiester or acyl linkages. Next, a more polar base, tris(2-aminoethyl)amine (TAEA), was selected due to its well-known ability to trap DBF[4] and its suitability for aqueous extraction from solutions of **βα-DLAM44** in hydrophobic organic solvents. Treatment of **23** with TAEA

indeed afforded **βα-DLAM44** within 1 h without any notable formation of DBF adducts. However, TAEA was sufficiently basic to promote cleavage of at least one acyl chain, yielding **25** (Table S2, entry 7). To circumvent acyl cleavage, reducing either the reaction time or the TAEA concentration slowed the progress of the transformation and did not result in complete conversion (Table S2, entries 8 and 9).

Table S2. Conditions and outcome of the deprotection step **23→βα-DLAM44**.

Entry	Reagent	Time	Solvent	Product (%)			
				**βα-DLAM44**	**24**	**25**	comigrating by-products
1	TEA, 20% v/v	4 d	CH_2Cl_2-CH_3CN	0	20 [a]	0	
2	TEA, 20% v/v	4 d	DMSO	0	70 [b]	0	
3	DBU, 20 equiv.	1h	CH_2Cl_2-CH_3CN	80 [a]	0	0	20 [a]
4	Piperidine, 20 equiv.	1 h	CH_2Cl_2-CH_3CN	70 [a]	0	0	30 [a]
5	diethylamine 20 equiv.	3 h	CH_2Cl_2-CH_3CN	50 [a]	0	0	50 [a]
6	DBU/piperidine, 20 equiv.	1 h	CH_2Cl_2-CH_3CN	90 [c]	0	0	10 [c] (DBF-adducts)
7	TAEA, 20 equiv.	1 h	CH_2Cl_2-CH_3CN	80 [c]	0	20 [c]	
8	TAEA, 20 equiv.	30 min.	CH_2Cl_2-CH_3CN	50 [c]	0	0	20 [c]
9	TAEA, 10 equiv.	1 h	CH_2Cl_2-CH_3CN	50 [c]	0	0	20 [c]

[a] According to LC-MS and TLC analysis
[b] Isolated yield
[c] According to ^{1}H NMR

Inhibition of TLR4-mediated signaling by PE-modified glycolipids in reporter cell lines and mouse macrophages

Initial evaluation of the variably acylated, zwitterionic, PE-containing glycolipids βα-DLAM17 and βα-DLAM44 in reporter cell lines, including hTLR4/MD-2/CD14-transfected Jurkat E6.1 cells (Figure S1) and mTLR4/MD-2-transfected HEK293 cells (Figure S2A), demonstrated suppression of LPS-induced cell activation at µM concentrations. Both DLAMs inhibited LPS-induced hTLR4 activation, albeit with different potencies: the shorter-chain molecule, βα-DLAM17, acylated at positions 2,2′ with two C_{14}-β-hydroxy lipid chains and at positions 3,3′ with two C_{10}-acyloxy chains, showed somewhat higher potency in suppressing hTLR4 activation in hTLR4/MD-2/CD14-transfected Jurkat E6.1 cells (Figure S1A). Consistently, βα-DLAM17 also displayed superior TLR4 inhibitory activity in the murine system, as 250 nM βα-DLAM17 reduced mTLR4-mediated IL-8 release in mTLR4/MD-2-transfected HEK293 cells by approximately twofold, whereas the same concentration of βα-DLAM44 was only marginally active (Figure S1B). Also, in immortalized mouse macrophages, a twofold inhibition of LPS-induced TNF-α production was observed for βα-DLAM17 at a concentration of 250 nM, whereas even 1000 nM of the longer-chain βα-DLAM44 was not sufficient to elicit a comparable effect (Figure S2B). Both βα-DLAM17 and βα-DLAM44 exhibited lower hTLR4-antagonizing activity than the nanomolar TLR4 antagonists DA193 and DA253, as demonstrated by assays in human MNCs (Figure S2A).

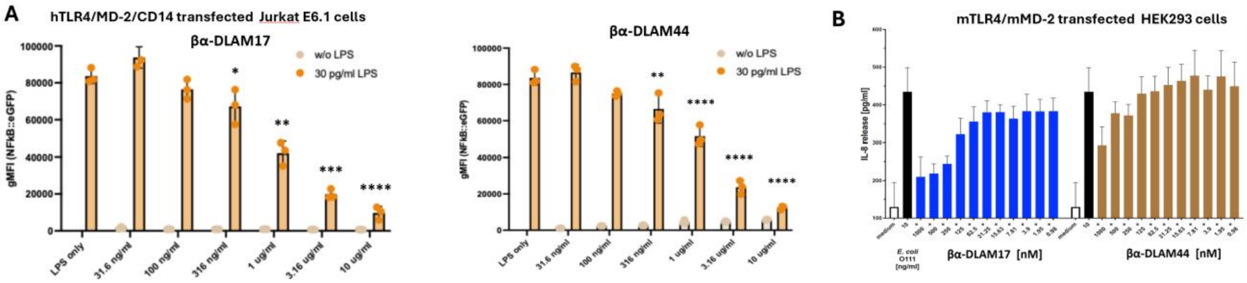

Figure S1. (A) Inhibition of *E. coli* LPS O55:B5-induced TLR4-mediated signaling in hTLR4/MD-2/CD14 transfected Jurkat E6.1 cells by βα-DLAM17 and βα-DLAM44. (B) Inhibition of *E. coli* O111:B4 LPS-induced TLR4-mediated signaling in mTLR4/MD-2 transfected HEK293 cells Experiments were performed in triplicates (mean +/- SD).

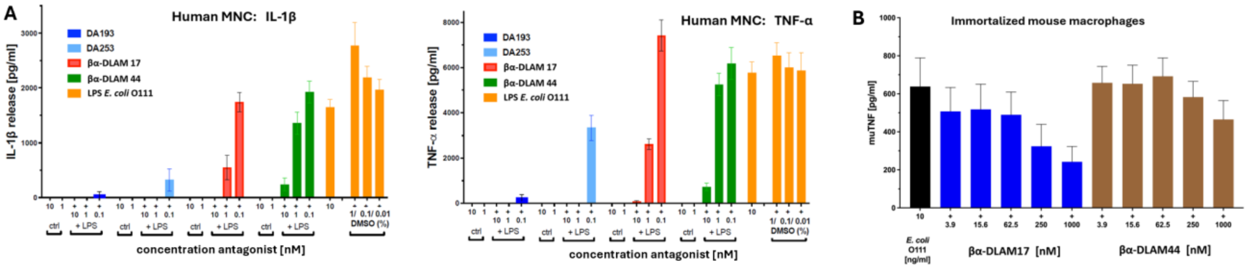

Figure S2. (B) Suppression of TNF-α release in *E. coli* O111:B4 LPS-activated mouse macrophages; (A) comparative antagonistic activities of βα-DLAM17 and βα-DLAM44 vs. DA193 / DA253 in suppressing IL-1β and TNF-α release in human MNC. Experiments were performed in triplicates.

Investigating whether zwitterionic βα-DLAMs interact with polymyxin, and whether polymyxin can neutralize TLR4 antagonists by sequestering them and preventing their interaction with TLR4/MD-2, is not straightforward because polymyxin itself can bind to and sequester LPS. The design of such experiments must also account for distinct mechanisms of action: whereas βα-DLAMs act as TLR4/MD-2 antagonists, polymyxin B antagonizes LPS. Specifically, βα-DLAMs inhibit TLR4/MD-2 by binding to the MD-2 pocket, and their antagonistic potency is determined by the relative affinities of DLAM and LPS for the MD-2/TLR4 complex. In contrast, the LPS-neutralizing activity of polymyxin B is mediated by ionically driven sequestration of LPS, and polymyxin must be applied at an approximately 1000-fold molar excess over LPS (Figure S3).

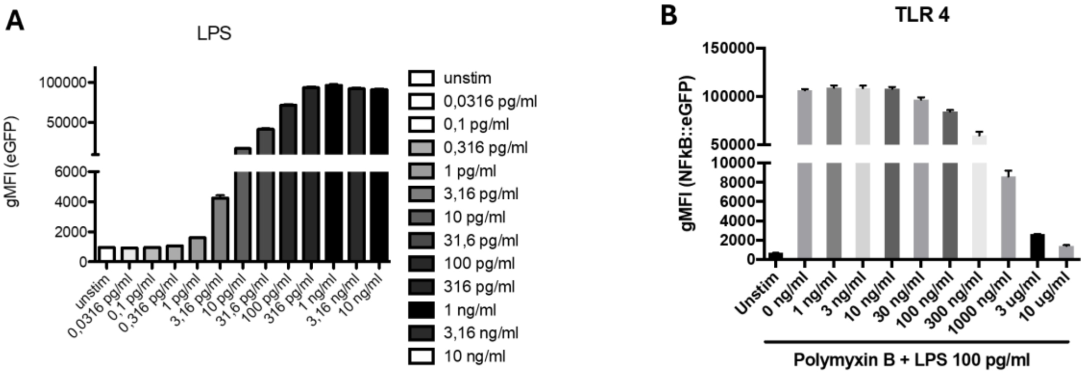

Figure S3. (A) TLR4-mediated activation of hTLR4/MD-2/CD14 transfected Jurkat E6.1 cells by increasing concentrations of *E. coli* LPS O55:B5; (B) Dose-dependent inhibition of *E. coli* LPS O55:B5-induced TLR4-mediated signaling in hTLR4/MD-2/CD14 transfected Jurkat E6.1 cells by polymyxin B.

We therefore developed a protocol in which βα-DLAMs were preconditioned with polymyxin (equimolar amounts) in solution for 1 h, followed by pre-incubation of the mixture with hTLR4/MD-2/CD14-transfected Jurkat E6.1 cells for 1 h and an additional wash step to remove excess polymyxin (and, simultaneously, unbound βα-DLAMs). Cells were then stimulated with *E. coli* LPS under standard conditions. Under this experimental setup, βα-DLAMs alone and βα-DLAMs premixed with polymyxin showed comparable inhibitory potency (Figure S4). These results suggest that the zwitterionic glycolipids do not interact measurably with the positively charged polymyxin.

Comparison of experiments including a wash step with those performed without washing indicates that polymyxin cannot be completely removed from the cell culture, leading to partial neutralization of LPS by residual polymyxin. Notably, βα-DLAM17 appears to exhibit sufficient affinity for the TLR4/MD-2 complex, as its inhibitory activity was largely retained after the wash step, to a nearly similar extent as observed in the standard (non-washed) setup.

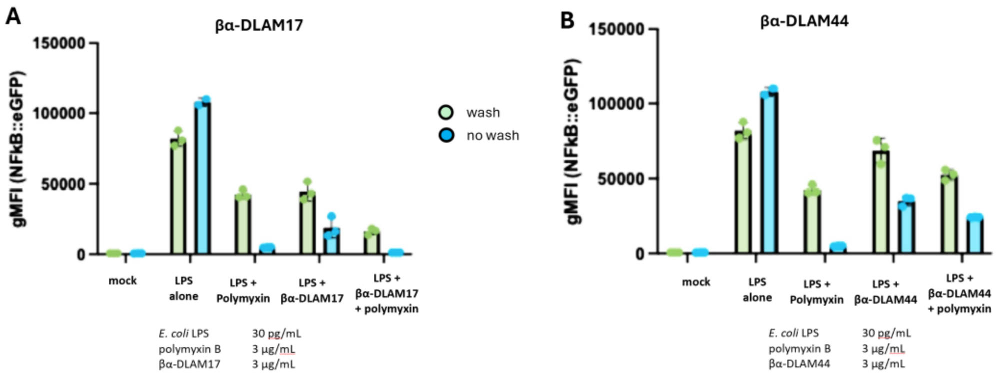

Figure S4. Inhibition of *E. coli* LPS O55:B5-induced TLR4-mediated signaling in hTLR4/MD-2/CD14 transfected Jurkat E6.1 cells by βα-DLAM17, βα-DLAM44 and polymyxin B.

Experimental procedures biology

Inhibition of TLR4-mediated signaling in hTLR4-transfected Jurkat E6.1 cells.

Highly sensitive TLR4-reporter cells from Jurkat E6.1-NF-kB::eGFP cells engineered to express human TLR4, MD-2 and CD14 has were generated as previously described.[5] TLR4-reporter cells were maintained in RPMI1640 medium supplemented with 10 % heat inactivated FBS and 100 U/ml penicillin and 100 U/ml streptomycin at 37 °C in a humidified atmosphere with 5 % CO_2. Stimulation cultures were performed under the same culture conditions in triplicates in 96 well-round bottom plates with 5 x 10^4 TLR-4 reporter cells/well in a final volume of 100 ml. The antagonists were pre-incubated with the cells at an indicated concentration for 1 h under standard culture conditions. Subsequently, *E. coli* LPS-B5 (ultrapure, InvivoGen, San Diego, CA) was added to a final concentration of 30 pg/ml. Following 20-24 h of stimulation, reporter activation was measured using a CytoFLEX S flow cytometer equipped with a plate loader (Beckman Coulter). Flow data were analyzed using FlowJo Software (v10.8.1, Treestar, Ashland, OR). For further analysis, the geometric mean fluorescence intensity (gMFI) was used.
For experiments with polymyxin, βα-DLAMs were mixed with an equimolar concentration of polymyxin for 1 h. The resulting solutions were then pre-incubated with the cells for 1 h under standard culture conditions. The βα-DLAMs alone and polymyxin alone (each at the indicated concentration) were included as positive controls. Before stimulation with *E. coli* LPS-B5, the cells were washed to remove excess antagonist and polymyxin that had not interacted with TLR4/MD-2 or βα-DLAMs, respectively. Following 24 h of stimulation, reporter activation was measured using a CytoFLEX S flow cytometer equipped with a plate loader. Flow data were analyzed using FlowJo Software. For further analysis, the geometric mean fluorescence intensity (gMFI) was used. Experiments performed with and without the intermediate wash step were compared. Statistical analysis was performed using GraphPad Prism (Version 10, GraphPad Software, Inc., La Jolla, CA). Statistics were calculated using One-way Anova followed by Dunnett's multiple comparison test (compared to LPS-only). Levels of significance were categorized as follows: ns, not significant; ns > 0.05, *p ≤ 0.05; **p ≤ 0.01; ***p ≤ 0.001; ****p ≤ 0.0001.

Inhibition of cytokine release by PE-modified glycolipids in human mononuclear cells (MNC).

MNC (peripheral human blood mononuclear cells) were prepared from heparinized blood from healthy volunteers by gradient centrifugation (Biocoll, Merck) and were subsequently incubated in 96-well tissue culture plates at a volume of 150 µL and a concentration of 1 × 106/mL using as medium RPMI-1640 supplemented with 100 U/mL penicillin (PAA Laboratories, Pasching, Austria), 100 µg/mL streptomycin (PAA Laboratories GmbH), and 10% FCS (Merck Millipore, Darmstadt, Germany). Solutions of βα-DLAMs were prepared from stock solutions in DMSO (1 mg/mL) using RPMI cell medium supplemented with 10% FCS. For inhibition experiments, cells were preincubated with the indicated amounts of βα-DLAM17 and βα-DLAM44 for 1h before cells were stimulated with *E. coli* O111:B4 LPS [10 ng/mL]. After a culture period of 20 h at 37 °C, culture supernatants were harvested and the TNF-α, IL-6 and IL-1β contents were determined using an ELISA according to the manufacturers' protocol (Thermo Fisher Scientific, Dreieich, Germany). Data shown are combined from n = 5 independent donors; error bars indicate standard error of the mean. Statistics were calculated with Prism 10. Statistical analyses were performed as follows: Figure 3: one-way ANOVA with Dunnett's multiple comparison test; adjusted p-values indicate significant differences to LPS alone: **** < 0.0001, ** < 0.005; Figure 5: two-way ANOVA with Tukeys multiple comparison test, adjusted p-values indicate significant differences between βα-DLAMs (as a stand-alone preparation or in combination with cholesterol) at the same concentration: **** < 0.0001, * < 0.05.

Ethics statement. Approval for these studies was obtained from the Institutional Ethics Committee at the University of Lübeck (Lübeck, Germany; Az. 12-202A) according to the Declaration of Helsinki. All relevant laws were followed and all donors gave written informed consent.

Inhibition of cytokine release by PE-modified glycolipids in transiently transfected HEK293 cells.

HEK293 cells (DSMZ, Braunschweig, Germany) were transfected for 24 h with plasmids coding for mouse TLR4 and MD-2 (kind gift of K. Miyake, Tokyo, Japan) using Lipofectamin2000 (Invitrogen GmbH, Karlsruhe, Germany) according to the manufacture's instruction. Solutions of βα-DLAMs were prepared from stock solutions in DMSO (1 mg/mL) using DMEM cell medium supplemented with 10% FCS. For inhibition experiments, cells were preincubated with indicated βα-DLAM for 1h before cells were stimulated with *E. coli* O111:B4 LPS [10 ng/mL] for 20h. IL-8 production was measured by human IL-8 CytoSet ELISA (Invitrogen) according to the manufacturer's instruction. Data shown are combined from n = 3 independent experiments, error bars indicate standard error of the mean. Statistics were calculated with Prism 10 (one-way ANOVA with Dunnett's multiple comparison test).

Inhibition of TNF-α release by PE-modified glycolipids in immortalized mouse macrophages.

Immortalized C57BL/6 wt mouse macrophage cell line was kindly provided by D.T. Golenbock (Worcester, MA, USA) and propagated in RPMI medium (PAA, Linz, Austria) containing 10% FCS, 20 mM HEPES buffer, 2 mM L-Glutamin (both PAA, Linz, Austria) and 20 µg/ml gentamicin (Sigma, Deisenhofen, Germany). Solutions of βα-DLAMs were prepared from stock solutions in DMSO (1 mg/mL) using RPMI cell medium supplemented with 10% FCS. Cells were preincubated for 30 min with increasing concentrations of βα-DLAMs before stimulation with 10 ng/ml of *E. coli* O111:B4 LPS. After a culture period of 20 h at 37 °C, culture supernatants were harvested and content of mouse TNF-α was determined using a CytoSet ELISA (Invitrogen GmbH, Karlsruhe, Germany) according to the manufacture's instruction. Data shown are combined from n=3 independent experiments, error bars indicate standard error of the mean. Statistics were calculated with Prism 10 (two-way ANOVA with Tukeys multiple comparison test).

Binding assay with CRP and SAP

PE-modified glycolipids (βα-DLAM17, βα-DLAM44) and their non-modified counterparts (DA193, DA253) were dissolved at 5 µg/µL in deionized water by vortex and sonication. The aqueous solutions (2 µL) were spotted on nitrocellulose membrane (Pall, Port Washington, NY, US). After complete absorption, membranes were blocked in TBS-T buffer with 0.5% BSA (Carl Roth Corporation, Karlsruhe, Germany) for 1 h at r.t. Membranes were incubated with either human C-reactive protein (MP Biochemicals, Santa Ana, US) diluted 1:200 in TBS-T supplemented with BSA and 2.5 mM $CaCl_2$ or human serum amyloid protein (Fitzgerald, Acton, MA, US) diluted 1:200 for 1 h at r.t., and washed with TBS-T; binding was detected with either anti-CRP from mouse (1:1000 diluted, R&D Systems, Minneapolis, MN, US) or anti-SAP from rabbit (1:1000 diluted, Calbiochem, San Diego, CA, US) followed by alkaline phosphatase (AP)-conjugated anti-mouse IgG (1:10000 diluted, Sigma-Aldrich, St. Louis, MO, US) or AP-conjugated anti-rabbit IgG (1:2000 diluted, Vector Laboratories, Newark, CA, US), respectively. After washing with TBS-T, blots were developed with SigmaFAST BCIP/NBT as described by the supplier. Spot intensities were quantified by densitometry (ratio of net compound dot to net dot control with local background subtraction; relative quantification was performed using ImageJ.[6#] The experiment was performed in duplicate.

[#] *Davarinejad, H. Quantifications of Western Blots with ImageJ; York University: Toronto, 2015; available at http://www.yorku.ca/yisheng/Internal/Protocols/ImageJ.pdf*

Experimental procedures chemistry

General Synthetic Methods

Reagents and solvents were purchased from commercial suppliers and used without further purification unless otherwise stated. Toluene was dried by distillation first over phosphorus pentoxide, then over calcium hydride and was then stored over activated 4 Å molecular sieves (MS). Solvents were dried by storage over activated MS for at least 24 h prior to use (dichloromethane 4 Å, toluene 4 Å, acetonitrile 3 Å, and DMF 3 Å). Residual moisture was determined by coloumbometric titration on a Mitsubishi CA21 Karl Fischer apparatus and did not exceed 20 ppm. Reactions were monitored by TLC performed on silica gel pre-coated glass plates (SIL G-25/UV254, MACHEREY-NAGEL) or on silica gel 60 F254 HPTLC precoated glass plates with a 25 mm concentration zone (MERCK). Spots were visualized by dipping into a sulfuric acid − p-anisaldehyde solution and subsequent charring at 250 °C. Solvents were removed under reduced pressure at ≤40 °C. Preparative HPLC was performed on a YMC Pack SIL-06 250 × 10 mm, S-5 µm, 6 nm column. Column chromatography and preparative MPLC were performed using silica gel PuriFlash IR-50SI (40-60 µm) and pre-packed Interchim SI-S-2G/6 and SI-S-500/6 column, respectively. Size exclusion chromatography was performed using Bio-Beads SX-1 (BioRad) support or Sephadex LH-20 support (Cytiva). NMR spectra were recorded at 298 K on a Bruker Avance III 600 spectrometer (^{1}H at 600.22 MHz; ^{13}C at 150.92 MHz; ^{31}P at 242.97 MHz) or on Bruker DPX 300 spectrometer (^{1}H at 300.11 MHz; ^{13}C at 75.03 MHz; ^{31}P at 121.48 MHz) using standard Bruker NMR software. Chemical shifts are reported in ppm, where ^{1}H NMR spectra recorded from samples in $CDCl_3$ were referenced to internal TMS and ^{13}C spectra were referenced to the corresponding solvent signal (77.16 ppm for $CDCl_3$). NMR spectra recorded from samples in other solvents were referenced to residual solvent signals (for CD_3OD 3.31 and 49.00 ppm; for CD_2Cl_2 5.32 and 53.84 ppm; for DMSO-d_6 2.50 and 39.52 ppm; for ^{1}H and ^{13}C NMR, respectively). ^{31}P NMR spectra were referenced according to IUPAC recommendations from a referenced ^{1}H-NMR spectrum. In all 1,1´-disaccharides the NMR signals of the "proximal" GlcN ring is indicated by primes. High-resolution mass spectrometry (HRMS) was carried out on acetonitrile or DCM solutions via LC-TOF MS (Agilent 1200SL HPLC and Agilent 6210 ESI-TOF, Agilent Technologies). Datasets were analysed using Agilent Mass Hunter Software. MALDI-TOF MS was performed in negative-ion mode using a Bruker Autoflex Speed instrument with 6-aza-2-thiothymine (ATT) or 2,5-dihydroxybenzoic acid (DHB) as matrix. Spectra were processed with the manufacturer's software (Bruker Flex analysis 3.3.80) using the SNAP algorithm with a signal/noise threshold of 6 (unsmoothed). Optical rotation was measured on a PerkinElmer 243B polarimeter equipped with a Haake water circulation bath and a Haake D1 immersion circulator for temperature control or an Anton Paar MCP 100 polarimeter featuring integrated Peltier temperature control. All $[\alpha]^D_{20}$ values are reported in units of deg*dm−1 *cm3 *g−1, the corresponding concentrations are reported in g / 100 mL.

(2-Cyanoethoxy)-[2-(benzyloxycarbonylamino)ethoxy]-(diisopropylamino)-phosphine (1)

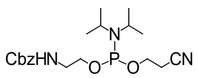

To a stirred solution of 2-Cyanoethyl *N*,*N*,*N*′,*N*′-tetraisopropylphosphorodiamidite (0.995 mmol, 0.3 g) and benzyl N-(2-hydroxyethyl)carbamate (0.6 equiv., 0.663 mmol, 130 mg) in dry DCM (1 mL), 1*H*-Tetrazole (0.45 M in acetonitrile, 0.3 equiv., 0.44 mL) was added at r.t. and the reaction mixture was stirred for 20 min under atmosphere of Ar. The mixture was diluted with DCM (50 mL) and washed with satd. aq. $NaHCO_3$ (3x15 mL), dried over Na_2SO_4, filtered and concentrated. The residue was purified by column chromatography on silica gel (15 g), (hexane – EtOAc, 7:3 buffered with 1% Et_3N), to give **1** (250 mg, 95 %) as colourless oil; R_f = 0.5 (hexane – EtOAc, 7:3); ^{1}H NMR (300 MHz, CD_2Cl_2): δ 7.39-7.30 (5H, Ph), 5.27 (1H, NH), 5.11 (s, 2H, CH_2 Cbz), 3.89-3.56 (m, 6H, OC*H*$_2$CH$_2$NHCbz, OC*H*$_2$CH$_2$CN, 2xNC*H*(CH$_3$)$_2$), 3.44-3.38 (m, 2H, OCH$_2$C*H*$_2$NHCbz), 5.22 (t, 1H, ^3J = 6.3 Hz, OCH$_2$C*H*$_2$CN), 1.22,

1.21, 1.20, 1.18 (4xs, 12H, NCH(CH_3)$_2$); ^{13}C NMR (75 MHz, CD_2Cl_2): δ 156.22 (*C*O Cbz), 137.02 (Cq Ph), 128.41, 127.94, 127.88 (CH Ph), 117.84 (*C*N), 66.40 (CH_2 Cbz), 62.71 (d, $^2J_{P,C}$ = 16.8 Hz, PO*C*H_2CH_2NH), 58.42 (d, $^2J_{P,C}$ = 20.2 Hz, PO*C*H_2CH_2CN), 43.09 (d, $^2J_{P,C}$ = 12.4 Hz, 2xPN*C*H(CH$_3$)$_2$), 42.19 (d, $^3J_{P,C}$ = 7.2 Hz, POCH$_2$*C*H$_2$NH), 24.42, 24.39, 24.32, 24.29 (4xs, 2xNCH(*C*H$_3$)$_2$) 20.35 (d, $^3J_{P,C}$ = 6.2 Hz, POCH$_2$*C*H$_2$CN); ^{31}P NMR (121 MHz, CD_2Cl_2) δ -147.45.

6-*O*-Benzyl-2-deoxy-3-*O*-[(*R*)-3-(benzyloxy)decanoyl]-4-*O*-[(2-benzyloxycarbonylamino)ethoxy]phosphoryl-2-[(*R*)-3-(benzyloxy)tetradecanoylamino]-β-D-glucopyranosyl-(1↔1)-6-*O*-benzyl-3-*O*-[(*R*)-3-(benzyloxy)-decanoyl]-2-deoxy-4-*O*-[(2-benzyloxycarbonylamino)ethoxy]phosphoryl-2-[(*R*)-3-(benzyloxy)tetradecanoyl-amino]-α-D-glucopyranoside (5)

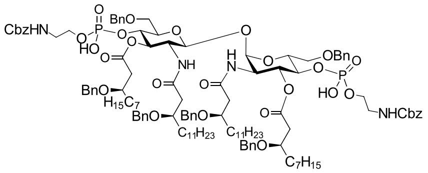

To a stirred solution of **3** (0.03 mmol, 50 mg) in dry DCM (1 mL), a solution of **1** (5 equiv., 0.15 mmol, 59 mg) in dry DCM (1 mL) and 1*H*-Tetrazole (0.45 M in acetonitrile, 5 equiv., 0.33 mL) were added successively at r.t. and the reaction mixture was stirred for 20 min under atmosphere of Ar. The mixture was cooled to -78°C and m-chloroperbenzoic acid (6.5 equiv., 0.2 mmol, 34 mg) in dry DCM (2 mL) was added and the stirring was continued for 2 h at -78°C. A solution of Et_3N (13 equiv., 0.4 mmol, 5 µL) in dry MeOH (1 mL) was added at -78°C and the reaction mixture was stirred while warming up to r.t. The mixture was diluted with EtOAc (20 mL) and washed with 10% aq. $Na_2S_2O_3$ (3x50 mL) and satd. aq. $NaHCO_3$ (3x50 mL), dried over Na_2SO_4, filtered and concentrated. The residue was dissolved in dry DCM – acetonitrile (1:1, 1 ml), then Et_3N (0.2 mL) was added, and reaction mixture was stirred for 40 h at r.t. under atmosphere of Ar. Toluene (5 mL) was added and the mixture was concentrated. The residue was purified by column chromatography on silica gel (6 g), (DCM – MeOH, 10:1 buffered with 0.1% Et_3N), and by size exclusion chromatography on BioRad SX1 (800x5mm), (toluene - CH_2Cl_2, 2:1) to give **5** (48 mg, 73 % over two steps) as white powder. R_f = 0.3 (DCM – MeOH, 10:1); $[\alpha]_D^{20}$= +10 (c 1.0, $CHCl_3$); ^{1}H NMR (600 MHz, $CDCl_3$ – CD_3OD, 1:2) δ 7.23-7.14 (40H, Ph), 6.86 (1H, NH), 6.61 (d, 1H, $^3J_{NH',H2'}$ = 8.9 Hz, NH′), 5.22 (dd, 1H, $^3J_{H2',H3'}$ = $^3J_{H3',H4'}$ = 9.9 Hz, H-3′), 4.95-4.94 (m, 5H, H-3, 2xCH$_2$ Cbz), 4.70 (d, 1H, $^3J_{H1',H2'}$ = 3.2 Hz, H-1′), 4.48-4.35 (m, 9H, H-4′, 4xCH$_2$Ph), 4.28-4.13 (m, 5H, H-2′, H-4, H-5′, 2xCH$_2$Ph), 4.09 (d, 1H, $^3J_{H\text{-}1,H\text{-}2}$ = 7.9 Hz, H-1), 3.84-3.79 (m, 2H, H-2, β^{Myr}-C*H*), 3.75-3.69 (m, 7H, H-6a, 2xβ^{Capr}-C*H*, 2x OC*H*$_2$CH$_2$NHCbz), 3.60-3.59 (m, 2H, H-6′), 3.53-3.50 (m, 2H, H-6b, β^{Myr}-C*H*), 3.39 (m, 1H, H-5), 3.16-3.11 (m, 2H, OCH$_2$C*H*$_2$NHCbz), 3.05-2.97 (m, 2H, OCH$_2$C*H*$_2$NHCbz), 2.85-2.81 (m, 6H, CH$_2$-TEA), 2.66-2.59 (m, 2H, α$^{Myr\ and\ Capr}$-C*H*$_2$a), 2.43-2.34 (m, 3H, α^{Myr}-C*H*$_2$a, α$^{Myr\ and\ Capr}$-C*H*$_2$b), 2.30 (dd, 1H, ^2J = 14.9 Hz, ^3J = 7.6 Hz, α^{Capr}-C*H*$_2$a), 2.21 (dd, 1H, ^2J = 15.0 Hz, ^3J = 3.3 Hz, α^{Myr}-C*H*$_2$b), 2.05 (dd, 1H, ^3J = 14.8 Hz, ^2J = 4.3 Hz, α^{Capr}-C*H*$_2$b), 1.47-1.07 (m, 73H, 32xCH$_2$ Capr and Myr*,* CH$_3$ TEA), 0.81-0.78 (m, 12H, CH$_3$ Capr and Myr*)*; ^{13}C NMR (151 MHz, $CDCl_3$ – CD_3OD, 1:2) δ 172.77, 172.12, 171.86 (4xCO, Capr and Myr), 157.04 (q, 2xCO, Cbz), 139.03, 138.26 (Cq, Ph), 136.58 (Cq, Cbz), 128.34, 128.28, 128.27, 128.16, 128.11, 128.04, 127.98, 127.92, 127.80, 127.63, 127.57, 127.50, 127.40 (*C*H, Ph), 100.62 (C-1), 98.77 (C-1′), 76.77 (βMyr-C*H*), 75.59 (β^{Myr}-C*H*), 75.45 (β^{Capr}-C*H*), 75.35 (β^{Capr}-C*H*), 74.83 (d, $^3J_{P,C}$ = 4.8 Hz, C-5), 73.34 (CH$_2$Ph), 73.27 (CH$_2$Ph), 73.22 (C-3), 72.48 (d, $^2J_{P,C}$ = 5.2 Hz, C-4), 72.22 (C-3′,C-4′), 71.97 (CH$_2$Ph), 71.32 (CH$_2$Ph), 71.20 (d, $^3J_{P,C}$ = 5.3 Hz, C-5′), 71.11 (CH$_2$Ph), 70.24 (CH$_2$Ph), 68.95 (C-6), 67.82 (C-6′), 66.46 (CH$_2$ Cbz), 65.32 (d, $^2J_{P,C}$ = 5.3 Hz, PO*C*H$_2$CH$_2$NHCbz), 65.02 (d, $^2J_{P,C}$ = 5.7 Hz, PO*C*H$_2$CH$_2$NHCbz), 54.07 (C-2), 51.29 (C-2′), 45.74 (CH$_2$-Tea), 41.45, 41.41, 41.25, 41.09 (POCH$_2$*C*H$_2$NHCbz, CH$_2$ Capr and Myr), 38.99, 34.30, 34.25, 34.24, 33.57, 31.84, 31.80, 29.78, 29.68, 29.66, 29.63, 29.61, 29.60, 29.58, 29.29, 29.26, 25.43, 25.20, 25.14, 24.87, 22.56 (CH$_2$ Capr and Myr), 13.81 (CH$_3$ Capr and Myr), 8.18 (CH$_3$ Tea); ^{31}P NMR (243 MHz, $CDCl_3$ – CD_3OD, 1:2) δ -2.59, -2.92; HRMS (ESI) *m/z* calcd. for $[M-2H]^{-2}$ $C_{122}H_{170}N_4O_{27}P_2$ 1092.5842, found 1092.5778.

3-*O*-[(*R*)-3-(hydroxyl)Decanoyl]-2-deoxy-4-*O*-[(2-(N,N-dimethylamino)ethoxy)phosphoryl]-2-[(*R*)-3-(hydroxyl)-tetradecanoylamino]-β-D-glucopyranosyl-(1↔1)-3-*O*-[(*R*)-3-(hydroxyl)decanoyl]-2-deoxy-4-*O*-[2-(*N,N*-dimethylamino)ethoxy-phosphoryl]-2-[(*R*)-3-(hydroxyl)tetradecanoylamino]-α-D-glucopyranoside (6)

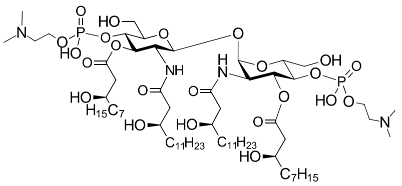

Hydrogenolysis was performed in a pressure-reactor (8.5 bar). A solution of **5** (0.003 mmol, 7 mg) in MeOH - toluene (2:1, 9 mL) was stirred with palladium-black (10 mg) under atmosphere of hydrogen (8.5 bar) for 16 h. The mixture was diluted with MeOH - $CHCl_3$ (2:1, 40 mL), the solids were removed by filtration (membrane filter, regenerated cellulose; 0.45 µm) and the filtrate was concentrated. The residue was purified by size exclusion chromatography on Sephadex LH-20 support (DCM – MeOH, 1:2) to give **6** (2.7 mg, 60 %) as white amorphous solid. R_f = 0.3 ($CHCl_3$ - MeOH – water, 5:3:0.5 with 0.1 % Et_3N); ^{1}H NMR (600 MHz, $CDCl_3$ – CD_3OD, 1:3 D) δ 7.78 (d, 1H, $^3J_{NH,H2}$ = 9.1 Hz, NH), 5.22 (dd, 1H, $^3J_{H2',H3'}$ = 10.7 Hz, $^3J_{H3',H4'}$ = 9.1 Hz, H-3´), 5.16 (dd, 1H, $^3J_{H2,H3}$ = $^3J_{H3,H4}$ = 9.8 Hz, H-3), 5.04 (d, 1H, $^3J_{H1',H2'}$ = 3.6 Hz, H-1´), 4.69 (d, 1H, $^3J_{H1,H2}$ = 8.5 Hz, H-1), 4.26-4.22 (m, 2H, H-4, H-4´), 4.29 (dd, 1H, $^3J_{H2',H3'}$ = 10.9 Hz, $^3J_{H1',H2'}$ = 3.6 Hz, H-2´), 4.06-3.94 (m, 10H, H-2, H-5´, 4xβ$^{\text{Myr and Capr}}$-C*H*, 2xPOC*H*$_2$CH$_2$NH$_2$), 3.87-3.78 (m, 4H, H-6, H-6´), 3.45 (m, 1H, H-5), 3.18-3.10 (m, 4H, 2xPOCH$_2$C*H*$_2$NH$_2$), 2.83-2.70 (m, 12H, 4xCH$_3$ NMe), 2.54-2.20 (m, 8H, α$^{\text{Capr and Myr}}$-C*H*$_2$), 1.43-1.27 (m, 64H, 32xCH$_2$ Myr and Capr), 0.89-0.87 (m, 12H, 4xCH$_3$- Myr and Capr); ^{13}C NMR (151 MHz, $CDCl_3$ – CD_3OD, 1:3) δ 101.04 (C-1), 98.78 (C-1´), 76.02 (C-5), 74.03 (C-3), 72.22 (C-5´), 72.01 (C-3´), 71.66 (C-4, C-4´), 68.52, 68.52, 67.68, 67.68 (4x4xβ$^{\text{Myr and Capr}}$-*C*H), 61.78, 61.78 (2x PO*C*H$_2$CH$_2$NH$_2$), 60.74, 60.74 (C-6, C-6´), 53.86 (C-2), 51.85 (C-2´), 44.67, 42.98 (CH$_2$ Capr and Myr), 42.6 (CH$_3$ NMe), 42.3 (CH$_2$ Capr and Myr), 40.37 (2xPOCH$_2$*C*H$_2$NH$_2$), 37.52, 36.89, 31.81, 29.47, 25.33, 22.39 (CH$_2$ Capr and Myr), 13.30 (CH$_3$ Capr and Myr); All ^{13}C shifts except for those arising from carbonyl carbons were gained from HSQC. Carbonyl ^{13}C shifts were obtained from HMBC; ^{31}P NMR (243 MHz, $CDCl_3$ – CD_3OD, 1:3) δ 0.00, -0.09.

6-*O*-Benzyl-2-deoxy-3-*O*-[(*R*)-3-(benzyloxy)decanoyl]-4-*O*-[(2-aminoethoxy)phosphoryl]-2-[(*R*)-3-(benzyloxy)-tetradecanoylamino]-β-D-glucopyranosyl-(1↔1)-6-*O*-benzyl-3-*O*-[(*R*)-3-(benzyloxy)decanoyl]-2-deoxy-4-*O*-[(2-aminoethoxy)phosphoryl]-2-[(*R*)-3-(benzyloxy)tetradecanoylamino]-α-D-glucopyranoside (8)

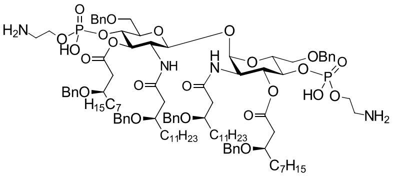

To a stirred solution of **3** (0.03 mmol, 50 mg) in dry DCM (1 mL), a solution of **2** (5 equiv., 0.15 mmol, 59 mg) in dry DCM (1 mL) and 1*H*-Tetrazole (0.45 M in acetonitrile, 5 equiv., 0.33 mL) were added successively at r.t. and the reaction mixture was stirred for 20 min. under atmosphere of Ar. The reaction mixture was cooled to -78°C and m-chloroperbenzoic acid (6.5 equiv., 0.2 mmol, 34 mg) in dry DCM (2 mL) was added and stirred for 2 h at -78°C. A solution of Et_3N (13 equiv., 0.4 mmol, 5 µL) in dry MeOH (1 mL) was added at -78°C and the reaction mixture was stirred while warming up to r.t. EtOAc (20 mL) was added to the reaction mixture and then extracted with 10% aq. $Na_2S_2O_3$ (3x50 mL) and satd. aq. $NaHCO_3$ (3x50 mL) and the organic phase was dried over Na_2SO_4, filtrated and concentrated. The residue was dissolved in a mixture of dry DCM - acetonitrile (1:1, 1 ml), Et_3N (0.2 mL) was added at r.t. and reaction mixture was stirred for 40 h. Toluene (5 mL) was added and the reaction mixture was concentrated. The residue was purified by column chromatography on silica gel (6 g), (DCM – MeOH, 10:1 → 7:3 with 0.1% Et_3N), appropriate fractions were collected and passed through a gel bed of BioRad SX1 column (800x 5mm), (toluene - CH_2Cl_2, 2:1), to give **8** (38 mg, 67 %) as white powder. R_f = 0.4 (DCM – MeOH, 7:3 with 0.1 % Et_3N); $[\alpha]_D^{20}$= +12 (c 1.0, $CHCl_3$); ^{1}H NMR (600 MHz,

$CDCl_3 – CD_3OD$ = 1:3) δ 7.29-7.17 (30H, Ph), 6.76 (d, 1H, $^3J_{NH',H2'}$ = 9.4 Hz, NH´), 5.25 (dd, 1H, $^3J_{H2',H3'}$ = $^3J_{H3',H4'}$ = 9.9 Hz, H-3´), 5.07 (dd, 1H, $^3J_{H2,H3}$ = $^3J_{H3,H4}$ = 9.8 Hz, H-3), 4.83 (d, 1H, $^3J_{H-1',H-2'}$ = 3.2 Hz, H-1´), 4.50-4.41 (m, 10H, H-4´, CH_2Ph, CH_2Ph), 4.37 (d, 1H, $^3J_{H-1,H-2}$ = 8.5 Hz, H-1), 4.34-4.18 (m, 3H CH_2Ph, CHa CH_2Ph), 4.24-4.18 (m, 3H, H-2´, H-4, H-5´), 3.89-3.66 (m, 11H, H-2, H-6a, H-6´, OCH_2CH_2NHCbz, $\beta^{Myr\ and\ Capr}$-C*H*), 3.59-3.56 (m, 2H, Hb-6, β^{Capr}-C*H*), 3.49 (m, 1H, H-5), 2.69 (dd, 1H, ^2J = 17.2 Hz, ^3J = 8.17 Hz, $\alpha^{Myr\ and\ Capr}$-CH_2a), 2.63 (dd, 1H, ^2J = 17.23 Hz, ^3J = 8.27 Hz, $\alpha^{Myr\ and\ Capr}$-CH_2a), 2.51-2.37 (m, 8H, $\alpha^{Myr\ and\ Capr}$-CH_2, 2xOCH_2CH_2NHCbz), 2.29 (dd, 1H, ^2J = 14.9 Hz, ^3J = 3.2 Hz, $\alpha^{Myr\ and\ Capr}$-CH_2b), 2.11 (dd, 1H, ^2J = 14.7 Hz, ^3J = 4.6 Hz, $\alpha^{Myr\ and\ Capr}$-CH_2b), 1.48-1.20 (m, 64H, 32xCH_2 Capr and Myr), 0.85-0.82 (m. 12H, CH_3 Capr and Myr*)*; ^{13}C NMR (151 MHz, $CDCl_3 – CD_3OD$ = 1:3) δ 173.68, 173.05, 172.97, 172.62 (4xCO, Capr and Myr), 139.73, 139.47, 139.31, 139.16 (Cq, Ph), 129.26, 129.21, 129.15, 129.09, 129.07, 129.06, 128.88, 128.80, 128.62, 128.50, 128.41, 128.36, 128.21, 128.12 (CH, Ph), 100.97 (C-1), 99.36 (C-1´), 77.40 (β^{Myr}-*C*H), 76.62 (β^{Myr}-*C*H), 76.59 (β^{Capr}-*C*H), 76.52 (β^{Capr}-*C*H), 76.02 (d, $^3J_{P,C}$ = 6.81 Hz, C-5), 74.39 (C-3), 74.19 (CH_2Ph), 74.10 (CH_2Ph), 73.54 (C-3´), 73.25 (d, $^2J_{P,C}$ = 6.2 Hz, C-4), 72.89 (d, $^3J_{P,C}$ = 5.5 Hz, C-5´), 72.60 (CH_2Ph), 72.549 (CH_2 Ph), 72.40 (d, $^3J_{P,C}$ = 5.5 Hz, C-4´), 72.27 (CH_2Ph), 70.85 (CH_2Ph), 70.17 (C-6), 69.29 (C-6´), 62.24 (d, $^2J_{P,C}$ = 5.7 Hz, $POCH_2CH_2NHCbz$), 65.02 (d, $^2J_{P,C}$ = 5.7 Hz, $POCH_2CH_2NHCbz$), 55.32 (C-2), 52.42 (C-2´), 42.03, 41.86 (CH_2 Capr and Myr), 41.12 ($POCH_2CH_2NHCbz$), 39.76, 39.76, 35.06, 34.92, 34.59, 32.72, 32.65, 30.61, 30.56, 30.47, 30.40, 30.14, 26.23, 25.99, 25.86, 23.39 (CH_2 Capr and Myr), 14.43, 14.39 (CH_3 Capr and Myr); ^{31}P NMR (243 MHz, $CDCl_3 – CD_3OD$ = 1:3) δ -1.93, -2.08; HRMS (ESI) *m/z* calcd. for $[M+2H]^{+2}$ $C_{106}H_{162}N_4O_{23}P_2$ 960.5474, found 960.5559.

6-*O*-Benzyl-2-deoxy-3-*O*-[(*R*)-3-(benzyloxy)decanoyl]-4-*O*-hydrogenphosphonate-2-[(*R*)-3-(benzyloxy)-tetradecanoylamino]-β-D-glucopyranosyl-(1↔1)-6-*O*-benzyl-3-*O*-[(*R*)-3-(benzyloxy)decanoyl]-2-deoxy-4-*O*-hydrogenphosphonate-2-[(*R*)-3-(benzyloxy)tetradecanoylamino]-α-D-glucopyranoside triethylammonium salt (9)

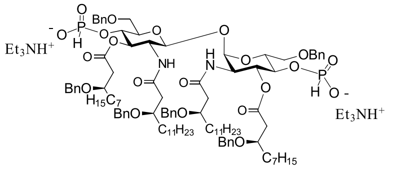

To a stirred solution of **3** (0.54 mmol, 0.1 g) in dry DCM (0.5 mL), dry pyridine (0.5 mL) and 2-chloro-1,3,2-benzodioxaphosphorin-4-one (5 equiv., 0.27 mmol, 54 mg) were successively added and the solution was stirred at r.t. for 16 h under atmosphere of Ar. Then a solution of Et_3N (0.5 mL) in water (0.5 mL) was added and the reaction mixture was stirred at r.t. for 20 min. The mixture was diluted with DCM (10 mL), washed with aq. Et_3N (pH 10, 2x3 mL), filtered over cotton, diluted with toluene (20 mL) and concentrated. The residue was purified by column chromatography on silica gel (10 g), (EtOAc – MeOH, 9:1 → 7:3, with 0.1% Et_3N), to give **9** (78 mg, 85 %) as colourless sirup. $[\alpha]_D^{20}$= +13 (c 1.0, $CHCl_3$); ^1H NMR (600 MHz, $CDCl_3$ – CD3OD, 3:1) δ 7.25-7.16 (31H, Ph, 0.5H PH and 0.5H P´H), 6.84 (m, 1H, NH), 6.57 (m, 1H, NH´), 6.14 (d, 0.5H, $J_{P,H}$ = 645 Hz, PH), 6.11 (d, 0.5H, $J_{P,H}$ = 652 Hz, P´H), 5.19 (dd, 1H, $^3J_{H2',H3'}$ = $^3J_{H3',H4'}$ = 9.9 Hz, H-3´), 4.91 (dd, 1H, $^3J_{H2,H3}$ = $^3J_{H3,H4}$ = 9.8 Hz, H-3), 4.71 (d, 1H, $^3J_{H-1',H-2'}$ = 3.7 Hz, H-1´), 4.50-4.41 (m, 17H, H-1, H-2´, H-4, H-4´, H-5´, CH_2Ph, CH_2Ph), 3.85-3.80 (m, 2H, H-2, $\beta^{Capr\ and\ Myr}$-C*H*), 3.75-3.71 (m, 3H, Ha-6, β^{Capr}-C*H*), 3.60 (m, 1H, H-6´a), 3.53-3.49 (m, 3H, H-6b, H-6´b, β^{Myr}-C*H*), 3.39 (m, 1H, H-5), 2.87-2.84 (m, 8H, CH_2-Tea), 2.62-2.54 (m, 2H, $\alpha^{Capr\ and\ Myr}$-CH_2a), 2.43-2.28 (m, 4H, $\alpha^{Capr\ and\ Myr}$-CH_2), 2.21-2.19 (dd, 1H, $\alpha^{Capr\ and\ Myr}$-CH_2b), 2.04 (dd, 1H, ^2J = 14.7 Hz, ^3J = 4.0 Hz, $\alpha^{Capr\ and\ Myr}$-CH_2b), 1.48-1.20 (m, 64H, 32xCH_2 Capr and Myr), 0.85-0.82 (m. 12H, CH_3 Capr and Myr*)*; ^{13}C NMR (151 MHz,$CDCl_3 – CD_3OD$, 3:1) δ173.04, 172.39, 172.03, 171.89(4xCO, Capr and Myr), 139.33, 138.46, 138.62, 138.50 (q, Ph), 128.75, 128.69, 128.66, 128.56, 128.54, 128.36, 128.29, 128.23, 128.21, 128.04, 128.02, 127.91, 127.84 (CH, Ph), 100.95 (C-1), 99.18 (C-1´), 77.19 (β^{Myr}-CH), 75.97 (β^{Myr}-CH), 75.73 (β^{Capr}-CH), 75.61 (β^{Capr}-CH), 75.11 (d, $^3J_{P,C}$ = 5.5 Hz, C-5), 73.85 (C-3), 73.76 (CH_2Ph), 73.66 (CH_2Ph), 73.02 (C-3´), 72.37 (CH_2Ph), 71.68 (d, $^3J_{P,C}$ = 5.5 Hz, C-5´), 71.64 (CH_2Ph), 71.41 (CH_2Ph), 71.01 (d, $^2J_{P,C}$ = 6.2 Hz, C-4), 70.90 (d, $^2J_{P,C}$ = 6.3 Hz, C-4´), 70.69 (CH_2Ph), 69.56 (C-6), 68.48 (C-6´), 54.44 (C-2), 51.66 (C-2´), 46.12 (CH_2 Tea), 41.72, 41.51, 39.73, 34.74, 34.02, 32.29, 32.11, 30.19, 29.95, 29.75, 29.63, 25.82, 25.55, 25.31, 23.02 (CH_2 Capr and Myr), 14.24(CH_3 Capr and Myr), 8.61 (CH_3 Tea); ^{31}P NMR (243 MHz,$CDCl_3 – CD_3OD$, 3:1) δ 3.61 (dd, $^1J_{P,H}$ = 651.1 Hz, $^3J_{P,H4}$ = 8.4 Hz), 3.16 (dd, $^1J_{P,H}$= 646.5 Hz, $^3J_{P,H4'}$ = 10.9 Hz); HRMS (ESI) *m/z* calcd. for $[M - 2H]^{-2}$ $C_{102}H_{148}N_2O_{21}P_2$ 899.5024, found 899.5028.

6-*O*-Benzyl-2-deoxy-3-*O*-[(*R*)-3-(benzyloxy)decanoyl]-4-*O*-[(2-tritylaminoethoxy)phosphoryl]-2-[(*R*)-3-(benzyloxy)tetradecanoylamino]-β-D-glucopyranosyl-(1↔1)-6-*O*-benzyl-3-*O*-[(*R*)-3-(benzyloxy)decanoyl]-2-deoxy-4-*O*-[(2-tritylaminoethoxy)phosphoryl]-2-[(*R*)-3-(benzyloxy)tetradecanoylamino]-α-D-glucopyranoside (11)

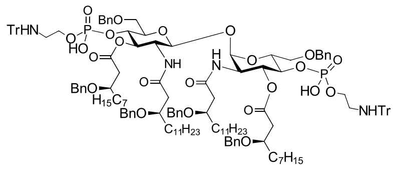

To a stirred solution of **9** (0.05 mmol, 95 mg) in a ACN-DCM (1:1, 2 mL), 2-(tritylamino)ethanol (5 equiv., 0.25 mmol, 75 mg), followed by 3-Nitro-l,2,4-triazol-l-yl-tris(pyrrolidin-1-yl)phosphonium hexafluorophosphate (PyNTP, 10 equiv., 0.5 mmol, 250 mg) and 2,6 lutidine (24 equiv., 1.2 mmol, 0.14 mL) were added at r.t. and reaction mixture was stirred for 4 h in the atmosphere of Ar. A solution of I_2 (1 equiv., 0.05 mmol, 12 mg) in pyridine - water (30:1, 2 mL) was added at 0 °C and the reaction mixture was stirred for 4 h at 0 °C. The mixture was diluted with DCM (15 mL), and washed with 10% aq. $Na_2S_2O_3$ (3x5 mL) and satd. aq. $NaHCO_3$ (3x5 mL), dried over Na_2SO_4, filtered over cotton and concentrated. The residue was purified by column chromatography on silica gel (15 g), (EtOAc → EtOAc – MeOH, 10:3 with 0.1% Et_3N), to give **11** (80 mg, 75 %) as colourless sirup. R_f = 0.2 (EtOAc – MeOH, 10:3); $[\alpha]_D^{20}$= +7 (c 1.0, $CHCl_3$); ^1H NMR (600 MHz, $CDCl_3$ – CD_3OD, 1:3) δ 7.31-6.96 (60H, Ph), 6.83 (d, 1H, $^3J_{NH',H2}$ = 9.3 Hz, NH′), 5.27 (dd, 1H, $^3J_{H2',H3'}$ = $^3J_{H3',H4'}$ = 9.8 Hz, H-3′), 5.08 (dd, 1H, $^3J_{H2,H3}$ = $^3J_{H3,H4}$ = 9.6 Hz, H-3), 4.83 (d, 1H, $^3J_{H-1',H-2'}$ = 2.9 Hz, H-1′), 4.50 (m, 1H, H-4′), 4.41-4.19 (m, 14H, H-1, H-2′, H-4, H-5′, 5xCH_2Ph), 3.90 (dd, 2H, $^3J_{H2,H3}$ = $^3J_{H3,H4}$ = 9,4 Hz, H-2), 3.80-3.60 (m, 10H, H-6a, H-6′, $\beta^{Myr\ and\ Capr}$-C*H*, 2xOC$H_2$$CH_2$NHTr), 3.55-3.49 (m, 3H, H-5, H-6b, $\beta^{Myr\ and\ Capr}$-C*H*), 2.59-2.22 (m, 11H, $\alpha^{Capr\ and\ Myr}$-CH_2, 2xOCH_2CH_2NHTr), 2.07 (dd, 1H, ^2J = 14.7 Hz, ^3J = 4.8 Hz, $\alpha^{Myr\ and\ Capr}$-CH_2b*)*, 1.45-1.16 (m, 64H, 32x CH_2 Capr and Myr), 0.81-0.78 (m, 12H, CH_3 Capr and Myr); ^{13}C NMR (151 MHz, $CDCl_3$ – CD_3OD, 1:3) δ 173.91, 173.32, 173.00, 172.64 (4xCO, Capr and Myr), 139.98, 139.71, 139.58 (q, Ph), 129.71, 129.63, 129.18, 128.98, 128.95, 128.83, 128.76, 128.58, 128.56, 128.51, 129.25, 128.14 (CH, Ph), 101.08 (C-1), 99.60 (C-1′), 77.53 (β^{Myr}-*C*H), 76.76 (β^{Myr}-*C*H), 76.35 (β^{Capr}-*C*H), 76.26 (β^{Capr}-*C*H), 76.10 (d, $^3J_{P,C}$ = 6.81 Hz, C-5), 74.47 (C-3), 74.16 (CH_2Ph), 73.75 (C-5′), 73.52 (C-3′), 73.42 (d, $^2J_{P,C}$ = 6.81 C-4′), 72.60 (CH_2Ph), 72.50 (d, $^2J_{P,C}$ = 6.0 C-4), 72.21 (CH_2Ph), 71.91 (CH_2Ph), 71.10 (CH_2Ph), 70.32 (C-6), 69.46 (C-6′), 61.84 (PO*C*$H_2$$CH_2$NHTr), 61.75 (PO*C*$H_2$$CH_2$NHTr), 55.56 (C-2), 52.75 (C-2′), 47.71 (POCH_2*C*H_2NHTr), 47.53 (POCH_2*C*H_2NHTr), 42.25, 42.03, 40.09, 40.02, 35.21, 35.09, 35.04, 33.81, 32.84, 30.71, 30.62, 30.26, 26.33, 26.15, 26.10, 25.96, 23.52 (CH_2 Capr and Myr), 14.49, 14.46 (CH_3 Capr and Myr); ^{31}P NMR (243 MHz, CD_2Cl_2 –CD_3OD, 1:3) δ 1.47, 1.27; HRMS (ESI) *m/z* calcd. for $[M-H]^-$ $C_{144}H_{187}N_4O_{23}P_2$ 2402.3061, found 2402.3093.

6-*O*-Benzyl-2-deoxy-3-*O*-[(*R*)-3-(benzyloxy)decanoyl]-4-*O*-[(2-azidoethoxy)phosphoryl]-2-[(*R*)-3-(benzyloxy)tetradecanoylamino]-β-D-glucopyranosyl-(1↔1)-6-*O*-benzyl-3-*O*-[(*R*)-3-(benzyloxy)decanoyl]-2-deoxy-4-*O*-[(2-azidoethoxy)phosphoryl]-2-[(*R*)-3-(benzyloxy)tetradecanoylamino]-α-D-glucopyranoside (12)

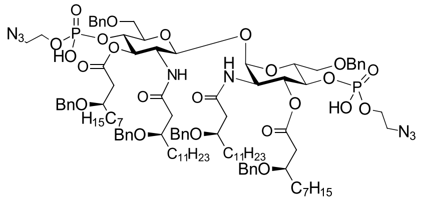

To a stirred solution of **9** (0.016 mmol, 30 mg) in a mixture of dry ACN-DCM (4:1, 2.5 mL), 2-azidoethanol (4 equiv., 0.063 mmol, 5 mg), 3-nitro-l,2,4-triazol-l-yl-tris(pyrrolidin-1-yl)phosphonium hexafluorophosphate (PyNTP) (8 equiv., 0.126 mmol, 63 mg) and 2,6-lutidine (24 equiv., 0.38 mmol, 44 µL) were added successively at r.t. and the reaction mixture was stirred for 8 h under atmosphere of Ar. A solution of I_2 (1 equiv., 0.016 mmol, 3 mg) in pyridine-water (30:1, 1.5 mL) was added at 0 °C and the reaction mixture was stirred for 4 h. The mixture was diluted with DCM (15 mL) and washed with a 10% aq. sol. of $Na_2S_2O_3$ (3x5 mL) and

satd. aq. $NaHCO_3$ (3x5 mL), dried over Na_2SO_4, filtered over cotton and concentrated. The residue was purified by column chromatography on silica gel (5 g), (EtOAc → EtOAc – MeOH, 10:3 with 0.1% Et_3N) to give **12** (20 mg, 64 %) as colourless sirup. R_f = 0.15 (EtOAc – MeOH, 10:3); $[\alpha]_D^{20}$= +98 (c 1.0, $CHCl_3$); ^{1}H NMR (600 MHz, $CDCl_3$ –CD_3OD, 1:3) δ 7.26-7.15 (30H, Ph), 6.76 (d, 1H, NH´), 5.21 (dd, 1H, $^3J_{H2´,H3´}$ = $^3J_{H3´,H4´}$ = 9.9 Hz, H-3´), 4.99 (dd, 1H, $^3J_{H2,H3}$ = $^3J_{H3,H4}$ = 9.8 Hz, H-3), 4.75 (d, 1H, $^3J_{H-1´,H-2´}$ = 3.3 Hz, H-1´), 4.49-4.34 (m, 10H, H-4´, 2xCH_2Ph, 2xCH_2Ph Capr), 4.27-4.14 (m, 7H, H-1, H-2´, H-4, H-5´, CH_2-Ph, CH_2Ph), 3.83-3.73 (m, 9H, H-2, Ha-6, 2xO$CH_2CH_2N_3$, 3x$\beta^{Myr\ And\ Capr}$-C*H*), 3.66-3.59 (m, 2H, H-6´), 3.56-3.51 (m, 2H, H-6b, 3x$\beta^{Myr\ And\ Capr}$-C*H*), 3.44 (m, 1H, H-5), 3.17-3.14 (m, 4H, 2xO$CH_2CH_2N_3$), 3.01-2.97 (m, 6H, CH_2 Tea), 2.73-2.66 (m, 2H, $\alpha^{Capr\ and\ Myr}$-CH_2a), 2.46-2.42 (m, 2H, $\alpha^{Capr\ and\ Myr}$-CH_2b), 2.38 (m, 1H, $\alpha^{Capr\ and\ Myr}$-CH_2a), 2.33 (m, 1H, $\alpha^{Capr\ and\ Myr}$-CH_2a), 2.21 (dd, 1H, ^2J = 15.1 Hz, ^3J = 3.4 Hz, $\alpha^{Capr\ and\ Myr}$--CH_2b), 2.04 (dd, 1H, ^2J = 14.6 Hz, ^3J = 4.1 Hz, $\alpha^{Capr\ and\ Myr}$-CH_2b), 1.45-1.16 (m, 73H, 32xCH_2 Capr and Myr, CH_3 TEA), 0.81-0.78 (m. 12H, CH_3 Capr and Myr*)*; ^{13}C NMR (151 MHz,$CDCl_3$–CD_3OD, 1:3) δ 173.88, 173.25, 172.79, 172.65 (4xCO, Capr and Myr), 139.91, 139.69, 139.65, 139.57, 139.49, 139.40 (Cq, Ph), 129.21, 129.19, 129.06, 129.04, 128.94, 128.93, 128.88 (CH, Ph), 101.31 (C-1), 99.58 (C-1´), 77.65 (β^{Myr}-*C*H), 76.67 (β^{Myr}-*C*H), 76.25 (2x β^{Capr}-*C*H), 76.00 (d, $^3J_{P,C}$ = 6.81 Hz, C-5), 74.37 (C-3), 74.23 (CH_2Ph), 74.18 (CH_2Ph), 73.54 (C-3´, C-4, C-4´), 72.88 (CH_2Ph), 72.24 (C-5´), 72.03 (CH_2Ph), 71.76 (CH_2Ph), 71.26 (CH_2Ph), 70.32 (C-6), 69.11 (C-6´), 66.02 (PO*C*$H_2CH_2N_3$), 65.58 (PO*C*$H_2CH_2N_3$), 55.34 (C-2), 52.43 (C-2´), 52.16 (d, $^3J_{P,C}$ = 7.6 Hz, POCH_2*C*H_2N_3), 51.97 (d, $^3J_{P,C}$ = 7.6 Hz, POCH_2*C*H_2N_3), 47.21 (CH_2 Tea), 42.27, 42.17, 39.35, 35.32, 34.69, 32.85, 32.81, 32.77, 30.75, 30.66, 30.61, 30.55, 30.29, 30.25, 26.32, 26.18, 26.07, 25.86, 23.52 (CH_2 Capr and Myr), 14.54, 14.51 (CH_3 Capr and Myr), 9.14 (CH_3 Tea); ^{31}P NMR (243 MHz,$CDCl_3$ –CD_3OD, 1:3) δ -1.84, -2.28; HRMS (ESI) *m/z* calcd. for $[M - H]^-$ $C_{106}H_{155}N_8O_{23}P_2$ 1970.0680, found 1970.0700.

(*R*)-3-(naphthalen-2-ylmethoxy)dodecanoic acid (15)

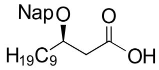

To a cooled (0°C) solution of methyl (R)-3-hydroxydodecanoate (4.491 mmol, 1.03 g) and 2-naphthaldehyde (3 eq., 13.478 mmol, 2.105 g) in THF (10 mL) was added hexamethyldisiloxane (6 eq., 26.95 mmol, 5.7 mL). After stirring for 15 min, TMS-OTf (0.8 eq., 3.59 mmol, 0.65 mL,) was added and stirring was continued for 15 min. Et_3SiH (3.5 eq., 15.71 mmol, 2.51 mL) was added and the reaction was stirred for 3 h at 0 °C. Reaction mixture was diluted with DCM (50 mL) and extracted with $NaHCO_3$ (3x20 mL) and brine (3x20 mL) after which the organic layer was dried with MgSO4, filtered and concentrated in vacuo. Crude was recrystallized using hexane – EtOAc, 20: 1. To a solution of the methyl ester in THF (15 mL) was added a solution of LiOH (6 eq., 29.84 mmol, 0.72 g) in water (3 mL). The resulting mixture was stirred vigorously for 4 h after which it was quenched by addition of 1.0 M HCl (aq.) until pH ≈ 2. DCM (60 mL) was added and the mixture was extracted with $NaHCO_3$ (3x20 mL) and brine (3x20 mL) after which the organic layer was dried with MgSO4, filtered and concentrated in vacuo. The residue was purified by column chromatography on silica gel (hexane – EtOAc, 20: 1, with 1% AcOH) afforded compound **15** as a clear yellow oil (1.27 g, 80%) Rf = 0.2 (Hex - EtOAc, 7:1 with 1% AcOH); ^{1}H NMR (300 MHz, $CDCl_3$): 7.84-7.44 (7H, Arom), 4.74 (s, 2H, CH_2 Nap), 3.94 (m, 1H, β^{Lau}-C*H*), 2.6 (dd, 1H, ^2J = 14.9 Hz, ^3J = 8.3 Hz, α^{Lau}- CH_2a), 2.58 (dd, 1H, ^2J = 14.9 Hz, ^3J = 7.9 Hz, α^{Lau}-CH_2a), 1.75-1.26 (m, 16H, 8xCH_2 Lau), 0.90 (t, 3H, CH_3 Lau); ^{13}C NMR (75 MHz, $CDCl_3$): d 176.63 (C=O), 135.6, 133.29, 133.04 (Cq Nap), 128.19, 127.91, 127.68, 126.55, 126.07, 125.87 (CH Nap), 75.81 (β^{Laur} -*C*H), 71.68 (CH_2 (Nap), 39.48 (CH2COOH), 34.1 (CH2CHCH2COO), 39.48, 34.19, 31.93, 29.65, 29.58, 29.36, 25.15, 22.70 (CH_2 Lau), 14.13 (CH_3 Lau).

4,6-*O*-Benzylidene-3-*O*-*tert*-butyldimethylsilyl-2-deoxy-2-[(*R*)-3-(naphthalen-2-ylmethoxy)tetradecanoylamino]-β-D-glucopyranosyl-(1↔1)-4,6-*O*-benzylidene-3-*O*-*tert*-butyldimethylsilyl-2-deoxy-2-[(*R*)-3-(naphthalen-2-ylmethoxy)tetradecanoylamino]-α-D-glucopyranoside (16)

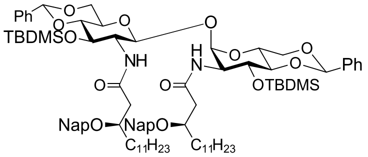

To a stirred solution of 1**3** (1.03 mmol, 0.77 g) in dry DMF (1 mL), a solution of **14** (2.8 equiv., 2.89 mmol, 1.11 g), *N*,*N*-diisopropylethylamine (3.3 equiv., 3.4 mmol, 0.531 mL) and HATU (3 equiv., 3.09 mmol, 1.17 g) in dry DMF (5 mL) was added at r.t. and the reaction mixture was stirred at r.t. for 3 h under atmosphere of Ar. The mixture was diluted with EtOAc (50 mL), and washed with satd. aq. NH_4Cl (3 x15 mL) and brine (3x15 mL), dried over Na_2SO_4, filtered over cotton, and concentrated. The residue was purified by column chromatography on silica gel (70 g), (toluene – acetone, 20:1 → 15:1) to give **16** (1.4 g, 88%) as colorless sirup. R_f = 0.15 (toluene – acetone, 15: 1); $[\alpha]_D^{20}$= +140 (c 1.0, $CHCl_3$); ^{1}H NMR (300 MHz, $CDCl_3$) δ 7.90-7.02 (24H, Aromatic), 6.65 (d, 1H, $^3J_{NH,H2}$ = 9.0 Hz, NH), 6.58 (d, 1H, $^3J_{NH',H2'}$ = 9.7 Hz, NH′), 5.54 (s, 1H, C*H*Ph), 5.25 (s, 1H, C*H*Ph), 4.82 (d, 1H, $^3J_{H1',H2'}$ = 3.5 Hz, H-1′), 4.53 (ddd, 1H, $^3J_{H2',H3'}$ = $^3J_{H2',NH'}$ = 9.7 Hz, $^3J_{H1',H2'}$ = 3.5 Hz, H-2′), 4.26-4.21 (m, 2H, β^{Myr}-C*H*, CHa CH_2 Nap), 4.13-3.99 (m, 2H, H-5′, H-6a′), 3.95-3.89 (m, 2H, H-3′, H-6a), 3.79 (dd, 1H $^2J_{H6a,H6b}$ = $^3J_{H5,H6b}$ = 9.6 Hz, H-6b′), 3.69-3.22 (m, 6H, H-1, H-2, H-4′, β^{Myr}-C*H*, H-6b), 3.11 (dd, 1H, $^3J_{H3,H4}$ = $^3J_{H4,H5}$ = 9.1 Hz, H-4), 2.67-2.39 (m, 4H, H-3, α^{Myr}-CH_2), 2.20 (m, 1H, α^{Myr}-CH_2), 2.03 (m, 1H, H-5), 1.31-1.23 (m, 40H, 20xCH_2 Myr), 0.93-0.88 (m, 6H, 2xCH_3 Myr), 0.82 (s, 9H, 3xCH_3, TBDMS), 0.70 (s, 9H, 3xCH_3, TBDMS), -0.01, -0.03, -0.26 and -0.30 (4s, 12H, 4xCH_3, TBDMS); ^{13}C NMR (75 MHz, $CDCl_3$) δ 173.15, 171.64 (2xCO, Myr), 137.46, 137.36, 137.32, 135.40, 133.45, 133.27, 133.19, 133.18 (q, Aromatic), 129.19, 129.17, 129.10, 128.85, 128.36, 128.25, 128.20, 128.04, 127.99, 127.93, 127.92, 127.90, 127.80, 127.42, 127.24, 126.85, 126.78, 126.69, 126.53, 126.48, 126.34, 126.29, 126.16, 125.43 (*C*H, Aromatic), 102.32 (C-1), 102.07, 101.72 (2x*C*HPh), 101.25 (C-1′), 82.64 (C-4′), 80.77 (C-4), 78.30 (β^{Myr}-*C*H), 77.36 (β^{Myr}-*C*H), 73.91 (*C*H_2 Nap), 72.92 (C-3), 71.27 (C-3′), 71.17 (*C*H_2 Nap), 68.89 (C-6′), 68.45 (C-6), 64.99 (C-5), 63.62 (C-5′), 56.51 (C-2′), 53.69 (C-2), 42.40, 40.82, 35.29, 33.05, 32.11, 32.09, 30.17, 30.07, 29.89, 29.87, 29.84, 29.81, 29.55, 29.54, 26.06 (CH_2 Myr), 25.84, 25.72 (6xCH_3, TBDMS), 22.85 (CH_2 Myr), 21.59 (CH_3 Myr), 18.22, 18.01 (2xCq, TBDMS), -4.05, -4.64 and -4.90 (4xCH_3, TBDMS); HRMS (ESI) *m/z* calcd. for $[M+H]^+$ $C_{88}H_{128}N_2O_{13}Si_2$ 1477.9033, found 1477.9033.

4,6-*O*-Benzylidene-2-deoxy-2-[(R)-3-(naphthalen-2-ylmethoxy)tetradecanoylamino]-β-D-glucopyranosyl-(1↔1)-4,6-*O*-benzylidene-2-deoxy-2-[(R)-3-(naphthalen-2-ylmethoxy)tetradecanoylamino]-α-D-glucopyranoside (17)

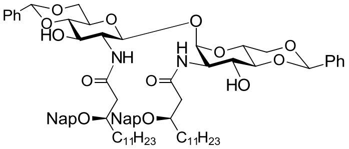

To a stirred solution of **16** (0.95 mmol, 1.4 g) in dry THF (5 mL) in a Teflon tube, TBAF (1 M in THF, 5 mL) was added at r.t. and the reaction mixture was stirred for 1 h under atmosphere of Ar. The reaction mixture was diluted with EtOAc (30 mL) and washed with satd. aq. $NaHCO_3$ (3x10 mL) and brine (3x10 mL), dried over Na_2SO_4, filtered over cotton, and concentrated. The residue was purified by column chromatography on silica gel (200 g), (toluene – EtOAc, 2:1 → 1:1) to give **17** (1 g, 85%) as white foam. R_f = 0.4 (toluene – EtOAc, 2:1.5); $[\alpha]_D^{20}$= +140 (c 1.0, $CHCl_3$); ^{1}H NMR (300 MHz, $CDCl_3$) δ 7.76-7.18 (24H, Arom.), 6.50 (d, 1H, $^3J_{NH',H2'}$ = 8.7 Hz, NH′), 6.09 (d, 1H, $^3J_{NH,H2}$ = 7.8 Hz, NH), 5.40 (s, 1H, C*H*Ph), 5.29 (s, 1H, C*H*Ph), 4.71 (d, 1H, ^2J = 11.0 Hz, CHa CH_2 Nap), 4.62 (d, 1H, $^3J_{H1',H2'}$ = 3.8 Hz, H-1′), 4.57-4.49 (m, 2H, CHb CH_2 Nap, CHa CH_2 Nap), 4.21 (d, 1H, ^2J = 11.8 Hz, CHb CH_2 Nap), 4.13 (m, 1H, H-2′); 4.05 (m, 1H, β^{Myr}-C*H*), 3.97 (dd, 1H, $^2J_{H6a',H6b'}$ = 9.9 Hz, $^3J_{H5',H6a'}$ = 4.8 Hz, H-6a′), 3.89 (dd, 1H, $^2J_{H6a,H6b}$ = 10.4 Hz, $^3J_{H5,H6a}$ = 5.1 Hz, H-6a), 3.75 (m, 1H, H-5′), 3.68-3.53 (m, 4H, H-2, H-3′, H-6b′, β^{Myr}-C*H*), 3.42 (m, 2H, H-6b), 3.30 (d, 1H, $^3J_{H1,H2}$ = 8.3 Hz, H-

1), 3.16 (dd, 1H, $^3J_{H3,H4}$ = $^3J_{H4,H5}$ = 9.2 Hz, H-4), 2.69 (dd, 1H, $^3J_{H2,H3}$ = $^3J_{H3,H4}$ = 9.4 Hz, H-3), 2.50-2.48 (m, 2H, 2xα^{Myr}-C*H*$_2$a), 2.43-2.41 (m, 2H, 2xα^{Myr}-C*H*$_2$b), 2.19 (ddd, 1H, $^3J_{H4,H5}$ = $^3J_{H5,H6a}$ = 9.7 Hz, $^3J_{H5,H6b}$ = 4.9, H-5), 1.58-1.19 (m, 40H, 20xCH$_2$ Myr), 0.83-0.79 (m, 6H, CH$_3$ Myr); ^{13}C NMR (75 MHz, CDCl$_3$) δ 173.82, 173.34 (2xCO, Myr), 137.27, 136.58, 135.48, 133.35, 133.29, 133.17, 133.13 (q, Aromatic), 129.38, 129.26, 128.71, 128.48, 128.39, 128.34, 127.98, 127.94, 126.96, 126.93, 126.71, 126.67, 126.64, 126.54, 126.48, 125.79 (CH, Arom.), 101.96 (C-1), 101.92, 101.82 (*C*HPh), 100.23 (C-1´), 81.89 (C-4´), 80.65 (C-4), 78.75 (β^{Myr}-*C*H), 77.19 (β^{Myr}-*C*H), 72.77 (CH$_2$ Nap), 72.39 (C-3), 71.50 (CH$_2$ Nap), 70.28 (C-3´), 68.65 (C-6´), 68.45 (C-6), 66.58 (C-5), 63.01 (C-5´), 56.42 (C-2), 53.95 (C-2´), 41.79, 41.21, 34.53, 33.54, 32.08, 29.98, 29.91, 29.81, 29.75, 29.52, 25.88, 25.30, 22.83 (CH$_2$ Myr), 14.25 (CH$_3$ Myr); HRMS (ESI) *m/z* calcd. for [M+H]$^+$ $C_{76}H_{100}N_2O_{13}$ 1249.7225, found 1249.7317.

4,6-*O*-Benzylidene-2-deoxy-3-*O*-[(*R*)-3-(naphthalen-2-ylmethoxy)dodecanoyl]-2-[(*R*)-3-(naphthalen-2-ylmethoxy)tetradecanoylamino]-β-D-glucopyranosyl-(1↔1)-4,6-*O*-benzylidene-2-deoxy-3-*O*-[(*R*)-3-(naphthalen-2-ylmethoxy)-dodecanoyl]-2-[(*R*)-3-(naphthalen-2-ylmethoxy)tetradecanoylamino]-α-D-glucopyranoside (18)

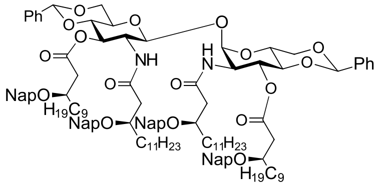

To a stirred solution of **17** (0.62 mmol, 0.78 g) in dry DCM (2 mL), a solution of **15** (2.2 equiv., 1.37 mmol, 0.49 g), *N*-ethyl-*N*′-(3-dimethylaminopropyl)carbodiimide hydrochloride (2.4 equiv., 1.50 mmol, 0.29 g) and 4-(dimethylamino)pyridine (0.1 equiv., 0.06 mmol, 8 mg) dissolved in in dry DCM (4 mL), was added and let stir for 2 h. at r.t. The mixture was diluted EtOAc (20 mL) and washed with satd. aq. NaHCO$_3$ (3x8 mL) and water (3x8 mL). The organic layer was dried over Na$_2$SO$_4$, filtrated over cotton, and concentrated. The residue was purified by column chromatography on silica gel (80 g), (toluene – acetone, 15:1 → 12:1), to give **18** (1.15 g, 96%) as colourless oil. R$_f$ = 0.5 (toluene - acetone = 10:1); $[\alpha]_D^{20}$= +107 (c 1.0, CHCl$_3$); ^{1}H NMR (300 MHz, CDCl$_3$) δ 7.87-7.65 (38H, Arom.), 6.51 (d, 1H, $^3J_{NH',H2'}$ = 9.4 Hz, NH´), 6.27 (d, 1H, $^3J_{NH,H2}$ = 8.7 Hz, NH), 5.37 (dd, 1H, $^3J_{H2,H3}$ = $^3J_{H3,H4}$ = 9.9 Hz, H-3´), 5.32 (s, 1H, C*H*Ph), 5.13 (s, 1H, C*H*Ph), 4.74 (d, 1H, ^2J= 10.7 Hz, CHa C*H*$_2$ Nap), 4.66-4.44 (m, 9H, H-1´, H-2´, H-3, CHb C*H*$_2$ Nap, CHa C*H*$_2$ Nap, 2xCH$_2$ Nap), 4.14 (d, 1H, ^2J = 12.1 Hz, CHa CH$_2$ Nap), 4.04 (m, 3H, H-5´, H-6´a, β $^{Myr\ and\ Lau}$-C*H*), 3.91-3.85 (m, 2H, H-2, H-6a), 3.82-3.75 (m, 2H, 2xβ$^{Myr\ and\ Lau}$-C*H*), 3.66-3.58 (m, 2H, H-4´, H-6´b), 3.53 (m, 1H, β$^{Myr\ and\ Lau}$-C*H*), 3.44 (dd, 1H, $^3J_{H5,H6b}$ = $^3J_{H6a,H6b}$ = 10.3 Hz, H-6b), 3.26 (dd, 1H, $^3J_{H3,H4}$ = $^3J_{H4,H5}$ = 9.4 Hz, H-4), 3.15 (d, 1H, $^3J_{H1,H2}$ = 8.3 Hz, H-1), 2.72-2.61 (m, 2H, α$^{Myr\ and\ Lau}$-C*H*$_2$), 2.51-2.18 (m, 7H, H-5, α$^{Myr\ and\ Lau}$), 1.48-1.19 (m, 72H, 36xCH$_2$ Myr And Lau), 0.83-0.79 (m, 12H, CH$_3$ Myr And Lau*);* ^{13}C NMR (75 MHz, CDCl$_3$) δ 172.43, 171.82, 171.57, 171.54 (4xCO, Myr and Lau), 137.01, 136.89, 136.34, 136.12, 135.89, 133.44, 133.38, 133.08, 133.04 (q, Arom.), 129.17, 129.08, 128.60, 128.29, 128.19, 128.10, 127.98, 127.85, 126.76, 126.93, 126.75, 126.69, 126.59, 126.51, 126.37, 126.20, 126.13, 126.00, 125.93, 125.76 (CH, Aromatic), 102.10 (C-1), 101.50, 101.32 (*C*HPh), 100.25 (C-1´), 79.26 (C-4´), 77.90 (C-4), 77.74 (β$^{Myr\ and\ Lau}$-*C*H), 77.36 (β$^{Myr\ and\ Lau}$-*C*H), 76.32 (β$^{Myr\ and\ Lau}$-*C*H), 75.64 (β$^{Myr\ and\ Lau}$-*C*H), 72.58 (*C*H$_2$ Nap), 71.30 (*C*H$_2$ Nap), 71.23 (C-3), 71.13 (*C*H$_2$ Nap), 70.87 (*C*H$_2$ Nap), 70.60 (C-3´), 68.65 (C-6´), 68.36 (C-6), 66.61 (C-5), 63.36 (C-5´), 53.88 (C-2), 51.55 (C-2´), 41.72, 41.31, 40.04, 39.94, 34.87, 34.72, 34.63, 33.72, 32.07, 30.02, 29.98, 29.89, 29.86, 29.79, 29.75, 29.53, 29.50, 25.89, 25.35, 25.27, 25.00, 22.84 (CH$_2$ Lau and Myr), 14.26 (CH$_3$ Lau and Myr); HRMS (ESI) *m/z* calcd. for [M+Na]$^+$ $C_{122}H_{160}N_2O_{17}$ Na 1948.1614, found 1948.1617.

2-Deoxy-3-*O*-[(*R*)-3-(naphthalen-2-ylmethoxy)dodecanoyl]-2-[(*R*)-3-(naphthalen-2-ylmethoxy)tetradecanoyl-amino]-β-D-glucopyranosyl-(1↔1)-2-deoxy-3-*O*-[(*R*)-3-(naphthalen-2-ylmethoxy)dodecanoyl]-2-[(*R*)-3-(naphthalen-2-ylmethoxy)tetradecanoylamino]-α-D-glucopyranoside (19)

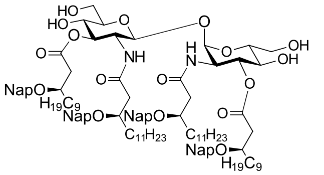

To a stirred solution of **18** (0.186 mmol, 0.36 g) in dry DCM (20 mL), a mixture of trifluoroacetic acid - H_2O (4:1, 2 mL) was added and the mixture was stirred for 2 h. at r.t., The reaction mixture was diluted with DCM (20 mL) and washed with satd. aq. $NaHCO_3$ (3 x 15 mL), the organic layer was dried over Na_2SO_4, filtered over cotton, and concentrated. The residue was purified by column chromatography on silica gel (30 g), (toluene – EtOAc, 12:1), to give **19** (0.28 g, 85%) as colourless sirup. R_f = 0.25 (toluene – EtOAc, 12:1); $[\alpha]_D^{20}$= +99 (c 1.0, $CHCl_3$); ^{1}H NMR (600 MHz, $CDCl_3$) δ 7.85-7.29 (28H, Arom.), 6.46 (d, 1H, $^3J_{NH',H2'}$ = 9.5 Hz, NH′), 6.21 (d, 1H, $^3J_{NH,H2}$ = 8.6 Hz, NH), 5.03 (dd, 1H, $^3J_{H2,H3}$ = $^3J_{H3,H4}$ = 9.8 Hz, H-3′), 4.71 (d, 1H, ^2J = 11 Hz, CHa CH_2 Nap), 4.67-4.60 (m, 5H, CHb CH_2 Nap, 2CH_2 Nap), 4.47-4.45 (m, 2H, H-1′, CHa CH_2 Nap), 4.36 (dd, 1H, $^3J_{H2,H3}$ = $^3J_{H3,H4}$ = 10.0 Hz, H-3), 4.29 (ddd, 1H, $^3J_{H2',H3'}$ = $^3J_{H2',NH'}$ = 10.1 Hz, $^3J_{H1',H2'}$ = 3.4 Hz, H-2′), 4.16 (m, 1H, ^2J = 12.1 Hz, CHb CH_2 Nap), 4.00 (m, 1H, $\beta^{\text{Myr and Lau}}$-C*H*), 3.94 (m, 1H, H-5′), 3.90-3.83 (m, 2H, 2x $\beta^{\text{Myr and Lau}}$-C*H*), 3.78-3.73 (m, 2H, H-2, H-6′a), 3.54-3.53 (m, 2H, H-6a, $\beta^{\text{Myr and Lau}}$-C*H*), 3.42 (dd, 1H, $^2J_{H6a',H6b'}$ = 12.1 Hz, $^3J_{H5',H6b'}$ = 8.8 Hz, H-6b′), 3.35-3.29 (m, 2H, H-4′, H-6b), 3.14.3.10 (m, 2H, H-1, H-4), 2.59 (dd, 1H, ^3J = 14.9 Hz, ^3J = 8.3 Hz, $\alpha^{\text{Myr and Lau}}$- C$H_2$a), 2.53 (dd, 1H, ^3J = 14.5 Hz, ^3J = 7.9 Hz, $\alpha^{\text{Myr and Lau}}$-C$H_2$a), 2.47-2.27 (m, 7H, H-5, 2x$\alpha^{\text{Myr and Lau}}$-C$H_2$b, 2x$\alpha^{\text{Myr and Lau}}$-C$H_2$), 1.61-1.23 (m, 72H, 36xCH_2 Myr and Lau), 0.89-0.87 (m, 12H, CH_3 Myr and Lau*);* ^{13}C NMR (151 MHz, $CDCl_3$) δ 172.94, 172.53, 172.49, 171.71 (4xCO, Myr and Lau), 136.80, 135.85, 135.70, 135.39, 133.40, 133.23, 133.17, 133.13 (q, Arom.), 128.63, 128.40, 128.31, 128.29, 128.11, 128.06, 127.92, 127.86, 127.81, 126.98, 126.92, 126.81, 126.78, 126.70, 126.63, 126.58, 126.43, 126.36, 126.31, 126.19, 126.15, 126.11, 126.02 (CH, Arom.), 101.67 (C-1), 99.33 (C-1′), 77.74 ($\beta^{\text{Myr and Lau}}$-*C*H), 76.19 (C-5), 76.16 ($\beta^{\text{Myr and Lau}}$-*C*H), 77.11 ($\beta^{\text{Myr and Lau}}$-*C*H), 75.91 ($\beta^{\text{Myr and Lau}}$-*C*H), 75.65 (C-3), 75.24 (C-3′), 72.63 (*C*H_2 Nap), 72.25 (C-5′), 71.30 (*C*H_2 Nap), 71.13 (*C*H_2 Nap), 70.75 (*C*H_2 Nap), 70.59 (C-4′), 68.86 (C-4), 63.27 (C-6′), 62.35 (C-6), 53.26 (C-2′), 50.64 (C-2), 41.83, 41.43, 39.95, 39.83, 34.70, 34.17, 34.06, 33.69, 32.08, 30.00, 29.93, 29.87, 29.77, 29.73, 29.55, 29.49, 29.90, 25.29, 25.25, 25.07, 22.84 (CH_2 Myr and Lau), 14.28 (CH_3 Myr and Lau); HRMS (ESI) *m/z* calcd. for $[M+H]^+$ $C_{108}H_{152}N_2O_{17}$ 1750.1091, found 1750.1160.

6-*O*-Allyloxycarbonyl-2-deoxy-3-*O*-[(*R*)-3-(naphthalen-2-ylmethoxy)dodecanoyl]-2-[(*R*)-3-(naphthalen-2-ylmethoxy)tetradecanoylamino]-β-D-glucopyranosyl-(1↔1)-6-*O*-allyloxycarbonyl-2-deoxy-3-*O*-[(*R*)-3-(naphthalen-2-ylmethoxy)dodecanoyl]-2-[(*R*)-3-(naphthalen-2-ylmethoxy)tetradecanoylamino]-α-D-glucopyranoside (20)

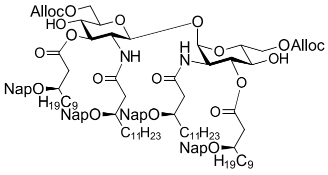

To a stirred solution of **19** (0.063 mmol, 0.11 g) in dry DCM (2 mL), 2,4,6-trimethylpyridine (5 equiv., 0.315 mmol, 41 µL) and allyloxycarbonyl chloride (15 equiv., 0.94 mmol, 113 mg) were added successively and the mixture was stirred for 4 h. at r.t. The mixture was diluted EtOAc (20 mL) and washed with satd. aq. $NaHCO_3$ (3x7 mL) and water (3x7 mL). The organic layer was dried over Na_2SO_4, filtered rated over cotton, and concentrated. The residue was purified by column chromatography on silica gel (20 g), (toluene – EtOAc, 4:1), to give **20** (105 mg, 85%) as colourless sirup. R_f = 0.4 (toluene – EtOAc, 4:1); $[\alpha]_D^{20}$= +81 (c 1.0, $CHCl_3$); ^{1}H NMR (600 MHz, $CDCl_3$) δ 7.84-7.20 (28H, Arom.), 6.44 (d, 1H, $^3J_{NH',H2'}$ = 9.5 Hz, NH′), 6.22 (d, 1H, $^3J_{NH,H2}$ = 8.6 Hz, NH), 6.01-5.98

(m, 2H, C*H*=CH$_2$), 5.42-5.25 (m, 4H, CH=C*H*$_2$), 5.09 (dd, 1H, $^3J_{H2,H3}$ = $^3J_{3,4}$ = 9.9 Hz, H-3′), 4.70-4.57 (m, 1H, H-1′, CH=CH$_2$C*H*$_2$, 3xCH$_2$ Nap), 4.42 (d, 1H, ^2J = 12.1 Hz, CHa CH$_2$ Nap), 4.39-4.35 (m, 2H, H-2′, H-6a′), 4.30 (m, 3H, H-3, H-6a, H-6b′), 4.06 (m, 1H, ^2J = 12.1 Hz, CHb CH$_2$ Nap), 4.03-3.97 (m, 2H, H-5′, β$^{\text{Myr and Lau}}$-C*H*), 3.90-3.91 (m, 1H, H-6b), 3.90-3.85 (m, 2H, 2xβ$^{\text{Myr and Lau}}$-C*H*), 3.75 (m, 1H, H-2), 3.66 (m, 1H, H-4′), 3.45 (m, 1H, β$^{\text{Myr and Lau}}$-C*H*), 3.24 (dd, 1H, $^3J_{H3,H4}$ = $^3J_{H4,H5}$ 9.3 Hz, H-4), 3.16 (d, 1H, $^3J_{H1,H2}$ = 8.3 Hz, H-1), 2.89 (br.s., 1H, OH′), 2.72 (br.s., 1H, OH), 2.59 (dd, 1H, ^3J = 15.2 Hz, ^3J = 7.9 Hz, α$^{\text{Myr and Lau}}$-C*H*$_2$a), 2.53 (dd, 1H, ^3J = 14.5 Hz, ^3J = 7.8 Hz, α$^{\text{Myr and Lau}}$-C*H*$_2$a), 2.47-2.20 (m, 7H, H-5, 2xα$^{\text{Myr and Lau}}$-C*H*$_2$b, 2xα$^{\text{Myr and Lau}}$-C*H*$_2$), 1.50-1.39 (m, 72H, 36xCH$_2$ Myr and Lau), 0.89-0.87 (m, 12H, CH$_3$ Myr and Lau); ^{13}C NMR (151 MHz, CDCl$_3$) δ 172.72, 172.48, 172.44, 171.64 (4xCO, Laur. and Myr), 155.55, 155.23 (q, CH$_2$=CHCH$_2$*C*O), 136.89, 136.00, 135.79, 135.54, 133.44, 133.23, 133.14, (q, Arom.), 131.74, 131.67 (*C*H=CH$_2$), 128.75, 128.38, 128.25, 128.10, 128.06, 127.98, 127.86, 127.79, 126.87, 126.94, 126.85, 126.72, 126.70, 126.66, 126.52, 126.37, 126.32, 126.17, 126.13, 126.09, 125.88 (*C*H, Arom.), 119.20, 119.18 (CH=*C*H$_2$), 101.14 (C-1), 99.69 (C-1′), 77.67, 76.36, 76.05, 75.92 (β$^{\text{Myr and Lau}}$-*C*H), 75.22 (C-3), 74.66 (C-3′), 73.11 (C-5), 72.61, 71.57, 71.32 (CH$_2$ Nap), 70.88 (C-5′), 70.86 (CH$_2$ Nap), 68.83, 68.78 (O*C*H$_2$CH=CH$_2$), 68.66 (C-4′), 68.24 (C-4), 66.27 (C-6′), 65.90 (C-6), 53.03 (C-2′), 50.74 (C-2), 41.74, 41.28, 40.07, 39.87, 34.77, 34.45, 34.19, 33.74, 32.12, 32.06, 30.05, 29.98, 29.92, 29.86, 29.80, 29.74, 29.58, 29.50, 25.91, 25.32, 24.95, 22.86 (CH$_2$ Laur. and Myr), 14.27 (CH$_3$ Laur. and Myr); HRMS (ESI) *m/z* calcd. for [M+H]$^+$ $C_{116}H_{161}N_2O_{21}$ 1918.1591, found 1918.1599.

6-*O*-Allyloxycarbonyl-2-deoxy-3-*O*-[(*R*)-3-(naphthalen-2-ylmethoxy)dodecanoyl]-4-*O*-[(2-cyanoethoxy)]-2-[(9-fluorenylmethoxycarbonylamino)ethoxy-phosphoryl]-2-[(*R*)-3-(naphthalen-2-ylmethoxy)tetradecanoylamino]-β-D-glucopyranosyl-(1↔1)-6-*O*-allyloxycarbonyl-2-deoxy-3-*O*-[(*R*)-3-(naphthalen-2-ylmethoxy)dodecanoyl]-4-*O*-[(2-cyanoethoxy)]-2-[(9-fluorenylmethoxycarbonylamino)ethoxy-phosphoryl]-2-[(*R*)-3-(naphthalen-2-ylmethoxy)tetradecanoylamino]-α-D-glucopyranoside (21)

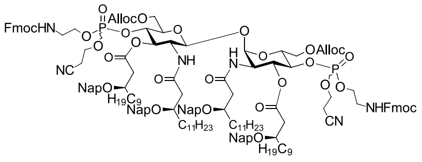

To a stirred solution of **20** (0.062 mmol, 0.12 g) in dry DCM (6 mL), a solution of **2** (5 equiv., 0.31 mmol, 122 mg) in dry DCM (1 mL) and 1*H*-Tetrazole (0.45 M in acetonitrile, 5 equiv., 0.68 mL) were added successively at r.t. and the reaction mixture was stirred for 20 min in the atmosphere of Ar. The reaction mixture was cooled to -78 °C and *m*-chloroperbenzoic acid (6.5 equiv., 0.4 mmol, 0.07 g) in dry DCM (2 mL) was added and the stirring was continued for 2 h at -78 °C. A solution of Et$_3$N (13 equiv., 0.8 mmol, 0.1 mL) in dry MeOH (1 mL) was added at -78 °C and the reaction mixture was stirred for xx min while warming up to r.t. The mixture was diluted with EtOAc (40 mL) and washed with 10% aq. Na$_2$S$_2$O$_3$ (3x10 mL) and satd. aq. NaHCO$_3$ (3x10 mL), the organic phase was dried over Na$_2$SO$_4$, filtered and concentrated. The residue was purified by column chromatography on silica gel (120 g), (hexane – acetone, 2:1.5), appropriate fractions were collected and purified by size exclusion chromatography of BioRad SX1 column (800x 5mm), (toluene - CH$_2$Cl$_2$, 2:1), to give **21** (150 mg, 89%) as colorless sirup. R$_f$ = 0.4 (hexane – acetone, 2:1); $[\alpha]_D^{20}$= +66 (c 1.0, CHCl$_3$); ^{1}H NMR (600 MHz, acetone-d$_6$) δ 7.85-7.29 (m, 44H, Arom.), 6.93 (m, 1H, NH), 6.65-6.54 (m, 3H, NH′, 2xNH Fmoc), 6.05-5.96 (m, 2H, 2xOCH$_2$C*H*=CH$_2$), 5.44-5.25 (m, 5H, H-3′, 2xOCH$_2$CH=C*H*$_2$), 5.05 (m, 1H, H-3), 4.92 (m, 1H, H-1′), 4.76-4.61 (m, 13H, H-4′, 2xOC*H*$_2$CH=CH$_2$, 4xCH$_2$ Nap), 4.51-3.89 (m, 26H, H-1, H-2, H-2′, H-4, H-5′, H-6, H-6′, 2xC*H* Fmoc, 2xC*H*$_2$ Fmoc, β^{Lau}-C*H*, 2xβ^{Myr}-C*H*, 2xOC*H*$_2$CH$_2$NHC(O)Fmoc, 2xOC*H*$_2$CH$_2$CN), 3.64 (m, 1H, β^{Lau}-C*H*), 3.45-3.279 (m, 5H, H-5, 2xOCH$_2$C*H*$_2$NHC(O)Fmoc), 2.86-2.76 (m, 5H, CHa α^{Lau}-C*H*$_2$ 2xOCH$_2$C*H*$_2$CN), 2.73-2.25 (m, 7H, CHb α^{Lau}-C*H*$_2$, 2xα^{Myr}-C*H*$_2$, α^{Lau}-C*H*), 1.58-1.13 (m, 72H, 36xCH$_2$- Lau and Myr), 0.88-0.83 (m, 12H, 4xCH$_3$- Lau and Myr); ^{13}C NMR (151 MHz, acetone-d$_6$) δ 173.30, 173.27,173.25, 172.48, 172.45, 172.38, 172.33, 171.78, 171.75 (4x*C*O Lau and Myr), 157.45, 157.43, 157.40, 154.36 (*C*O Fmoc), 155.76, 155.72, 155.70 (CO Alloc), 145.34, 145.32, 145.29, 145.25, 142.30, 138.17, 137.98, 137.93, 137.92, 137.88, 137.60, 134.48, 134.46, 134.44, 134.11, 134.06, (Cq, Arom.), 133.44, 1333.38, 133.30, 133.29, 133.26, 133.23 (OCH$_2$*C*H=CH$_2$), 129.11, 129.01, 128.92, 128.91, 128.86, 128.84, 128.81, 128.76, 128.75, 128.69, 128.68, 128.14, 127.51, 127.47, 127.42, 127.40, 127.36,

127.33, 127.31, 127.28, 127.18, 127.03, 127.01, 127.00, 126.94, 126.88, 126.74, 126.71, 126.38, 126.31, 120.97 (*C*H, Arom.), 119.20, 119.12, 119.09 and 118.99 ($OCH_2CH{=}CH_2$), 118.27, 118.25 and 118.18 (*C*N), 101.81 (C-1), 100.37 (C-1´), 77.84, 77.17, 76.31, 76.24, 76.18, 76.10 (4xβ$^{\text{Lau and Myr}}$-*C*H), 74.36, 74.33, 74.27, 74.22, 74.13 and 74.10 (C-4, C-4´), 73.31, 73.24, 73.18 and 73.13 (*C*-3, C-5), 72.92 (CH_2 Nap), 72.94 (C-3´), 71.95, 71.83, 71.65, 71.58 (CH_2 Nap), 70.30, 70.25 and 70.21 (C-5´), 69.49, 69.44, 69.38 and 69.29 ($OCH_2CH{=}CH_2$), 68.34, 68.29, 68.26 and 68.20 (2xCH_2 Fmoc), 67.42, 67.37 and 67.31 (2x$OCH_2CH_2NH_2$), 64.11, 64.07, 64.03, 63.95 and 63.91 (C-6, C-6´), 67.08, 67.02 ($OCH_2CH_2NHC(O)$Fmoc), 62.40, 62.28 (OCH_2CH_2CN), 54.88 (C-2), 51.94 (C-2´), 48.26, 48.24 (CH Fmoc), 42.28 (α$^{\text{Lau and Myr}}$-CH_2), 42.16, 42.10 ($OCH_2CH_2NHC(O)$Fmoc), 40.31, 40.26, 40.20, 40.15, 35.59, 35.36, 35.31, 35.29, 34.73, 32.88, 26.57, 26.30, 26.25, 26.18, 25.86, 23.57 (36xCH_2- Lau and Myr), 20.10, 19.99 and 19.94 (OCH_2CH_2CN), 14.59 (4xCH_3- Lau and Myr); ^{31}P NMR (243 MHz, acetone-d_6) δ -2.17, -2.19, -2.20, -2.25; HRMS (ESI) *m/z* calcd. for $[M+2H]^{+2}$ $C_{156}H_{200}N_6O_{31}P_2$ 1357.6866, found 1357.6859.

6-*O*-Allyloxycarbonyl-2-deoxy-3-*O*-[(*R*)-3-(hydroxy)dodecanoyl]-4-*O*-[(2-cyanoethoxy)]-2-[(9-fluorenylmethoxy-carbonylamino)ethoxy-phosphoryl]-2-[(*R*)-3-(hydroxy)tetradecanoylamino]-β-D-glucopyranosyl-(1↔1)-6-*O*-allyloxycarbonyl-2-deoxy-3-*O*-[(*R*)-3-(hydroxy)dodecanoyl]-4-*O*-[(2-cyanoethoxy)]-2-[(9-fluorenylmethoxy-carbonylamino)ethoxy-phosphoryl]-2-[(*R*)-3-(hydroxy)tetradecanoylamino]-α-D-glucopyranoside (22)

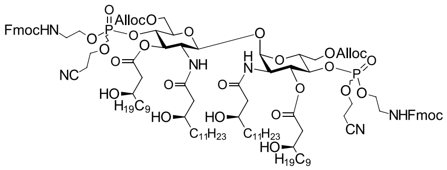

To a stirred solution of **21** (0.055 mmol, 150 mg) in a mixture of $CHCl_3$ - H_2O (10:1, 3.3 mL), 2,3-dichloro-5,6-dicyano-p-benzoquinone (20 equiv., 0.33 mmol, 75 mg) was added at r.t. and reaction mixture was stirred for 3 h. DCM (20 mL) was added and the reaction mixture was extracted with satd. aq. $NaHCO_3$ (3x5 mL) and water (3x5 mL), the organic phase was dried over Na_2SO_4, filtrated over cotton and the solvent concentrated. The residue was purified by column chromatography on silica gel (50 g), (DCM → DCM – MeOH, 10:3), to give **22** (104 mg, 88 %) as white foam. R_f = 0.3 (DCM – MeOH, 10:0.5); $[\alpha]_D^{20}$= +17 (c 1.0, CH_3OH); ^{1}H NMR (600 MHz, CD_3OD) δ 7.79-7.30 (m, 16H, Fmoc), 5.99-5.86 (m, 2H, 2xCH $OCH_2CH{=}CH_2$), 5.37-5.19 (m, 6H, H-3, H-3´, 2xCH_2 $OCH_2CH{=}CH_2$), 5.05 (m, 1H, H-1´), 4.77 (m, 1H, H-1), 4.68-4.45 (m, 8H, H-4, H-4´, H-6a, H-6´a, 2xCH_2 $OCH_2CH{=}CH_2$), 4.38-4.11 (m, 19H, H-2, H-2´, H-5´, H-6b, H-6´b, 2x*C*H Fmoc, 2xCH_2 Fmoc, 2xOCH_2CH_2CN, 2xOCH_2CH_2NHFmoc), 4.02-3.92 (m, 4H, 4xβ$^{\text{Lau and Myr}}$-*CH*), 3.86 (m, 1H, H-5), 3.44-3.39 (m, 4H, 2xOCH_2CH_2NHFmoc), 2.83-2.78 (m, 4H, OCH_2CH_2CN), 2.58-2.2 (m, 8H, 4xα$^{\text{Lau and Myr}}$-CH_2), 1.50-1.39 (m, 72H, 36xCH_2 Lau and Myr), 0.89-0.87 (m, 12H, CH_3 Lau and Myr); ^{13}C NMR (151 MHz, CD_3OD) δ 175.14, 175.11, 174.84, 174.82, 173.52, 173.13, 173.11 (4xCO, Laur. and Myr), 158.96, 158.92, 158.89 (q, CO Fmoc), 156.34, m 156.29 (q, $CH_2{=}CHCH_2CO$), 145.52, 142.82 (q, aromatic Fmoc), 133.39, 133.33, 133.29 ($OCH_2CH{=}CH_2$), 128.98, 128.36, 126.46, 126.43, 126.40, 121.13 (CH, aromatic), 119.34, 119.32, 119.29 ($OCH_2CH{=}CH_2$), 118.56 (*C*N) 102.14 (C-1), 100.52 (C-1´), 75.44, 75.34, 75.24, 75.18 (C-4, C-4´), 74.31, 74.32 (C-3), 74.03, 73.97 (C-5), 72.39, 72.32 (C-3´), 70.47, 70.43 (C-5´), 69.99, 69.96 ($OCH_2CH{=}CH_2$), 69.90, 69.87, 69.83, 69.19, 69.17, 69.09, 69.05, 69.03, 69.00 (β$^{\text{Lau and Myr}}$-*C*H), 69.03, 69.00 (OCH_2CH_2NHFmoc), 68.15, 68.10 (CH_2 Fmoc), 66.85, 66.77, 66.64, 66.60, 66.56, 66.54, (C-6, C-6´), 64.82, 64.78, 64.76, 64.71, 64.65, (OCH_2CH_2CN), 55.07 (C-2´), 52.94 (C-2), 48.62 (CH Fmoc), 45.10, 44.54, 43.89, 43.84, 43.74, 43.68 (CH_2 Laur. and Myr), 42.17, 42.12 (OCH_2CH_2NHFmoc), 38.98, 38.94, 38.45, 38.37, 33.26, 33.26, 31.13, 31.11, 31.09, 31.06, 31.04, 31.03, 31.02, 30.99, 30.96, 30.71, 30.70, 27.04, 26.97, 26.93, 23.91 (CH_2 Laur. and Myr), 20.18, 20.13 (OCH_2CH_2CN), 14.62 (CH_3 Laur. and Myr); ^{31}P NMR (243 MHz, CD_3OD) δ -3.17, -3.20, -3.27; HRMS (ESI) *m/z* calcd. for $[M+H]^+$ $C_{112}H_{167}N_6O_{31}P_2$ 2154.1151, found 2154.1187.

2-Deoxy-3-*O*-[(*R*)-3-(hydroxy)dodecanoyl]-4-*O*-[(2-cyanoethoxy)]-2-[(9-fluorenylmethoxycarbonyl-amino)ethoxy-phosphoryl]-2-[(*R*)-3-(hydroxy)tetradecanoylamino]-β-D-glucopyranosyl-(1↔1)-2-deoxy-3-*O*-[(*R*)-3-(hydroxy)dodecanoyl]-4-*O*-[(2-cyanoethoxy)]-2-[(9-fluorenylmethoxycarbonylamino)ethoxy-phosphoryl]-2-[(*R*)-3-(hydroxy)tetradecanoylamino]-α-D-glucopyranoside (23)

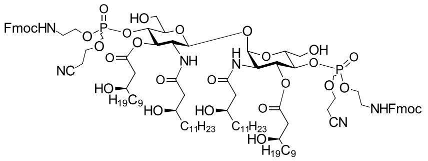

To a stirred solution of **22** (0.016 mmol, 34 mg) in 0.5 mL of dry CH_2Cl_2, bis(triphenylphisohine)Pd(II)dichloride 15.2% (0.15 equiv., 0.002 mmol, 2 mg) and tributyltin hydride (1.5 equiv., 0.023 mmol, 6 µL) were added at r.t. and reaction mixture was stirred for 1 h. The stirrer was removed, silica (300 mg) was added, and the solvent concentrated. The residue was purified by column chromatography on silica gel (30 g), (Hexane → DCM → DCM – MeOH, 10:1) to give **23** (27 mg, 87 %) as white foam. R_f = 0.3 (DCM – MeOH, 10:0.8); $[\alpha]_D^{20}$= +22 (c 1.0, CH_3OH); ^{1}H NMR (600 MHz, CD_3OD - $CDCl_3$, 3:1;) δ 8.06 (d, 0.5 H, $^3J_{NH,H2}$ = 10.4 Hz, NH), 7.77-7.63 (m, 8H, Fmoc), 7.47 (d, 1H, NH´), 7.38-7.26 (m, 8H, Fmoc), 5.32-5.23 (m, 2H, H-3, H-3´), 5.07 (m, 1H, H-1´), 4.72 (m, 1H, H-1), 4.53-4.47 (m, 2H, H-4, H-4´), 4.36-4.33 (m, 4H, 2xCH_2 Fmoc), 4.27-4.06 (m, 14H, H-2, H-2´, H-5´, 2xCH Fmoc, 2xO$CH_2$$CH_2$CN, 2xO$CH_2$$CH_2$NHFmoc, $\beta^{Lau\ and\ Myr}$-C*H*), 4.00-3.91 (m, 3H, 3x$\beta^{Lau\ and\ Myr}$-C*H*), 3.84-3.81 (m, 2H, H-6a, H6´a), 3.75-3.73 (m, 2H, H-6b, H6´b), 3.56 (m, 1H, H-5), 3.42-3.39 (m, 4H, 2xO$CH_2$$CH_2$NHFmoc), 2.80-2.77 (m, 4H, 2xO$CH_2$$CH_2$CN), 2.56-2.20 (m, 8H, 4x$\alpha^{Lau\ And\ Myr}$ CH_2), 1.42-1.23 (m, 72H, 36xCH_2 Laur. and Myr), 0.88-0.85 (m, 12H, 4xCH_3 Laur. and Myr); ^{13}C NMR (151 MHz, CD_3OD - $CDCl_3$, 3:1;) δ 174.84, 174.80, 174.74, 174.71, 173.63, 173.59, 173.08, 173.00 (4x CO, Laur. and Myr), 158.73, 158.69 (CO Fmoc), 145.21, 145.17, 142.51 (q, aromatic Fmoc), 128.78, 128.14, 126.21, 120.96 (CH, Aromatic), 118.32 (CN) 102.42, 102.37, 102.34 (C-1), 100.10 (C-1´), 76.32, 76.28, 76.21, 76.17 (C-5), 75.47, 75.43, 75.32, 75.27, 75.05, 75.02, 74.85, 74.80 (C-4, C-4´), 74.58 (C-3), 72.66, 72.66 (C-5´), 72.28, 72.20 (C-3´), 69.93, 69.81, 69.09, 69.01, 68.88, 68.83, 68.79 ($\beta^{Laur.\ and\ Myr}$-*C*H), 69.72, 69.67 (O*C*H$_2$CH$_2$NHFmoc), 67.99 (CH_2 Fmoc), 64.36, 64.32, 64.28 (O*C*H$_2$CH$_2$CN), 61.44, 61.16, 61.13 (C-6, C-6´), 54.94 (C-2), 53.08 (C-2´), 48.35 (CH Fmoc), 44.89, 44.29, 43.77, 43.66, 43.58, 43.48 (CH_2 Laur. and Myr), 42.02, 41.89,41.92 (OCH$_2$*C*H$_2$NHFmoc), 38.92, 38.88, 38.21, 38.15, 33.05, 30.92, 30.88, 30.82, 30.90, 30.76, 30.74, 30.50, 30.47, 29.08, 26.85, 26.82, 26.76, 26.74, 26.66, 26.63, 26.59, 23.72 (CH_2 Laur. and Myr), 20.08, 20.03 (OCH$_2$CH$_2$*C*N), 14.59 (CH_3 Laur. and Myr); ^{31}P NMR (243 MHz, CD_3OD - $CDCl_3$, 3:1;) δ -3.74, -3.78, -4.01, -4.04; HRMS (ESI) *m/z* calcd. for $[M + H]^+$ $C_{104}H_{159}N_6O_{27}P_2$ 1986.0728, found 1986.0719.

2-Deoxy-3-*O*-[(*R*)-3-(hydroxyl)dodecanoyl]-4-*O*-[(2-aminoethoxy)phosphoryl]-2-[(*R*)-3-(hydroxyl)tetradecanoyl-amino]-β-D-glucopyranosyl-(1↔1)-2-deoxy-3-*O*-[(*R*)-3-(hydroxyl)dodecanoyl]-4-*O*-[(2-cyanoethoxy)-phosphoryl]-2-[(*R*)-3-(hydroxyl)tetradecanoylamino]-α-D-glucopyranoside (24)

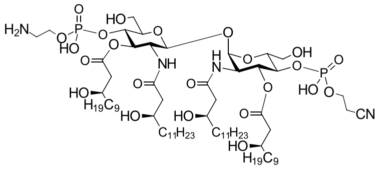

To a stirred solution of **23** (0.005 mmol, 10 mg) in dry DMSO (2 mL), Et_3N (0.5 mL) was added at r.t. and reaction mixture was stirred for 16 h. The stirrer was removed and the solvent concentrated, the residue was purified by size exclusion chromatography on BioRad SX1 (800x5mm), (toluene - CH_2Cl_2, 2:1), to give **24** (4 mg, 60 %) as white powder. ^{1}H NMR (600 MHz, $CDCl_3$ – CD_3OD, 1:3) δ 5.28-5.19 (m, 2H, H-3, H-3´), 5.06 (d, 1H, $^3J_{H1´,H2´}$ = 3.8 Hz, H-1´), 4.73 (m, H-1), 4.38-4.30 (m, 2H, H-4, H-4´), 4.20 (dd, 1H, $^3J_{H2´,H3´}$ = 11.0 Hz, $^3J_{H1´,H2´}$ = 3.6 Hz, H-2´), 4.15-3.93 (m, 10H, H-2, H-5´, 4x$\beta^{Lau\ and\ Myr}$-C*H*, OC*H*$_2$CH$_2$NH$_2$, OC*H*$_2$CH$_2$CN), 3.83-3.79 (m, 4H, H-6, H´-6), 3.48 (m, 1H, H-5), 3.19-3.16 (m, 2H, OCH$_2$C*H*$_2$NH$_2$), 2.83-2.80 (m, 2H, OCH$_2$C*H*$_2$CN), 2.55-2.21 (m, 8H, 2x$\alpha^{Lau\ And\ Myr}$-C*H*$_2$), 1.44-1.27 (m, 72H, 36xCH Lau and Myr), 0.89-0.86 (m, 12H, 4xCH_3 Lau and Myr); ^{13}C NMR (151 MHz, $CDCl_3$ – CD_3OD, 1:3) δ

173.43, 173.29, 172.70, 171.94 (4xCO, Lau and Myr), 100.95 (C-1), 98.75 (C-1´), 72.26 (C-5), 74.65 (C-3), 72.00 (C-5´), 71.46 (C-3´), 72.66 (C-4, C-4´), 68.69, 68.65, 67.92, 67.77 (4x$\beta^{\text{Lau and Myr}}$-*C*H), 62.63 (O*C*$H_2CH_2NH_2$, O*C*$H_2CH_2CN$), 60.37 (H-6, H-6´), 53.4 (C-2), 51.72 (C-2´), 43.69, 43.40, 43.10, 42.25, 42.05, (CH_2 Laur. and Myr), 39.76 (OCH_2*C*H_2NH_2), 37.42, 32.00, 29.41, 25.03, 24.78, 22.29, (CH_2 Laur. and Myr), 18.61 (OCH_2*C*H_2CN), 13.51 (CH_3 Laur. and Myr); ^{31}P NMR (243 MHz, $CDCl_3$ – CD_3OD, 1:3) δ -0.27, -0.36. All ^{13}C shifts except for those arising from carbonyl carbons were gained from HSQC. Carbonyl ^{13}C shifts were obtained from HMBC; ^{31}P NMR (243 MHz, $CDCl_3$ – CD_3OD, 1:3) δ -1.11, -1.42.

2-Deoxy-3-*O*-[(*R*)-3-(hydroxyl)dodecanoyl]-4-*O*-[(2-aminoethoxy)phosphoryl]-2-[(*R*)-3-(hydroxyl)tetradecanoyl-amino]-β-D-glucopyranosyl-(1↔1)-2-deoxy-3-hydroxy-4-*O*-[(2-aminoethoxy)phosphoryl]-2-[(*R*)-3-(hydroxyl)-tetradecanoylamino]-α-D-glucopyranoside (25)

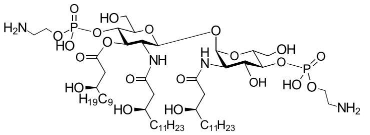

To a stirred solution of **23** (0.005 mmol, 10 mg) in a dry mixture of DCM – ACN (1:1, 2 mL), tris(2-aminoethyl)amine (20 equiv., 0.1 mmol, 13 µL) was added at r.t. and reaction mixture was stirred for 1 h. The mixture was diluted $CHCl_3$ (10 mL) and washed with PBS buffer pH 5.5 (2x5 mL). The organic layer was dried over Na_2SO_4, filtrated over cotton, and concentrated. The residue was purified by size exclusion chromatography on Sephadex LH-20 support (DCM – MeOH, 1:2) to give a mixture of βα-DLAM44 and **25** (2:1, 4 mg, 60%) as a white powder. ^{1}H NMR (600 MHz, $CDCl_3$ – CD_3OD, 1:3) δ 5.22 (dd, 1H, $^{3}J_{H2´,H3´}$ = $^{3}J_{H3´,H4´}$ = 10.0 Hz, H-3´), 5.16 (dd, 1H, $^{3}J_{H2,H3}$ = $^{3}J_{H3,H4}$ = 9.8 Hz, H-3), 5.10 (m, 1H, H-1´cleav, H-3cleav), 5.04 (d, 1H, $^{3}J_{H1´,H2´}$ = 3.4 Hz, H-1´), 4.70 (d, 1H, $^{3}J_{H1,H2}$ = 8.2 Hz, H-1), 4.63 (d, 0.5H, $^{3}J_{H1,H2}$ = 8.2 Hz, H-1cleav), 4.27-4.18 (m, 3H, H-2´, H-4, H-4´), 4.09-3.93 (m, 11H, H-2, H-2cleav, H-5´, H-4´cleav, 4x$\beta^{\text{Lau and Myr}}$-*CH,* 2xO*C*$H_2CH_2NH_2$), 3.89-3.75 (m, 5H, H-2´cleav, H-5´cleav, H-6, H´-6), 3.46 (m, 1H, H-5), 3.12-3.12 (m, 4H, $2xOCH_2$*C*H_2NH_2), 2.51-2.23 (m, 8H, 2x$\alpha^{\text{Lau And Myr}}$-*C*$H_2$), 1.43-1.27 (m, 72H, 36xCH Lau and Myr), 0.89-0.86 (m, 12H, 4xCH_3 Lau and Myr); ^{13}C NMR (151 MHz, $CDCl_3$ – CD_3OD, 1:3) δ 173.43, 173.29, 172.70, 171.94 (4xCO, Lau and Myr), 101.38 (C-1cleav), 101.06 (C-1), 98.80 (C-1´, C-1´cleav), 76.01 (C-5), 74.51 (C-3cleav), 74.33 (C-3), 74.09 (C-5´cleav), 72.46 (C-3´cleav), 72.36 (C-5´), 72.01 (C-3´), 71.59 (C-4, C-4´), 68.61, 67.47, 67.61 (4x$\beta^{\text{Lau and Myr}}$-*C*H), 63.55, 61.68 (2xO*C*$H_2CH_2NH_2$), 60.53, 60.53 (H-6, H-6´), 54.52 (C-2´ cleav), 53.91 (C-2), 52.13 (C-2´), 43.56, 42.20 (CH_2 Laur. and Myr), 40.27 ($2xOCH_2$*C*H_2NH_2), 36.98, 31.69, 29.57, 25.43, 22.38, (CH_2 Laur. and Myr), 13.51 (CH_3 Laur. and Myr); All ^{13}C shifts except for those arising from carbonyl carbons were gained from HSQC. Carbonyl ^{13}C shifts were obtained from HMBC; ^{31}P NMR (243 MHz, $CDCl_3$ – CD_3OD, 1:3) δ -0.27, -0.36.

2-Deoxy-3-*O*-[(*R*)-3-(naphthalen-2-ylmethoxy)dodecanoyl]-4-*O*-[(2-aminoethoxy)phosphoryl]-2-[(*R*)-3-(naphthalen-2-ylmethoxy)tetradecanoylamino]-β-D-glucopyranosyl-(1↔1)-2-deoxy-3-*O*-[(*R*)-3-(naphthalen-2-ylmethoxy)dodecanoyl]-4-*O*-[(2-aminoethoxy)phosphoryl]-2-[(*R*)-3-(naphthalen-2-ylmethoxy)tetradecanoyl-amino]-α-D-glucopyranoside (27)

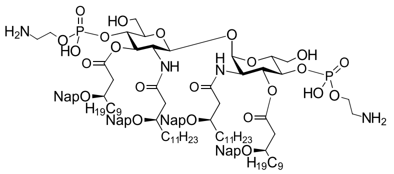

To a stirred solution of **21** (0.041 mmol, 112 mg) in dry CH_2Cl_2, bis(triphenylphisohine)Pd(II)dichloride 15.2% (0.1 equiv., 0.006 mmol, 4 mg) and tributyltin hydride (3 equiv., 0.123 mmol, 33 µL) were added at r.t. and reaction mixture was stirred for 1 h. The stirrer was removed, silica gel (1 g) was added, and the solvent concentrated. The residue was purified by column chromatography on silica gel (30 g), (DCM → DCM – MeOH, 10:1). Products containing fractions were pooled together and concentrated. The residue was dissolved in a dry mixture of DCM – ACN (1:1, 2 mL), Et_3N (0.4 mL) was added at r.t. and reaction mixture was stirred

for 24 h. The stirrer was removed, silica gel (0.5 g) was added, and the solvent concentrated. The residue was purified by column chromatography on silica gel (5 g), (DCM → DCM – MeOH, 10:4, with 0.1% of Et_3N), to give **27** (59 mg, 72% over two steps) as white foam. R_f = 0.2 (DCM - MeOH, 10:4 with 0.1% Et_3N); $[\alpha]_D^{20}$= +139 (c 1.0, $CHCl_3$); ^1H NMR (600 MHz, CD_3OD - $CDCl_3$, 2:1) δ 7.78-7.37 (m, 30H, Nap), 7.25 (d, 1H, $^3J_{H2,NH}$ = 8.5 Hz, NH), 6.284 (m, 1H, NH′), 5.32 (dd, 1H, $^3J_{H2′,H3′}$ = $^3J_{H3′,H4′}$ = 9.8 Hz, H-3′), 4.90 (m, 1H, H-3), 4.83 (m, 1H, H-1′), 4.65-4.51 (m, 6H, 3xCH_2 Nap), 4.37-4.29 (m, 4H, H-2′, H-4′, CH_2 Nap), 4.19-4.15 (m, 2H, H-4, H-5′), 3.97-3.73 (m, 12H, H-1, H-2, H-6a, H-6′, 3xβ$^{\text{Lau and Myr}}$- C*H*, 2xOC*H*$_2$CH$_2$NH$_2$), 3.60 (m, 1H, H-6b), 3.49 (m, 1H, β$^{\text{Lau and Myr}}$- C*H*), 3.05 (m, 1H, CH_2 TEA), 2.84 (m, 1H, H-5), 2.71-2.12 (m, 12H, 2xOCH$_2$C*H*$_2$NH$_2$, α$^{\text{Lau and Myr}}$-C*H*$_2$), 1.37-1.09 (m, 72H, 36xCH_2 Laur. and Myr), 0.90 (t, 2H, CH_3 Tea) 0.86-0.84 (m, 12H, 4xCH_3 Laur. and Myr); ^{13}C NMR (151 MHz, CD_3OD - $CDCl_3$, 2:1) δ 173.94, 173.36, 172.95, 172.69 (4xCO, Laur. and Myr), 137.48, 137.09, 136.98, 136.94, 134.33, 134.08 (q, aromatic Nap), 128.98, 128.95, 128.88, 128.79, 128.75, 128.64, 128.58, 128.53, 127.75, 127.73, 127.73, 127.64, 127.57, 127.40, 127.28, 127.20, 127.15, 127.05, 126.93, 126.85, 126.82, 126.79 (CH, Aromatic), 101.84 (C-1), 100.39 (C-1′), 77.96, 77.04, 76.64 (3xβ$^{\text{Laur. and Myr}}$-*C*H), 76.55 (C-5), 76.45 (β$^{\text{Laur. and Myr}}$-*C*H), 74.35 (C-3), 73.52 (C-3′), 73.34 (C-5′), 73.16 (*C*H$_2$ Nap), 72.90 (d, $^2J_{P,C4′}$ = 5.4 Hz, C-4′), 72.58 (CH_2 Nap), 72.33 (d, $^2J_{P,C4}$ = 5.4 Hz, C-4), 72.58, 71.12 (2xCH_2 Nap), 62.81 (d, $^2J_{P,C}$ = 6.7 Hz, O*C*H$_2$CH$_2$NH$_2$), 62.72 (d, $^2J_{P,C}$ = 6.8 Hz, O*C*H$_2$CH$_2$NH$_2$), 61.52, 61.45 (C-6, C-6′), 55.11 (C-2), 52.60 (C-2′), 47.47 (CH_2 Tea), 42.33, 41.95 (CH_2 Laur. and Myr), 41.32 (d, $^3J_{P,C}$ = 4.7 Hz, OCH$_2$*C*H$_2$NH$_2$), 40.29, 40.24, 35.29, 35.17, 34.65, 32.90, 30.81, 30.72, 30.67, 30.63, 30.58, 30.34, 28.87, 27.95, 26.45, 26.27, 26.06, 23.54 (CH_2 Laur. and Myr), 14.62 (CH_3 Laur. and Myr), 9.31 (CH_3 Tea); ^{31}P NMR (243 MHz, CD_3OD - $CDCl_3$, 2:1) δ 0.15, 0.08; HRMS (ESI) *m/z* calcd. for $[M + 2H]^{2+}$ $C_{112}H_{166}N_4O_{23}P_2$ 998.5709, found 998.5722.

2-Deoxy-3-*O*-[(*R*)-3-(hydroxyl)dodecanoyl]-4-*O*-[(2-aminoethoxy)phosphoryl]-2-[(*R*)-3-(hydroxyl)tetradecanoyl-amino]-β-D-glucopyranosyl-(1↔1)-2-deoxy-3-*O*-[(*R*)-3-(hydroxyl)dodecanoyl]-4-*O*-[(2-aminoethoxy)-phosphoryl]-2-[(*R*)-3-(hydroxyl)tetradecanoylamino]-α-D-glucopyranoside (βα-DLAM44)

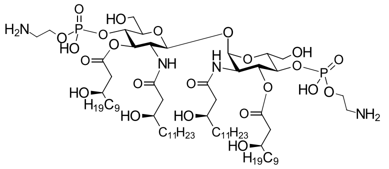

To a stirred solution of **27** (0.005 mmol, 10 mg) in $CHCl_3$ (4 mL), 2,3-dichloro-5,6-dicyano-p-benzoquinone (10 equiv., 0.05 mmol, 11 mg) was added at r.t. and reaction mixture was stirred for 24 h. $CHCl_3$ (20 mL) was added and the reaction mixture was extracted with satd. aq. $NaHCO_3$ (3x5 mL) and water (3x5 mL), the organic phase was filtrated over cotton and the solvent concentrated. The residue was purified by column chromatography on silica gel (1 g), ($CHCl_3$ - MeOH - Et_3N, 5:4:0.1 → 5:6:0.1), products containing fractions were pooled together and concentrated. The residue, dissolved in MeOH – DCM, 1:2 with 10 µL of Et_3N, was subjected to Sephadex LH-20 size exclusion resin support (DCM – MeOH, 1:2) to give **βα-DLAM44** (3 mg, 42 %) as white foam. R_f = 0.4 ($CHCl_3$ - MeOH - H_2O, 5:3:0.5); ^1H NMR (600 MHz, $CDCl_3$ – CD_3OD, 1:3) δ 8.06 (d, 1H, $^3J_{NH,H2}$ = 8.8 Hz, NH), 7.41 (d, 1H, $^3J_{NH′,H2′}$ = 9.0 Hz, NH′), 5.24 (dd, 1H, $^3J_{H2′,H3′}$ = $^3J_{H3′,H4′}$ = 10.0 Hz, H-3′), 5.17 (dd, 1H, $^3J_{H2,H3}$ = $^3J_{H3,H4}$ = 9.8 Hz, H-3), 5.04 (d, 1H, $^3J_{H1′,H2′}$ = 3.4 Hz, H-1′), 4.70 (d, 1H, $^3J_{H1,H2}$ = 8.2 Hz, H-1), 4.29-4.27 (m, 2H, H-4, H-4′), 4.19 (m, 1H, H-2′), 4.05-3.94 (m, 10H, H-2, H-5′, 4xβ$^{\text{Lau and Myr}}$-C*H*, 2xOC*H*$_2$CH$_2$NH$_2$), 3.83-3.79 (m, 4H, H-6, H′-6), 3.46 (m, 1H, H-5), 3.13-3.12 (m, 4H, 2xOCH$_2$C*H*$_2$NH$_2$), 2.54-2.21 (m, 8H, 2xα$^{\text{Lau And Myr}}$-C*H*$_2$), 1.44-1.27 (m, 72H, 36xCH Lau and Myr), 0.89-0.87 (m, 12H, 4xCH_3 Lau and Myr); ^{13}C NMR (151 MHz, $CDCl_3$ – CD_3OD, 1:3) δ 173.43, 173.29, 172.70, 171.94 (4xCO, Lau and Myr), 101.07 (C-1), 98.9 (C-1′), 76.15 (C-5), 74.19 (C-3), 72.42 (C-5′), 71.95 (C-3′), 71.72 (C-4, C-4′), 68.65, 67.84, 67.61 (4xβ$^{\text{Lau and Myr}}$-*C*H), 63.55, 61.68 (2xO*C*H$_2$CH$_2$NH$_2$), 60.53, 60.53 (H-6, H-6′), 53.91 (C-2), 52.13 (C-2′), 43.56, 42.20 (CH_2 Laur. and Myr), 40.27 (2xOCH$_2$*C*H$_2$NH$_2$), 36.98, 31.69, 29.57, 25.43, 22.38, (CH_2 Laur. and Myr), 13.51 (CH_3 Laur. and Myr); All ^{13}C shifts except for those arising from carbonyl carbons were gained from HSQC. Carbonyl ^{13}C shifts were obtained from HMBC; ^{31}P NMR (243 MHz, $CDCl_3$ – CD_3OD, 1:3) δ -1.27, -1.36; MALDI-TOF MS: *m/z* calcd. for calcd. for $[M- H]^-$ $C_{68}H_{131}N_4O_{23}P_2$ 1433.8679, found 1433.686.

3-*O*-[(R)-3-(hydroxyl)Decanoyl]-2-deoxy-4-*O*-[(2-aminoethoxy)phosphoryl]-2-[(R)-3-(hydroxyl)tetradecanoylamino]-β-D-glucopyranosyl-(1↔1)-3-*O*-[(R)-3-(hydroxyl)decanoyl]-2-deoxy-4-*O*-[(2-aminoethoxy)phosphoryl]-2-[(R)-3-(hydroxyl)tetradecanoylamino]-α-D-glucopyranoside (βα-DLAM17)

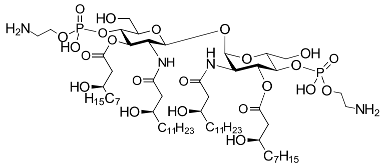

Hydrogenolysis was performed in a pressure-reactor (8.5 bar). A solution of **8** (0.006 mmol, 15 mg) in 2-propanol - H_2O - AcOH (10:0.5:1, 11.5 mL) was stirred with palladium-black (20 mg) under atmosphere of hydrogen (8.5 bar) for 120 h. The mixture was diluted with MeOH - $CHCl_3$ (2:1, 40 mL), the solids were removed by filtration (membrane filter, regenerated cellulose; 0.45 µm) and the filtrate was concentrated. The residue was repeatedly (x 2) redissolved in DCM - MeOH (1:2, 4 mL buffered with Et_3N, 11 µL) and concentrated. The residue was purified by size exclusion chromatography on Sephadex LH-20 support (DCM – MeOH, 1:2) to give **βα-DLAM17** (4 mg, 50 %) as white amorphous solid. R_f = 0.3 ($CHCl_3$ - MeOH – water, 5:3:0.5 with 0.1 % Et_3N); ^{1}H NMR (600 MHz, $CDCl_3$ – CD_3OD, 1:3 D) δ 8.01 (d, 1H, $^3J_{NH,H2}$ = 9.1 Hz, NH), 7.38 (d, 1H, $^3J_{NH',H2'}$ = 8.9 Hz, NH´), 5.21 (dd, 1H, $^3J_{H2',H3'}$ = 10.8 Hz, $^3J_{H3',H4'}$ = 9.1 Hz, H-3´), 5.14 (dd, 1H, $^3J_{H2,H3}$ = $^3J_{H3,H4}$ = 9.8 Hz, H-3), 5.03 (d, 1H, $^3J_{H1',H2'}$ = 3.7 Hz, H-1´), 4.68 (m, 1H, H-1), 4.26-4.23 (m, 2H, H-4, H-4´), 4.22-4.16 (m, 1H, H-2´), 4.06-4.00 (m, 10H, H-2, H-5´, 4x$\beta^{\text{Myr and Capr}}$-*CH,* 2xPOC*H*$_2CH_2NH_2$), 3.87-3.84 (m, 2H, H-6a, H-6´a), 3.80-3.76 (m, 2H, H-6b, H-6´b), 3.43 (m, 1H, H-5), 3.09-3.08 (m, 4H, 2xPOCH$_2$C*H*$_2$NH$_2$), 2.52-2.19 (m, 8H, $\alpha^{\text{Capr and Myr}}$-C*H*$_2$), 1.42-1.25 (m, 64H, 32xCH_2 Myr and Capr), 0.87-0.85 (m, 12H, 4xCH_3- Myr and Capr); ^{13}C NMR (151 MHz, $CDCl_3$ – CD_3OD, 1:3) δ 173.43, 173.29, 172.70, 171.94 (4xCO, Capr and Myr), 101.07 (C-1), 98.87 (C-1´), 75.72 (C-5), 75.95 (C-3), 72.09 (C-4, C-4´, C-5´), 71.80 (C-3´), 68.64, 68.43, 67.80, 67.65 (4x4x$\beta^{\text{Myr and Capr}}$-*C*H), 62.19, 62.05 (2x PO*C*H$_2$CH$_2$NH$_2$), 60.51, 60.30 (C-6, C-6´), 53.83 (C-2), 51.83 (C-2´), 44.70, 43.06, 42.25, 42.10 (CH_2 Capr and Myr), 40.23, 40.19, 40.16, 40.13, 40.08 (2xPOCH$_2$*C*H$_2$NH$_2$), 37.56, 37.50, 36.99, 36.93, 31.74, 31.72, 31.70, 29.59, 29.55, 29.53, 29.52, 29.49, 29.45, 29.42, 29.20, 29.20, 29.18, 29.14, 25.49, 25.43, 25.34, 22.41 (CH_2 Capr and Myr), 13.32 (CH_3 Capr and Myr); ^{31}P NMR (243 MHz, $CDCl_3$ – CD_3OD, 1:3) δ -0.93, -1.01; MALDI-TOF MS: *m/z* calcd. for calcd. for $[M- H]^-$ $C_{64}H_{123}N_4O_{23}P_2$ 1377.8053, found 1377.7.

DLS measurements

Samples of **βα-DLAM44, DA193** and **βα-DLAM44 - cholesterol (1:1)** were prepared as follows**:**

βα-DLAM44; 0.069 mM
βα-DLAM44 (100 µg, 0.069 µmol) was dissolved in 100 µL of DMSO, and water (900 µL) was added.

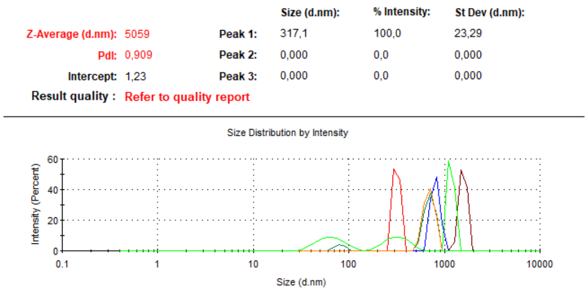

βα-DLAM44; 0.0069 mM
100 µL of **βα-DLAM44** (0.069 mM solution) was diluted with water (900 µL).

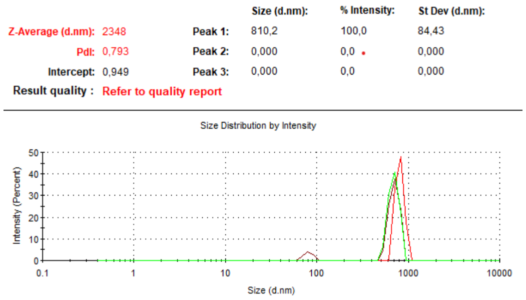

DA193; 0.074 mM
DA193 (100 µg, 0.074 µmol) was dissolved in 100 µL of DMSO and diluted with water (900 µL).

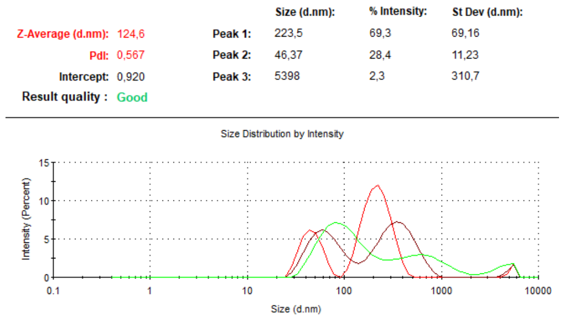

DA193; 0.0074 mM
100 µL of **DA193 (**0.074 mM solution) was diluted with water (900 µL).

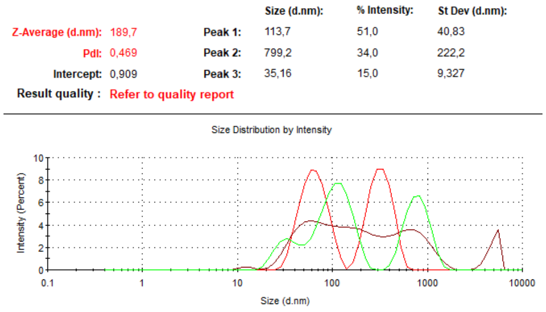

βα-DLAM44–cholesterol, 50:50; 0.069 mM
βα-DLAM44 (100 µg, 0.069 µmol) was dissolved in 0.5 mL of $CHCl_3$–CH_3OH (1:1), and 26 µg of cholesterol was added.
The solvents were evaporated under a stream of argon and then dried in *vacuo*. The residue was redissolved in DMSO (100 µL) and diluted with water (900 µL).

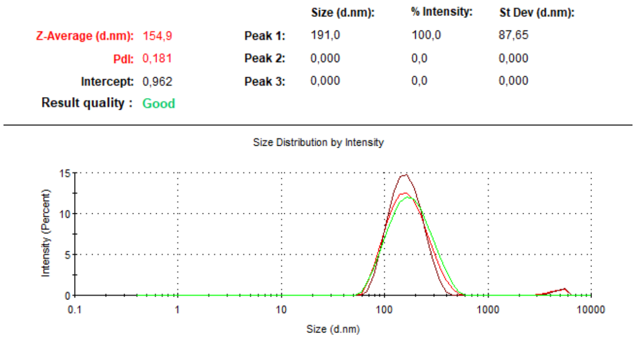

βα-DLAM44–cholesterol, 50:50; 0.0069 mM
100 µL of the **βα-DLAM44–Cholesterol**, 50:50 (0.069 mM stock solution) was diluted with water (900 µL).

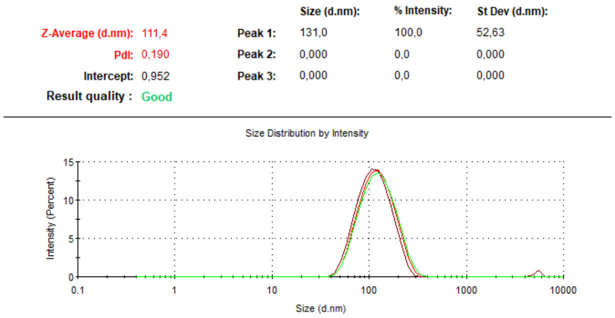

References

1. Benoiton, N. L., On the side-reaction of N-alkylation of amino groups during hydrogenolytic deprotection in alcohol-containing solvents. *Int. J. Pept. Protein Res.* **1993,** *41* (6), 611-611.
2. Li, W.; O'Brien-Simpson, N. M.; Hossain, M. A.; Wade, J. D., The 9-Fluorenylmethoxycarbonyl (Fmoc) Group in Chemical Peptide Synthesis – Its Past, Present, and Future. *Aust. J. Chem.* **2020,** *73* (4), 271-276.
3. Raetz, C. R.; Purcell, S.; Meyer, M. V.; Qureshi, N.; Takayama, K., Isolation and characterization of eight lipid A precursors from a 3- deoxy-D-manno-octylosonic acid-deficient mutant of Salmonella typhimurium. *J. Biol. Chem.* **1985,** *260* (30), 16080-16088.
4. Carpino, L. A.; Sadat-Aalaee, D.; Beyermann, M., Tris(2-aminoethyl)amine as substitute for 4-(aminomethyl)piperidine in the FMOC/polyamine approach to rapid peptide synthesis. *J. Org. Chem.* **1990,** *55* (5), 1673-1675.
5. Radakovics, K.; Battin, C.; Leitner, J.; Geiselhart, S.; Paster, W.; Stöckl, J.; Hoffmann-Sommergruber, K.; Steinberger, P., A Highly Sensitive Cell-Based TLR Reporter Platform for the Specific Detection of Bacterial TLR Ligands. *Front. Immunol.* **2022,** *Volume 12 - 2021*.
6. Schneider, C. A.; Rasband, W. S.; Eliceiri, K. W., NIH Image to ImageJ: 25 years of image analysis. *Nature Methods* **2012,** *9* (7), 671-675.

NMR spectra of synthetic compounds

1, ^{1}H-NMR, ^{1}H NMR (300 MHz, CD_2Cl_2)

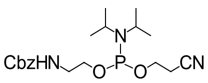

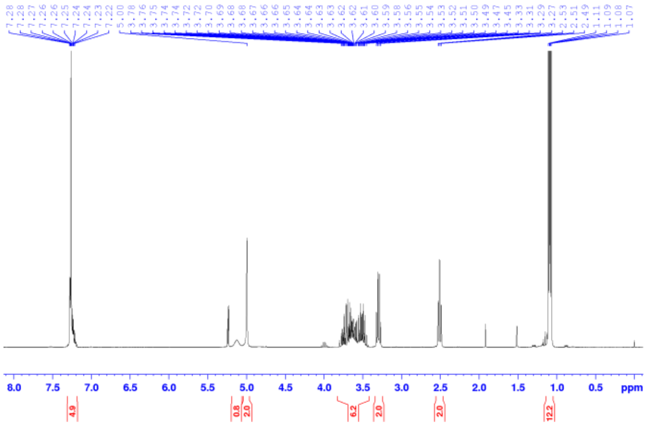

1, ^{31}P NMR (121 MHz, CD_2Cl_2)

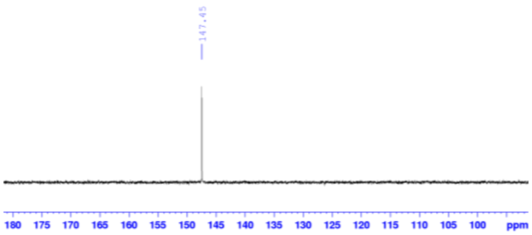

1, ^{13}C NMR (75 MHz, CD_2Cl_2)

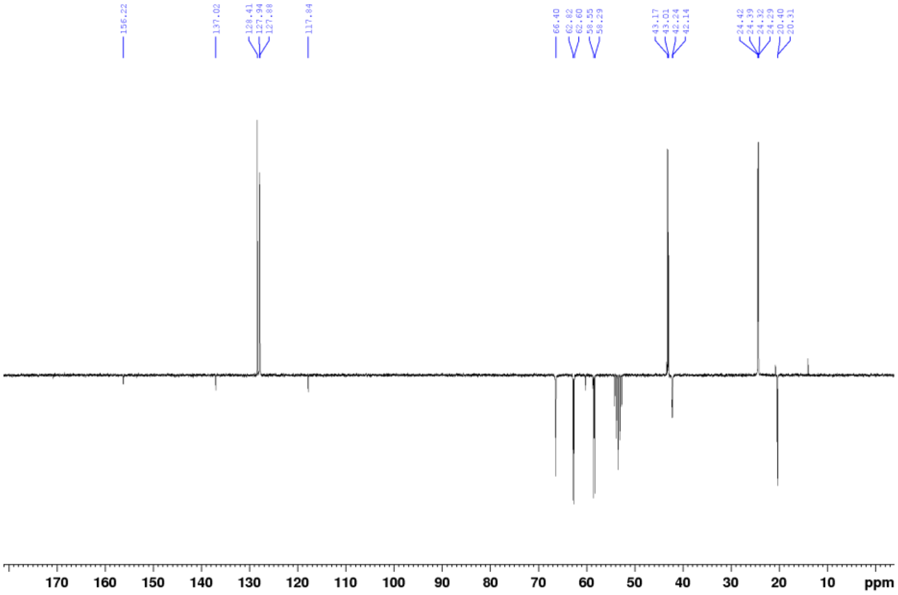

5, ^{1}H NMR (600 MHz, $CDCl_3$ – CD_3OD, 1:2)

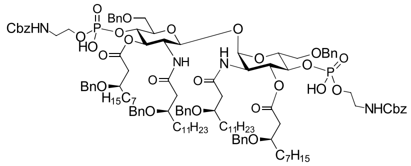

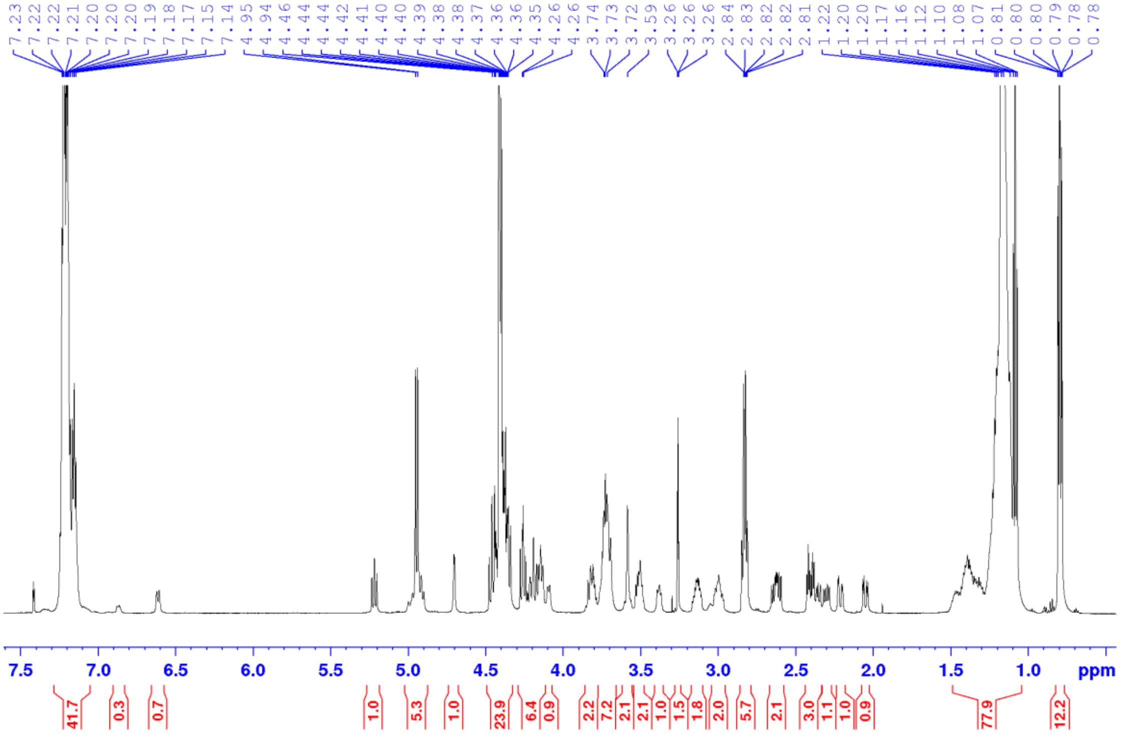

5, ^{13}C NMR (151 MHz, $CDCl_3$ – CD_3OD, 1:2)

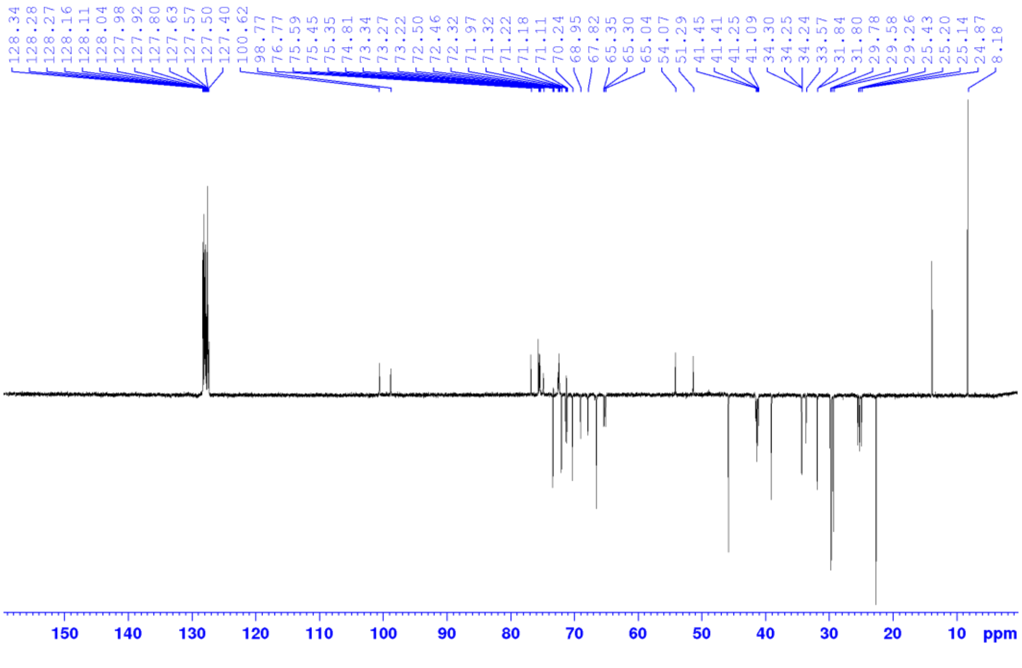

5, ^{31}P NMR (243 MHz, $CDCl_3$ – CD_3OD, 1:2)

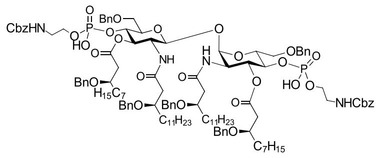

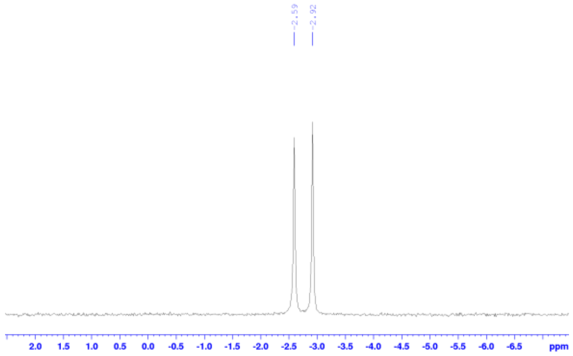

6 (by-product), ^{1}H NMR (600 MHz, $CDCl_3$ – CD_3OD, 1:3)

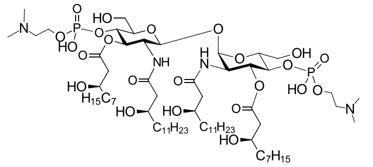

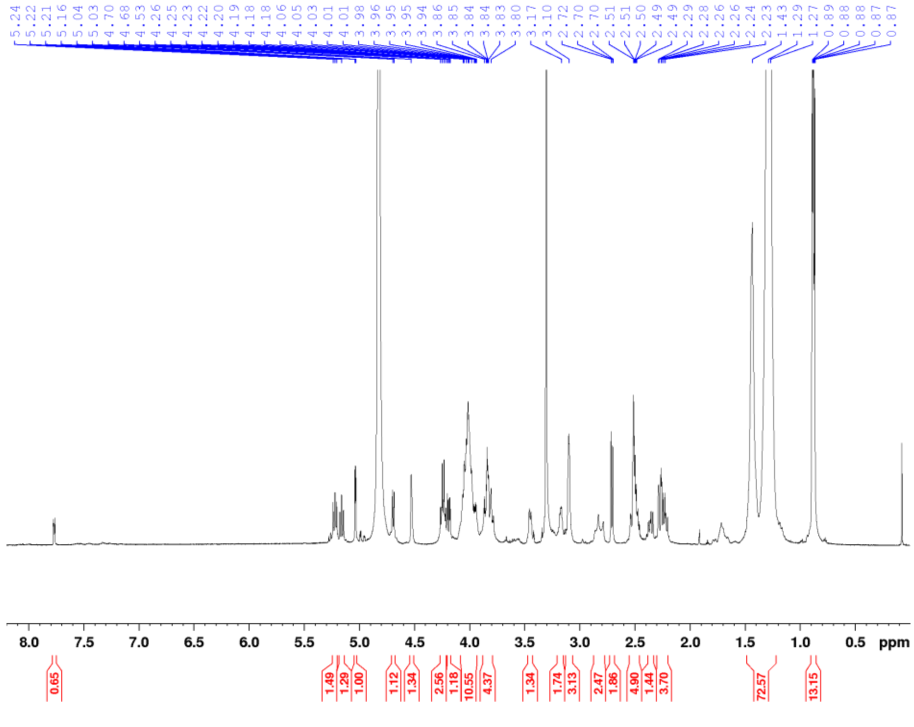

6 (by-product),, ^{1}H-^{13}C HSQC NMR (600/151 MHz, $CDCl_3 - CD_3OD$, 1:3)

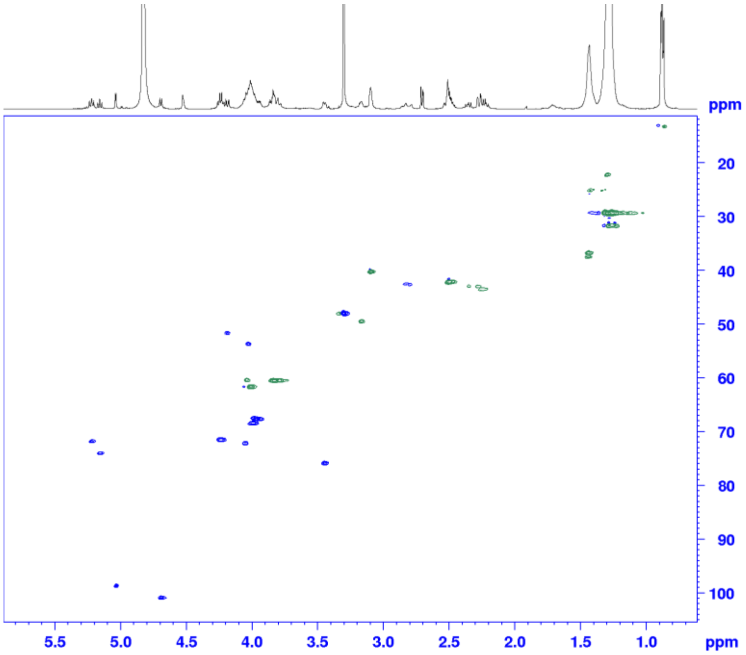

8, ^{1}H NMR (600 MHz, $CDCl_3 - CD_3OD$ = 1:3)

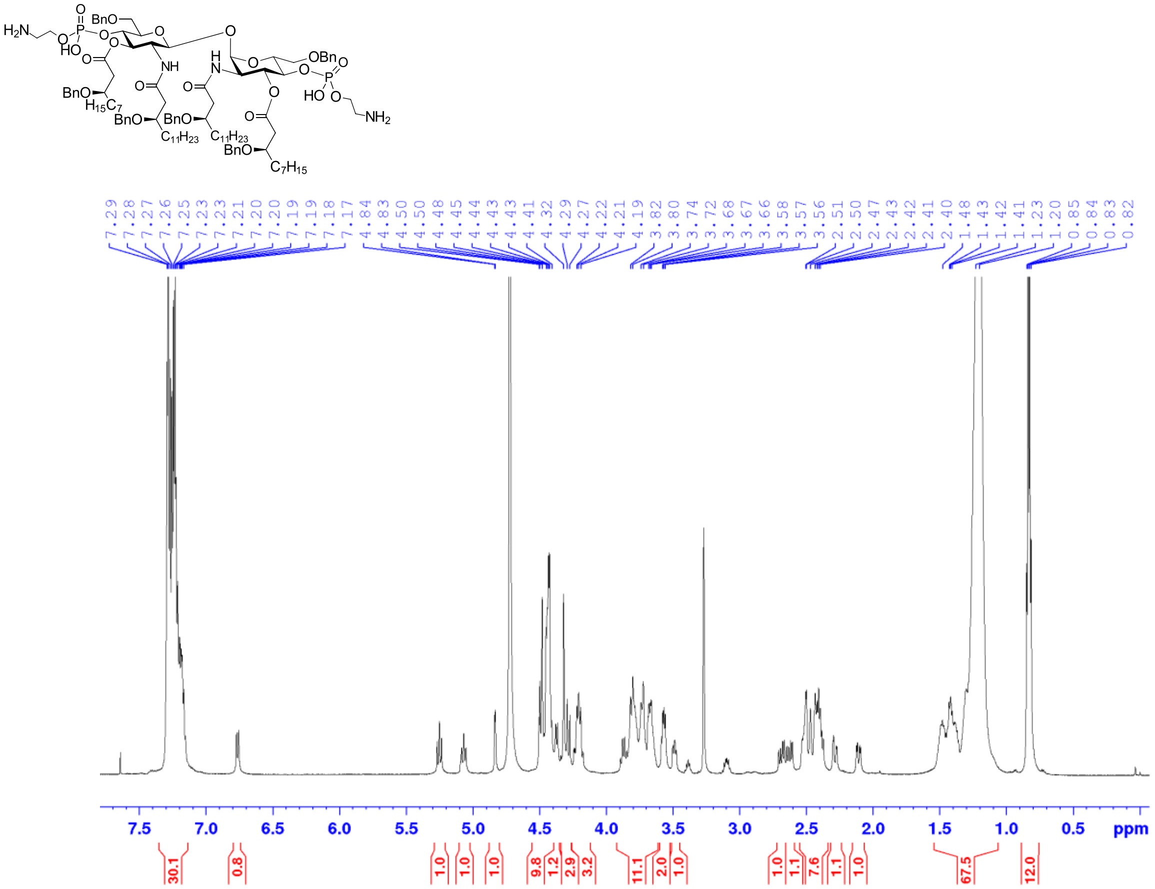

8, ^{13}C NMR (151 MHz, $CDCl_3 – CD_3OD$ = 1:3)

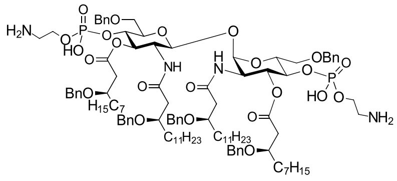

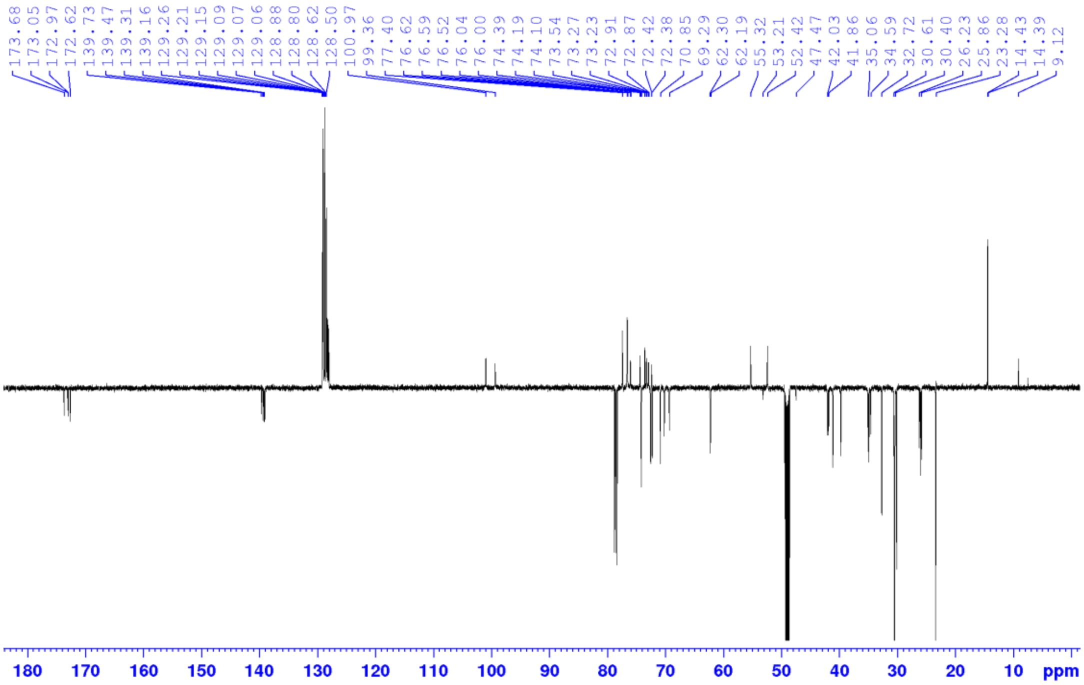

8, ^{31}P NMR (243 MHz, $CDCl_3 – CD_3OD$ = 1:3)

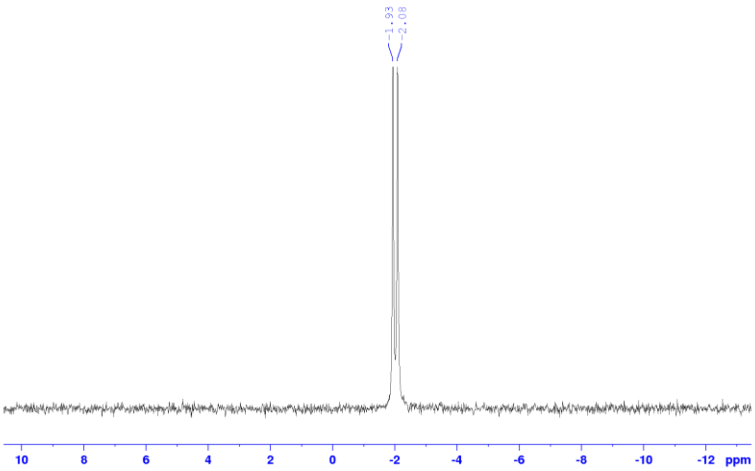

9, ^{1}H NMR (600 MHz, $CDCl_3$ – CD_3OD, 3:1)

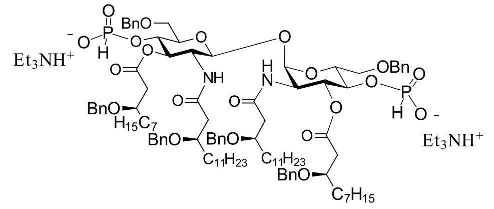

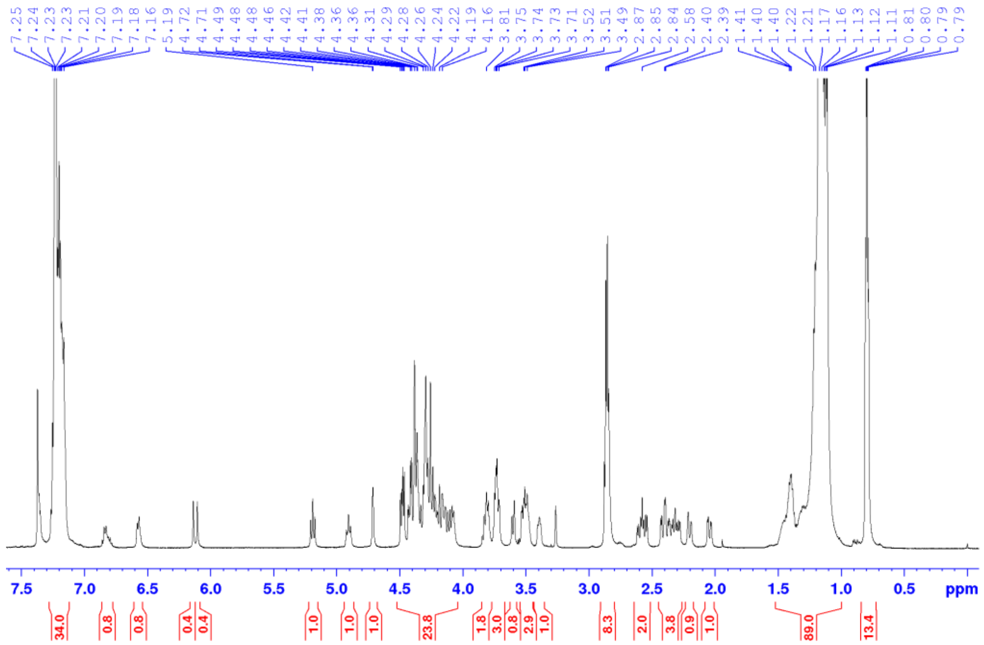

9, ^{13}C NMR (151 MHz, $CDCl_3$ – CD_3OD, 3:1)

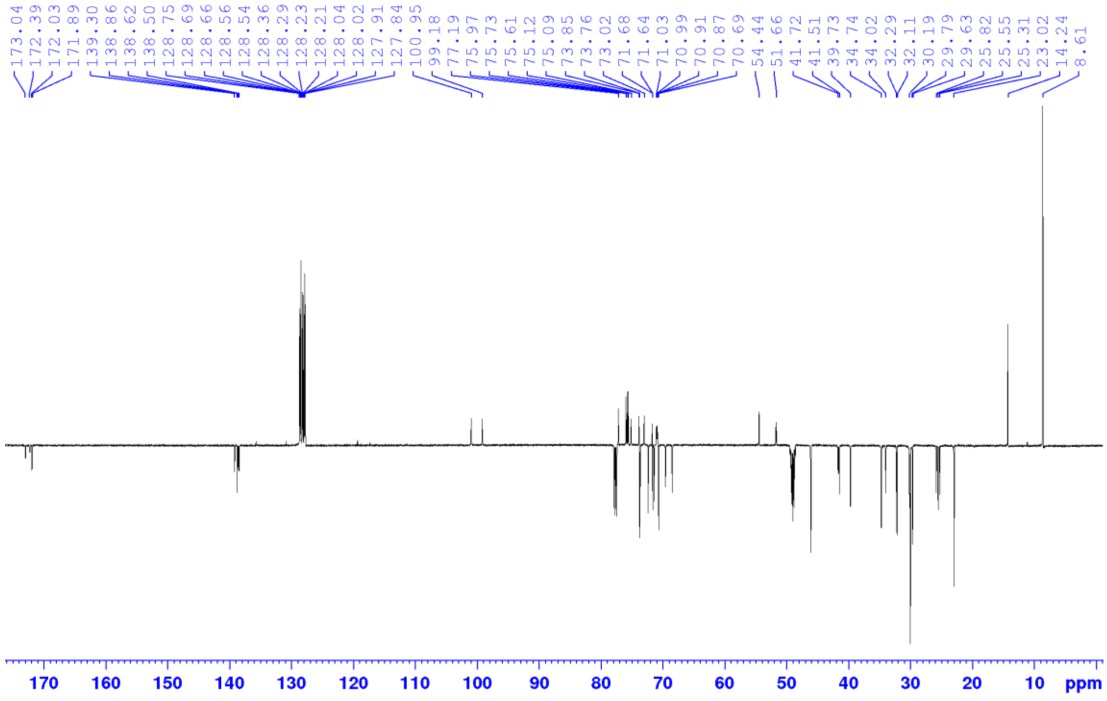

9, ^{31}P NMR H-coupled and H-decoupled (243 MHz, $CDCl_3 - CD_3OD$, 3:1)

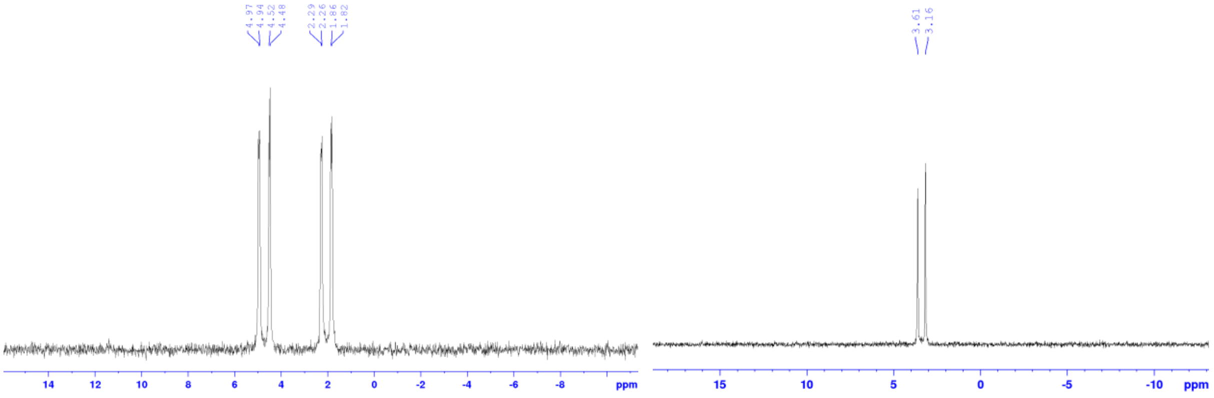

11, ^{1}H NMR (600 MHz, $CDCl_3 - CD_3OD$, 1:3)

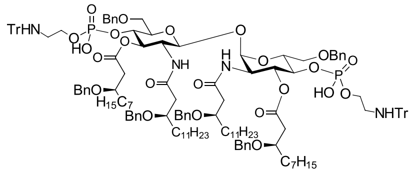

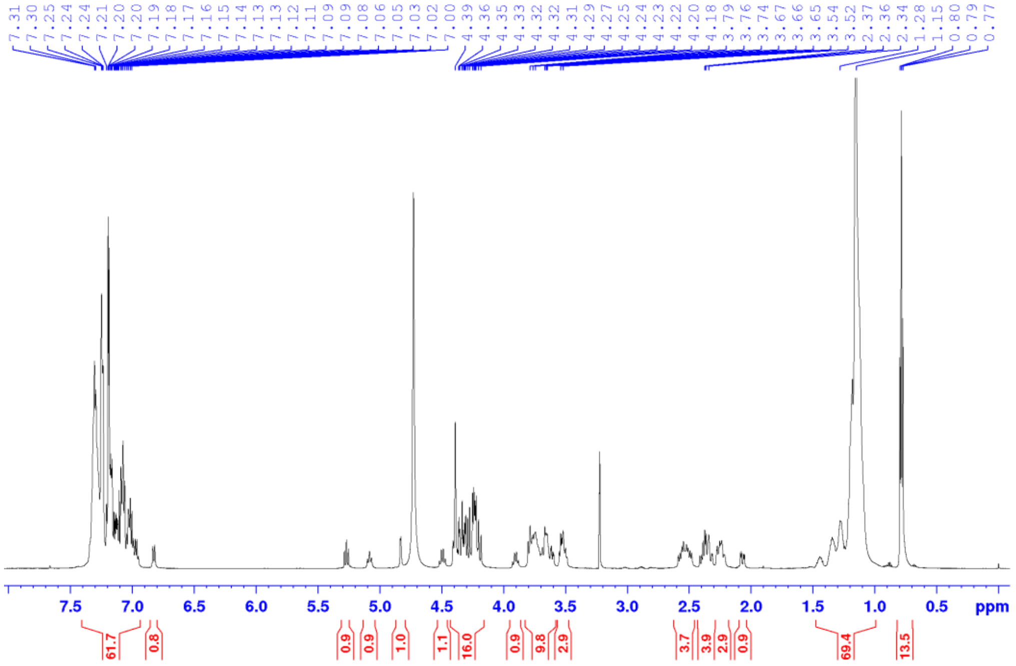

11, ^{13}C NMR (151 MHz, $CDCl_3$ – CD_3OD, 1:3)

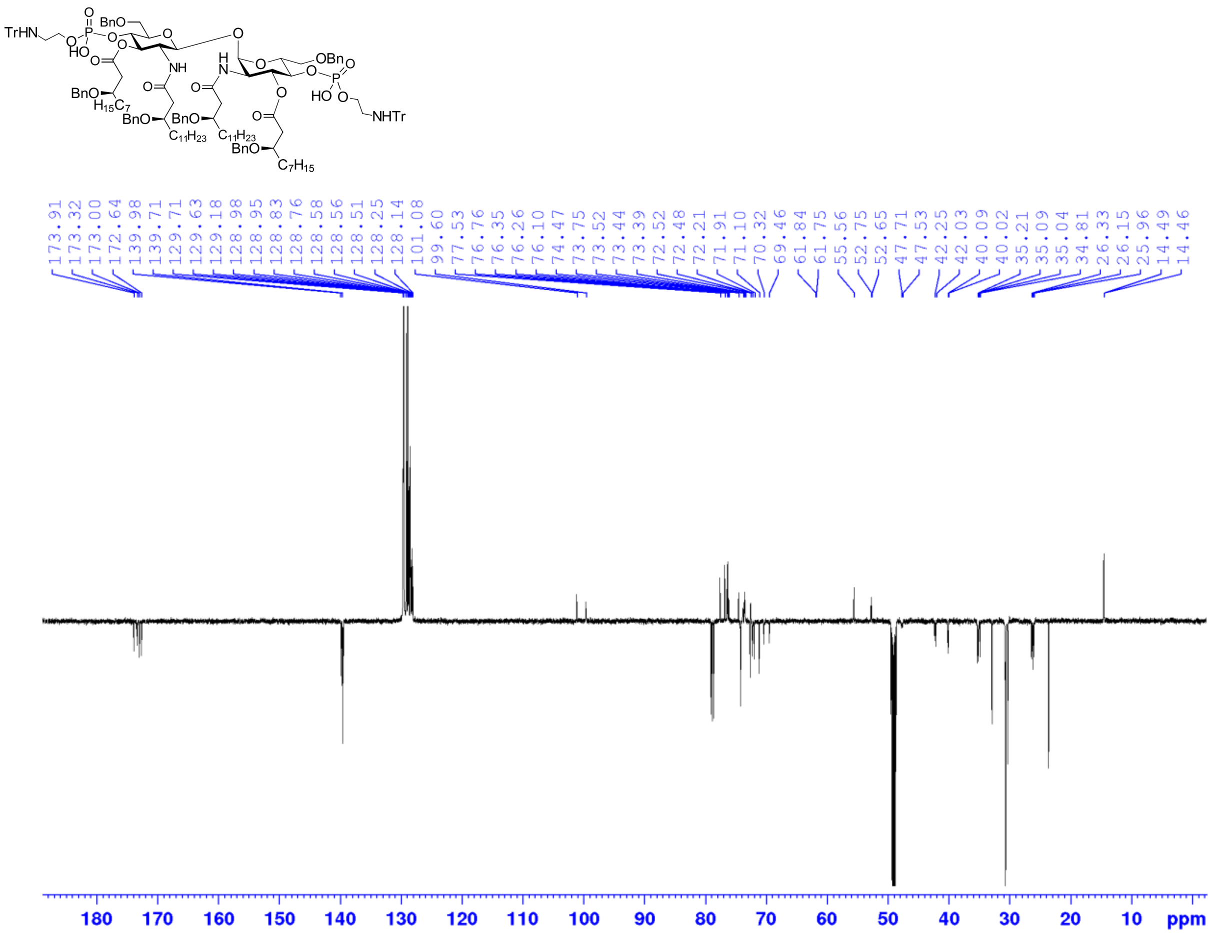

11, ^{31}P NMR (243 MHz, $CDCl_3$ – CD_3OD, 1:3), (243 MHz, CD_2Cl_2 – CD_3OD, 1:3)

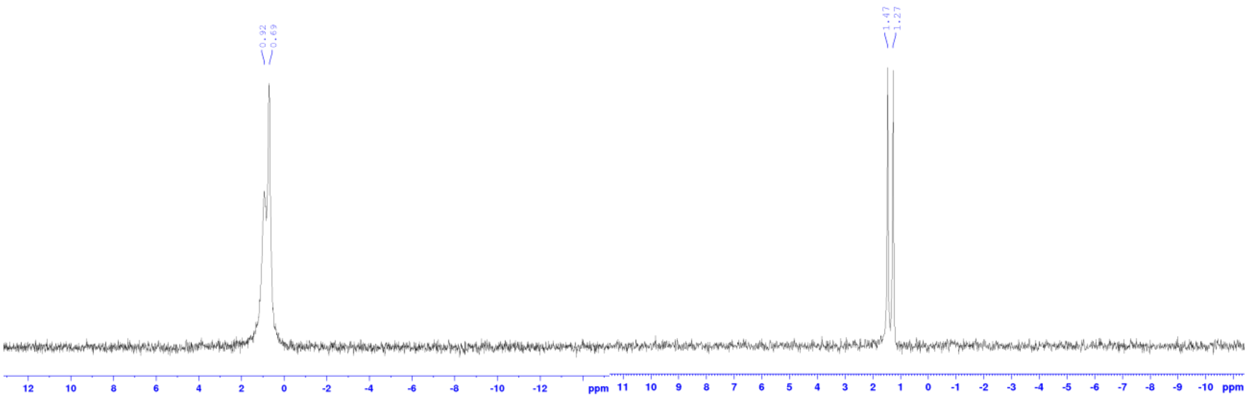

12, ^{1}H NMR (600 MHz, $CDCl_3$ – CD_3OD, 1:3)

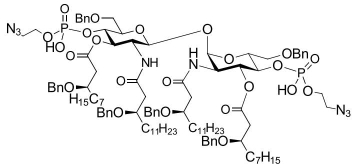

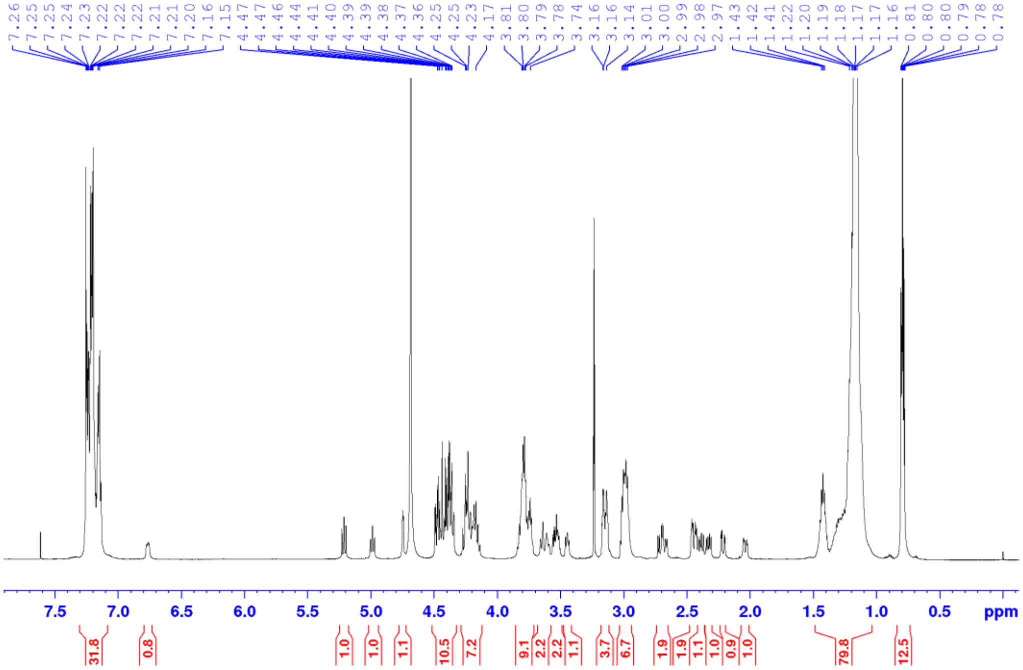

12, ^{13}C NMR (151 MHz, $CDCl_3$ – CD_3OD, 1:3)

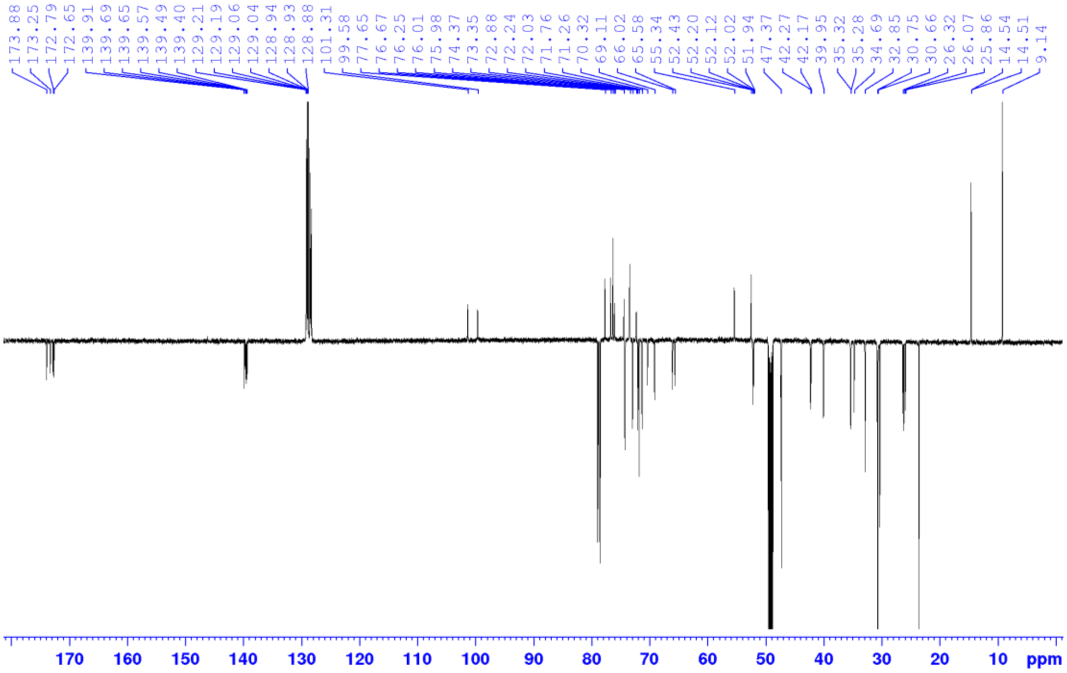

12, ^{31}P NMR (243 MHz $CDCl_3$ – CD_3OD, 1:3)

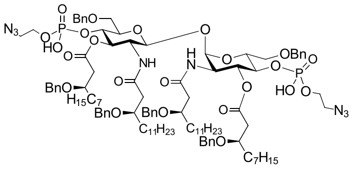

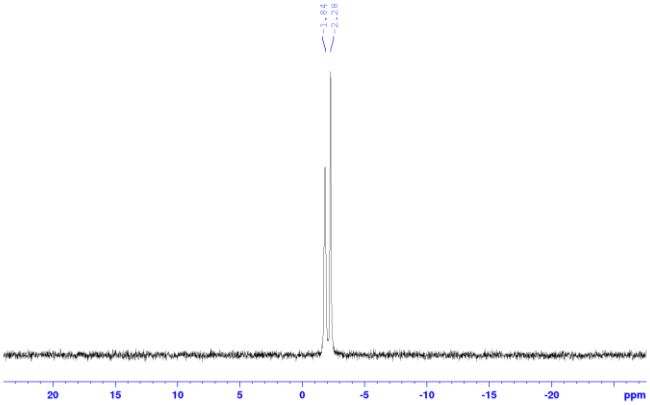

15, ^{1}H NMR (300 MHz, $CDCl_3$)

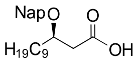

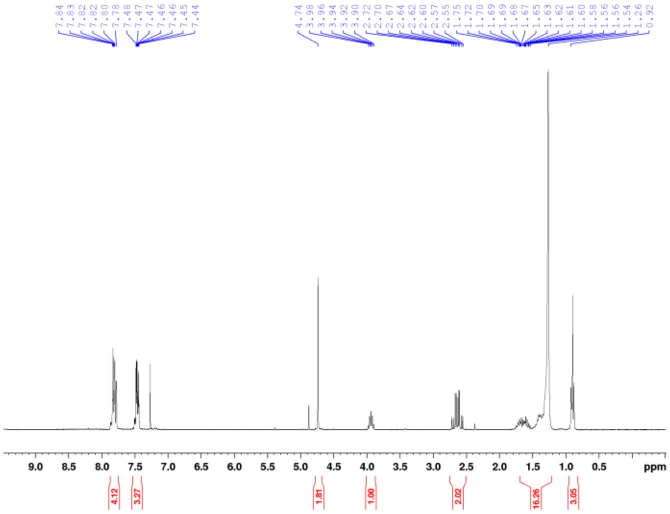

15, ^{13}C NMR (75 MHz, $CDCl_3$)

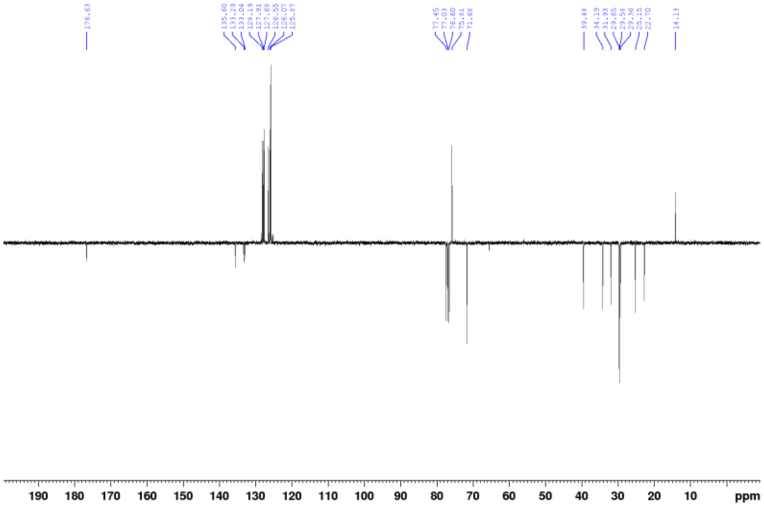

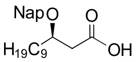

16, ^{1}H NMR (300 MHz, $CDCl_3$)

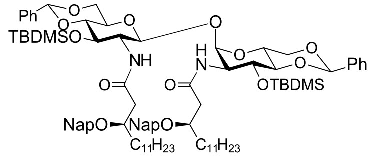

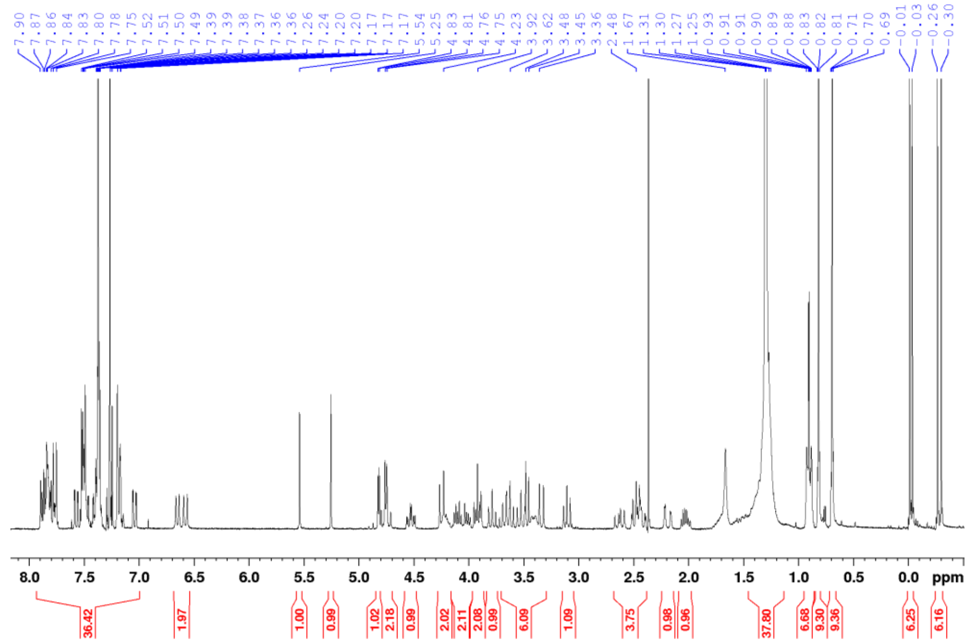

16, ^{13}C NMR (75 MHz, $CDCl_3$)

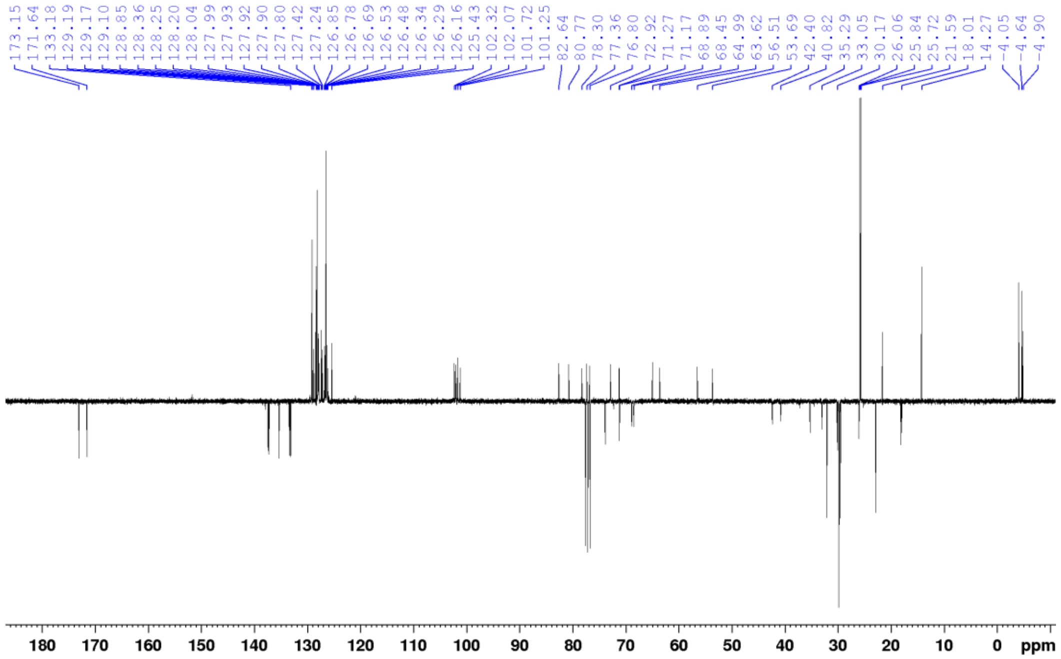

17, ^{1}H NMR (300 MHz, $CDCl_3$)

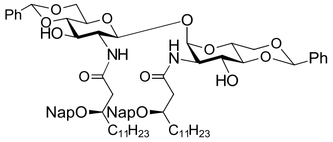

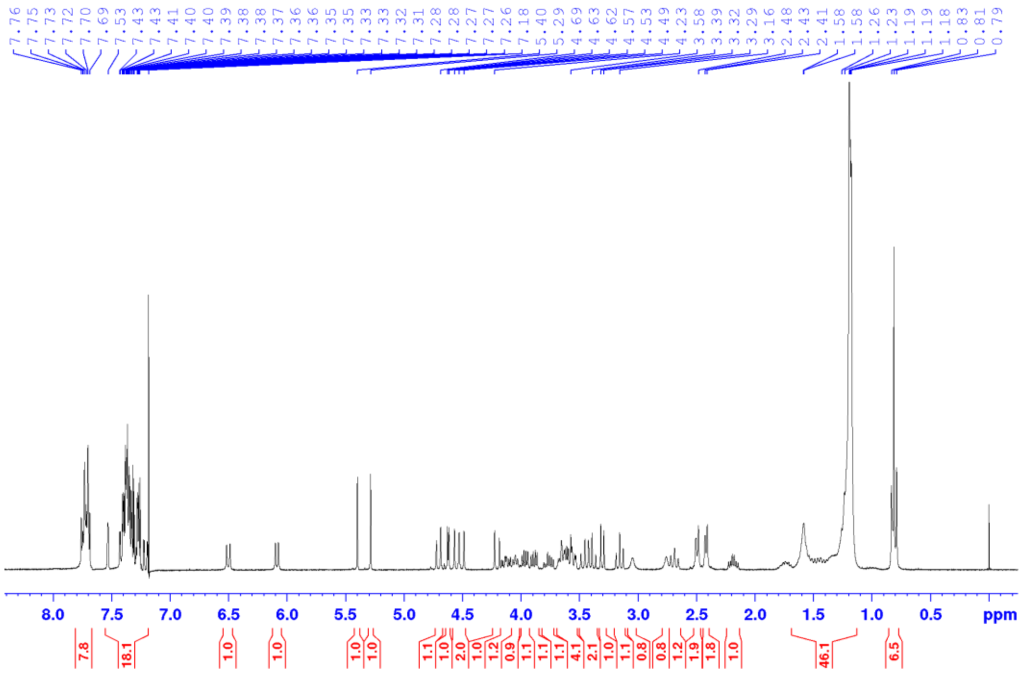

17, ^{13}C NMR (75 MHz, $CDCl_3$)

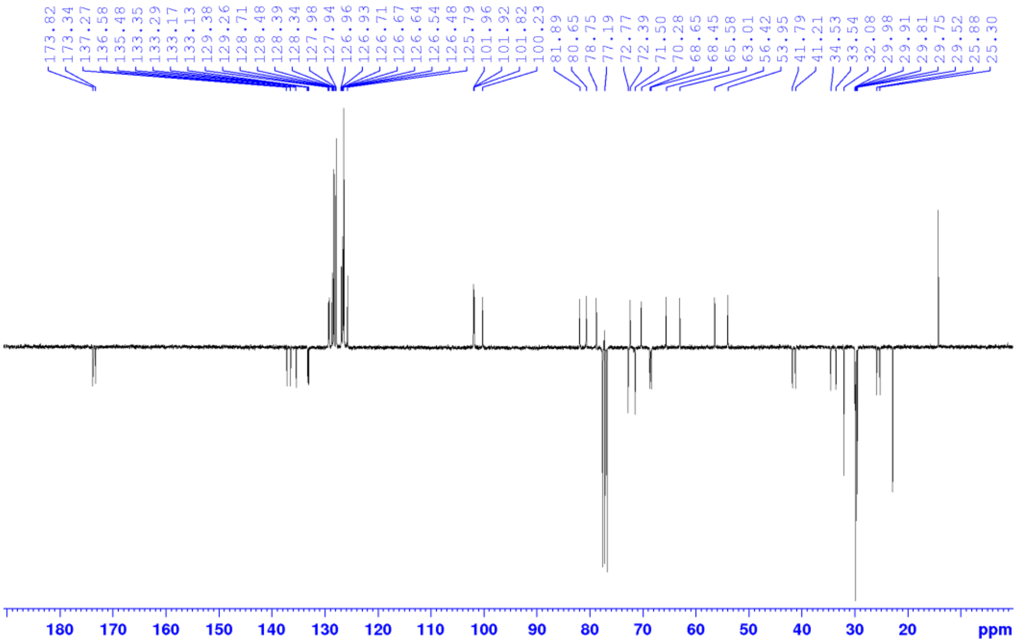

18, ^{1}H NMR (300 MHz, $CDCl_3$)

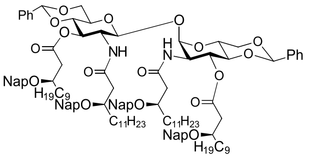

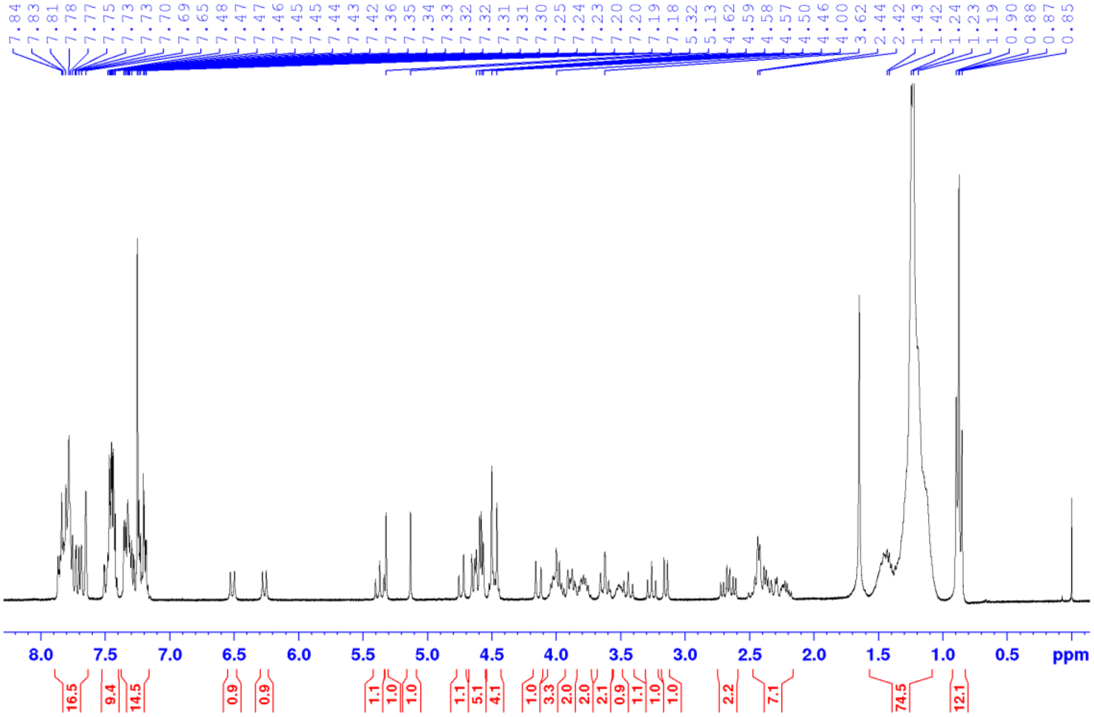

18, ^{13}C NMR (75 MHz, $CDCl_3$)

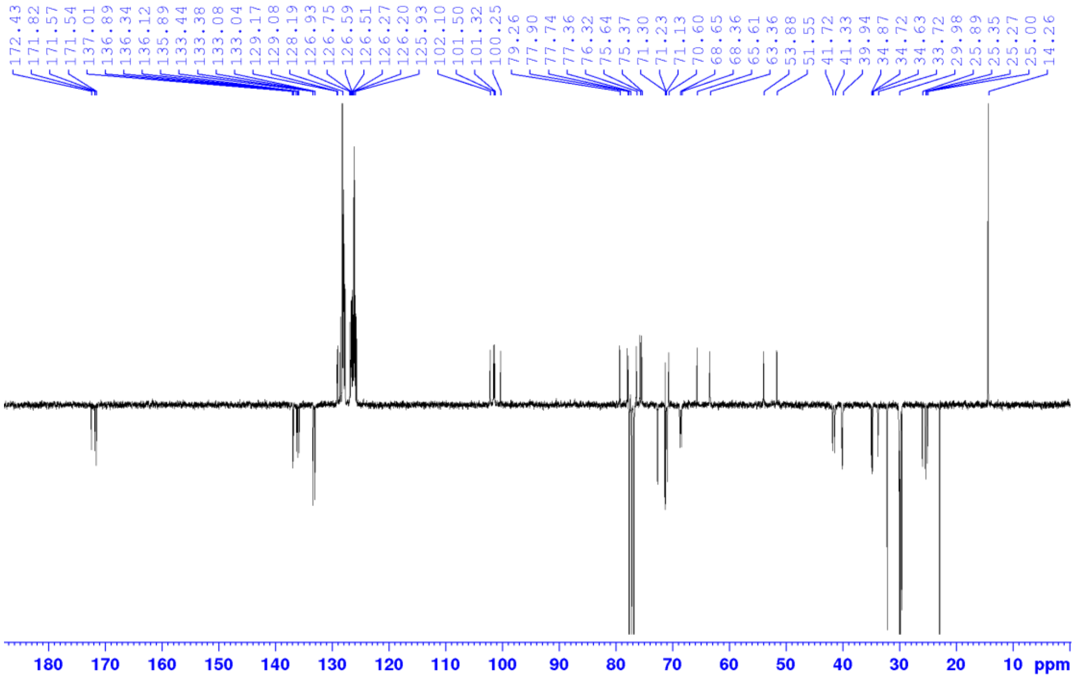

19, ^{1}H NMR (600 MHz, $CDCl_3$)

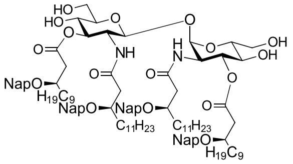

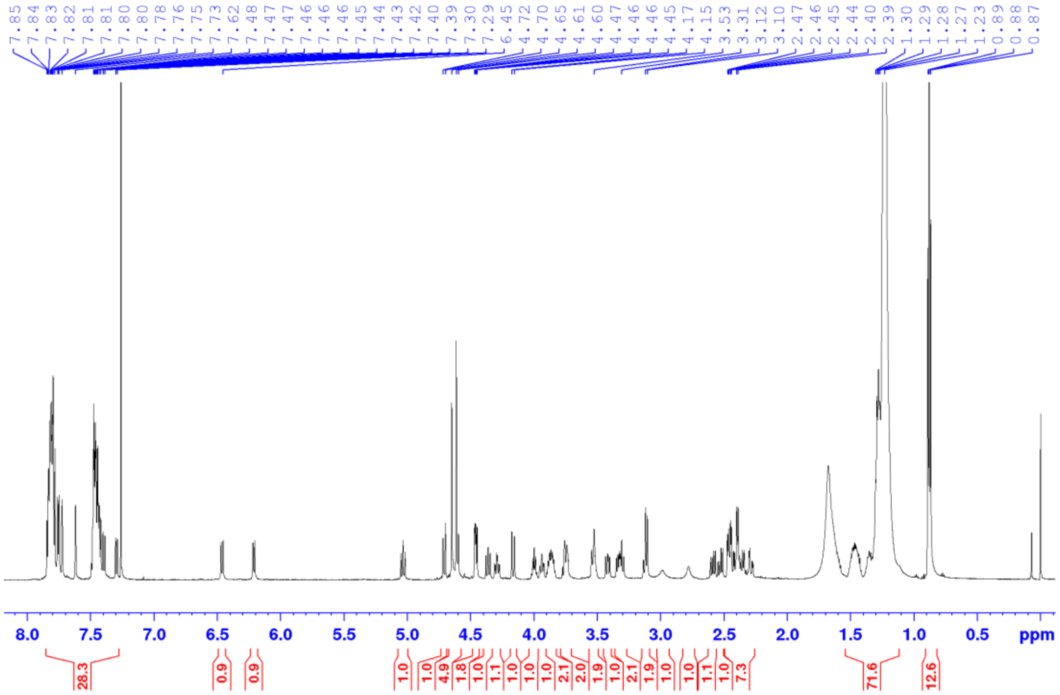

19, ^{13}C NMR (151 MHz, $CDCl_3$)

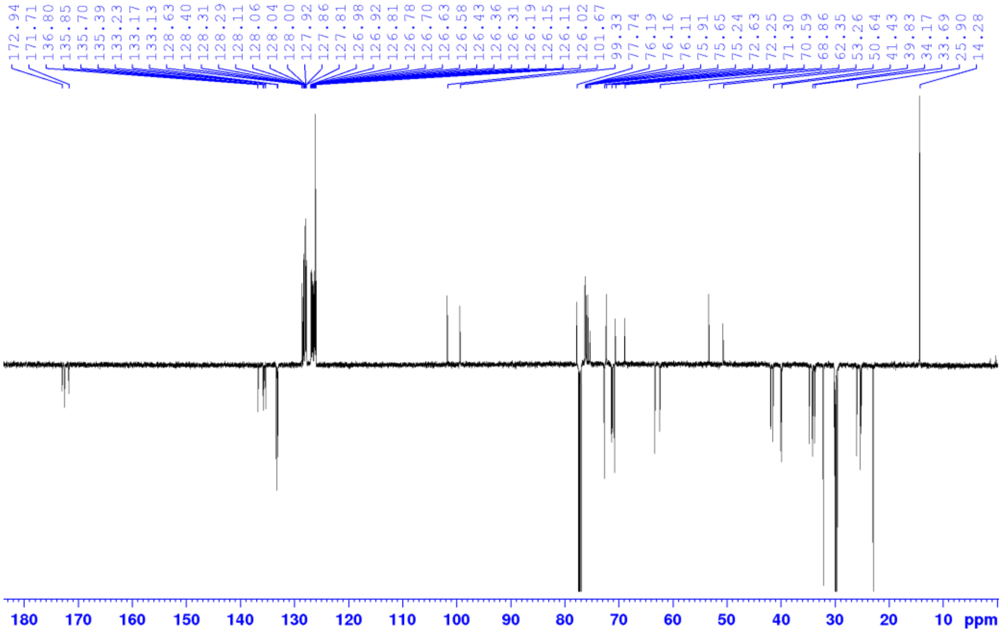

20, ^{1}H NMR (600 MHz, $CDCl_3$)

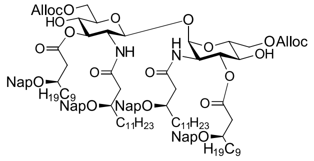

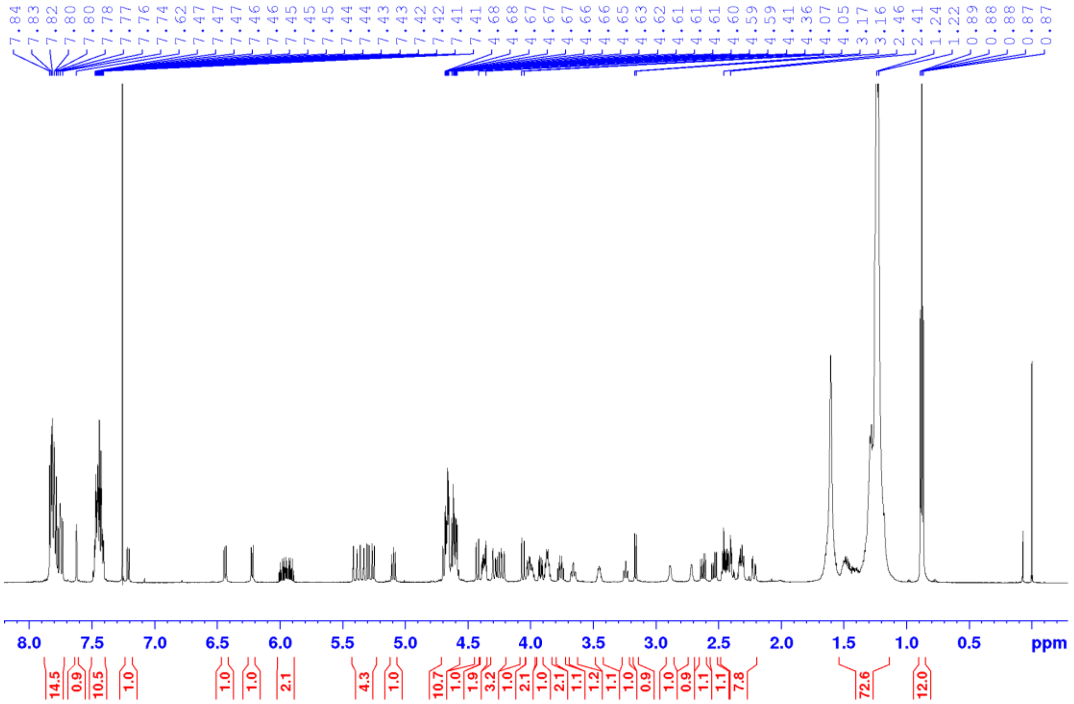

20, ^{13}C NMR (151 MHz, $CDCl_3$)

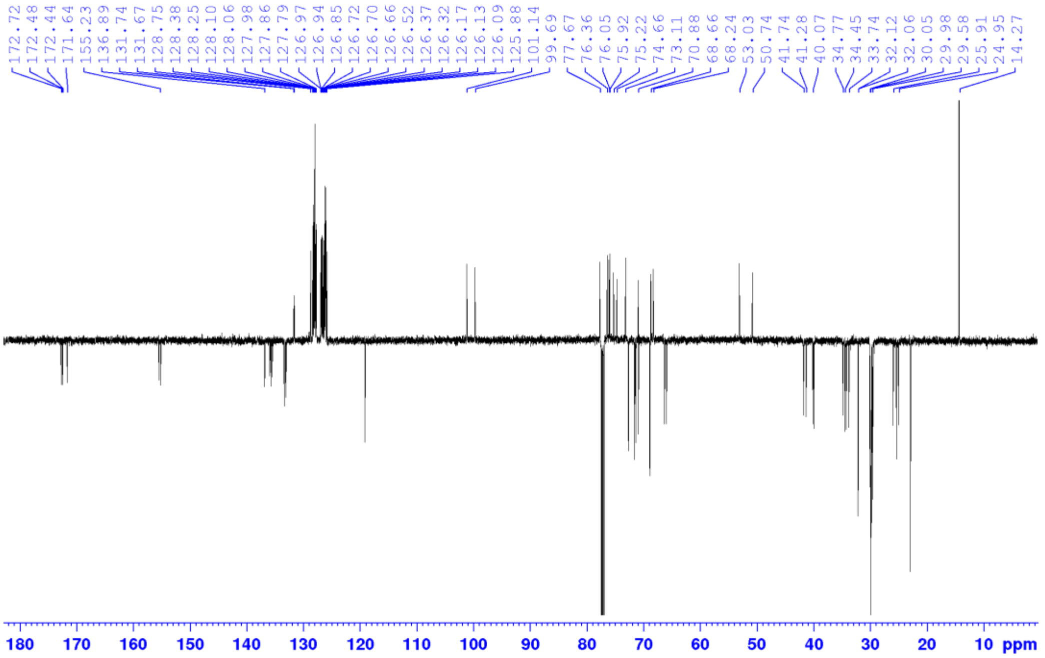

21, ^{1}H NMR (600 MHz, acetone-d_6)

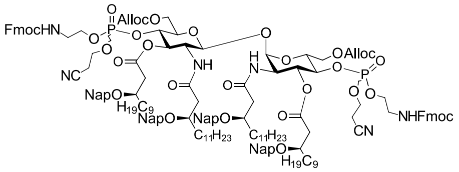

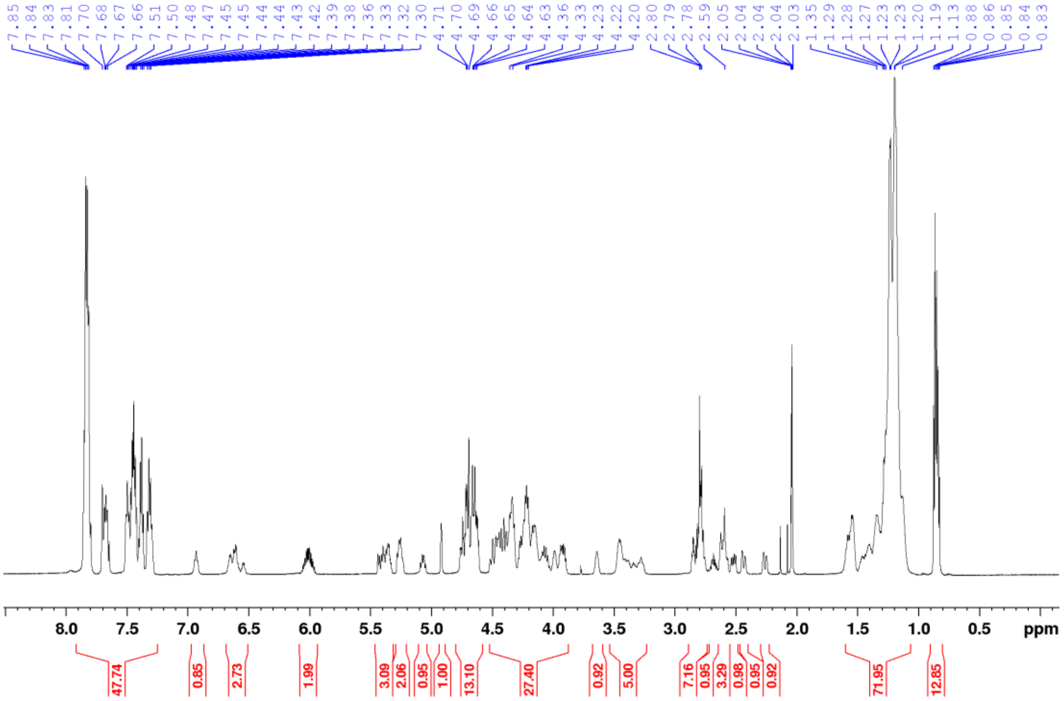

21, ^{13}C NMR (151 MHz, acetone-d_6)

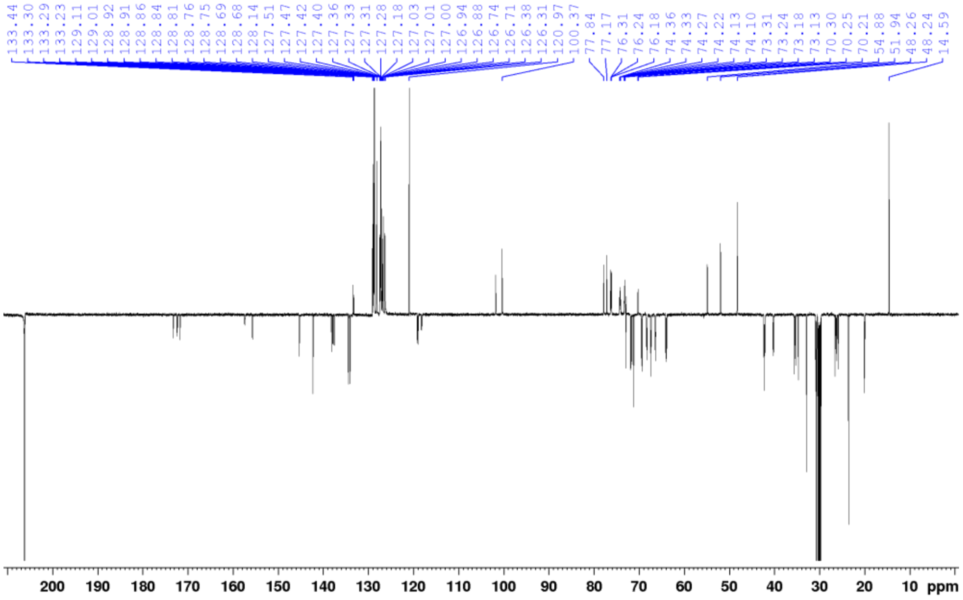

21, ^{31}P NMR (243 MHz, acetone-d$_6$)

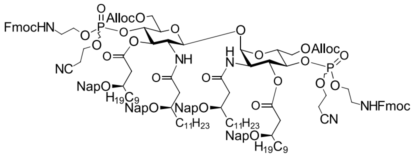

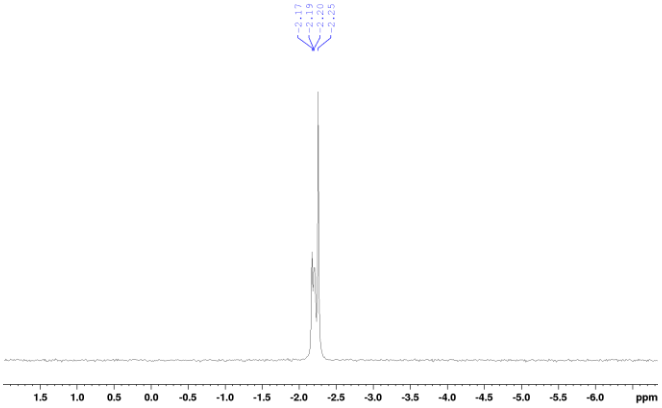

22, ^{1}H NMR (600 MHz, CD_3OD)

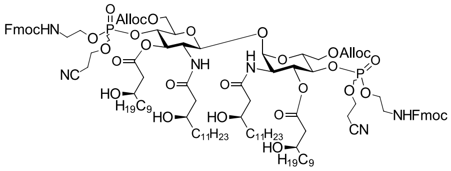

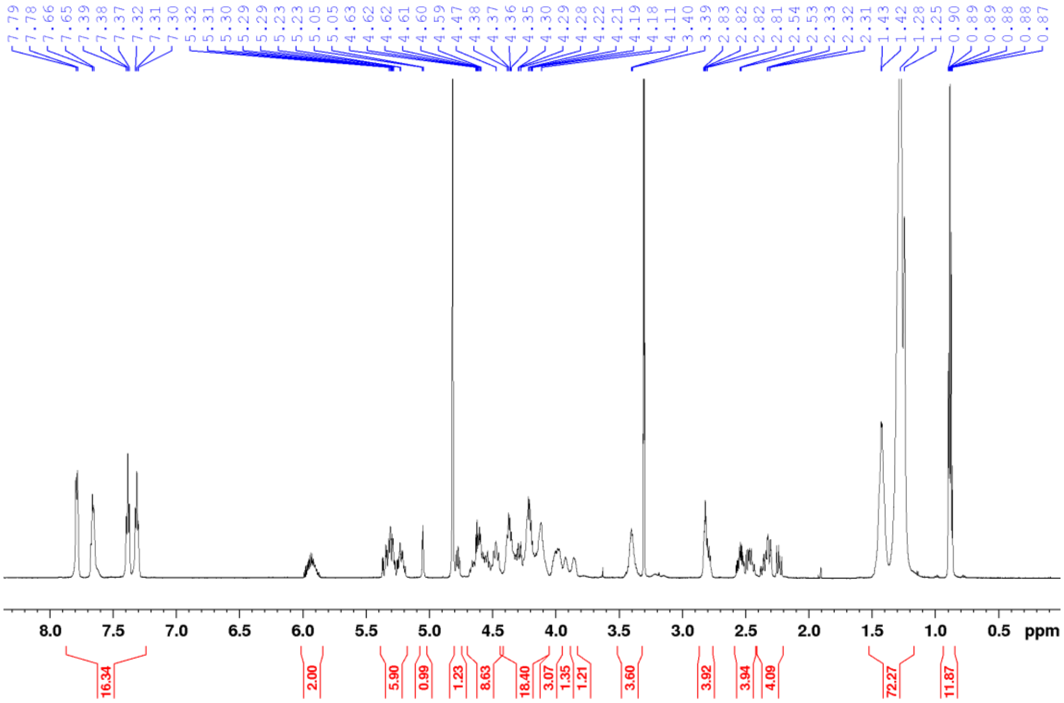

22, ^{13}C NMR (151 MHz, CD_3OD)

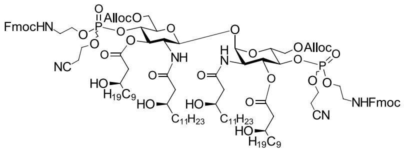

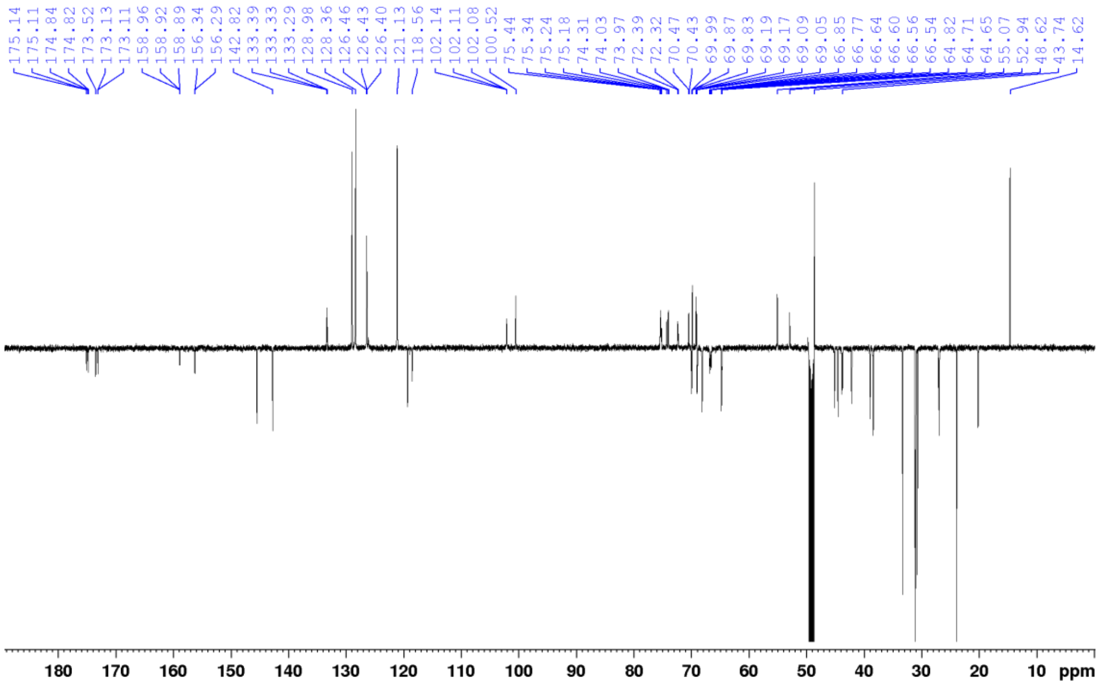

22, ^{31}P NMR (243 MHz, CD_3OD)

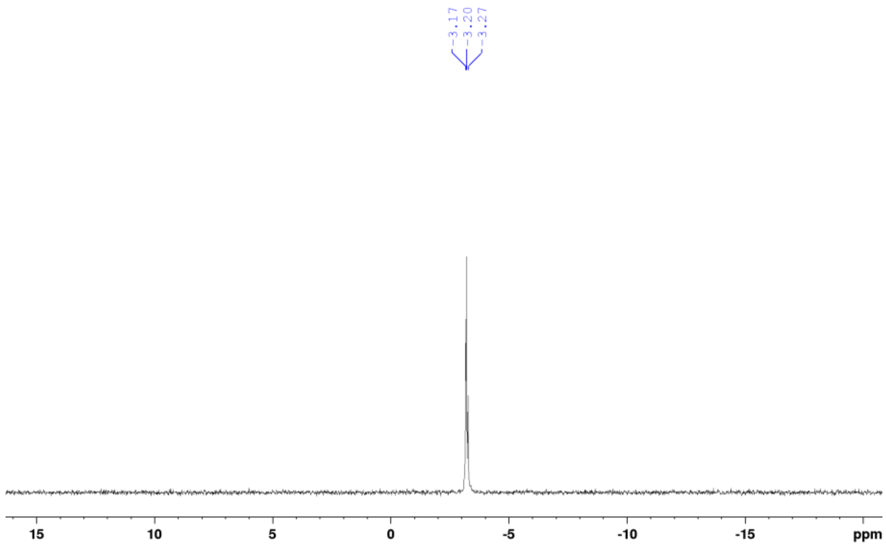

23, ^{1}H NMR (600 MHz, CD_3OD - $CDCl_3$, 3:1;)

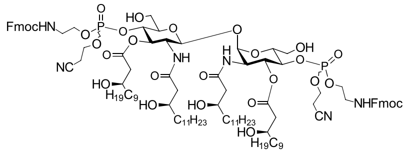

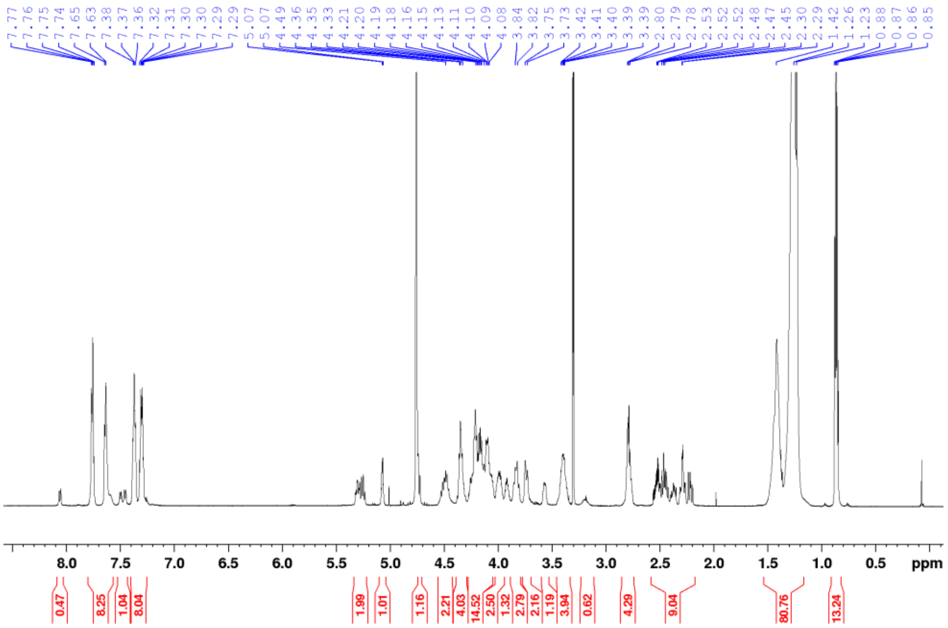

23, ^{13}C NMR (151 MHz, CD_3OD - $CDCl_3$, 3:1;)

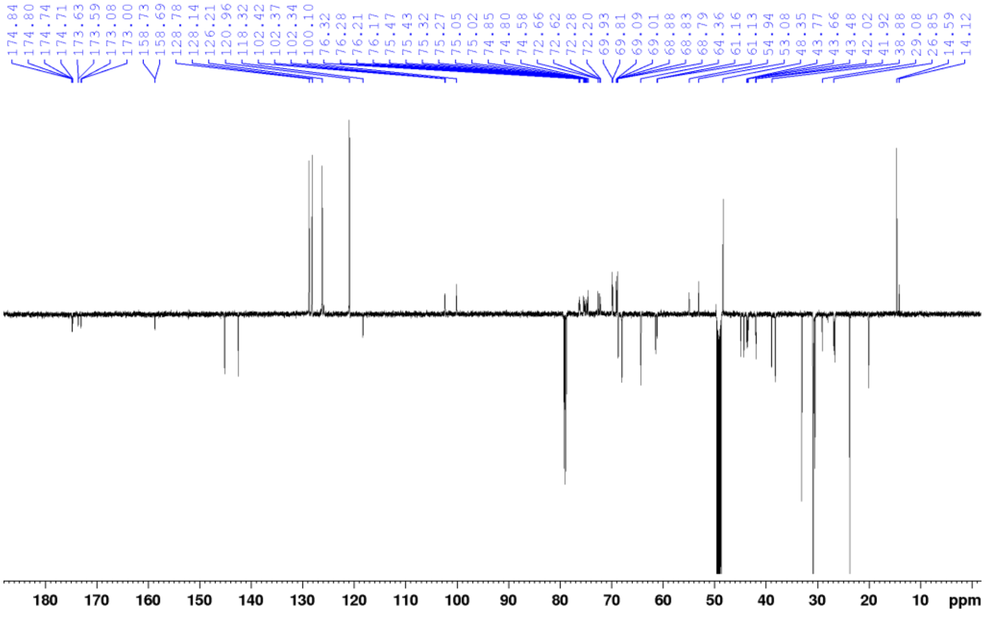

23, ^{31}P NMR (243 MHz, CD_3OD - $CDCl_3$, 3:1)

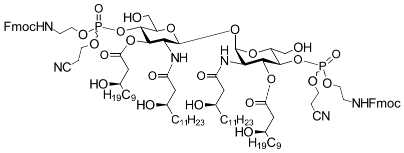

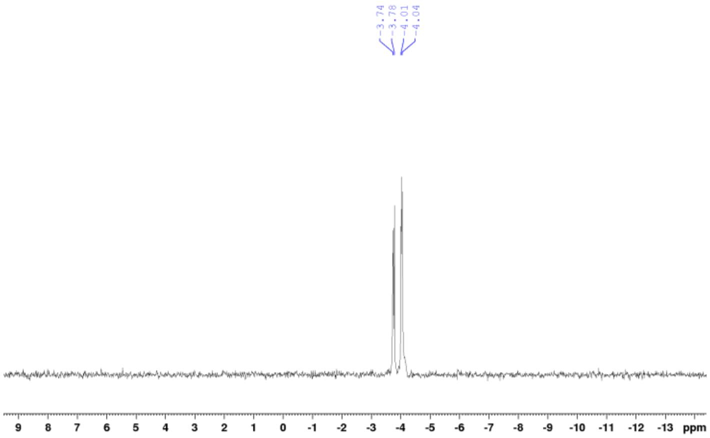

24 (by-product), ^{1}H NMR (600 MHz, $CDCl_3$ – CD_3OD, 1:3)

^{1}H-^{13}C HSQC (600/151 MHz, $CDCl_3$ – CD_3OD, 1:3)

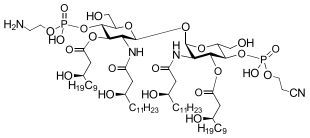

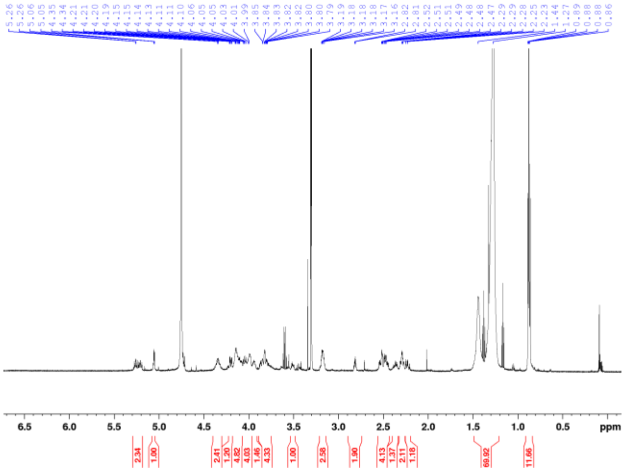

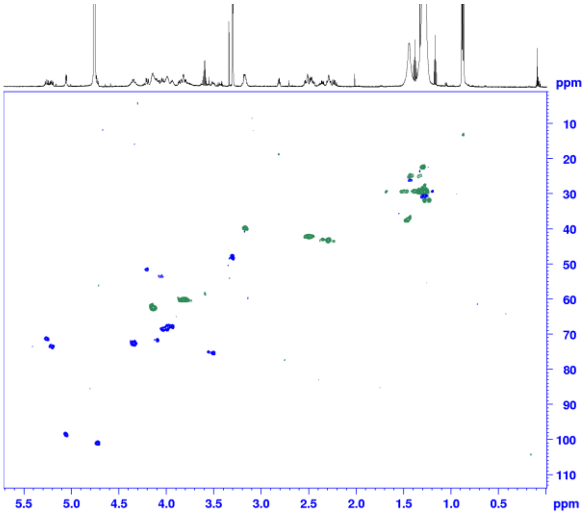

27, ^{1}H NMR (600 MHz, CD_3OD - $CDCl_3$, 2:1)

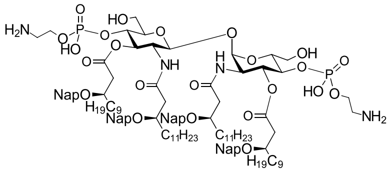

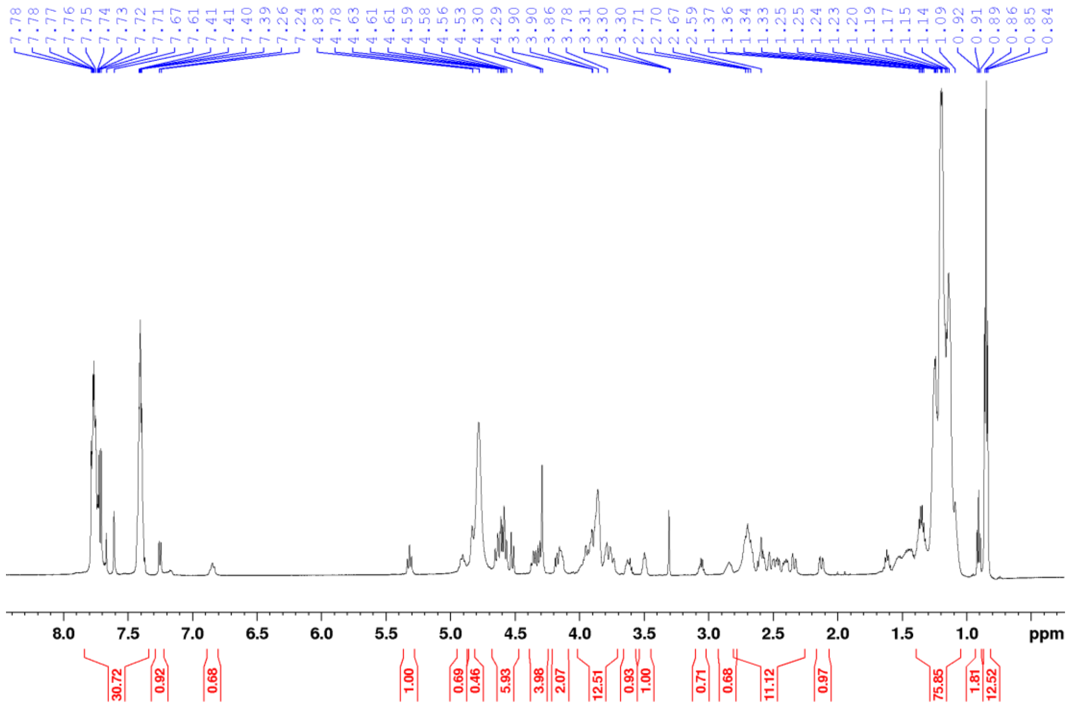

27, ^{13}C NMR (151 MHz, CD_3OD - $CDCl_3$, 2:1)

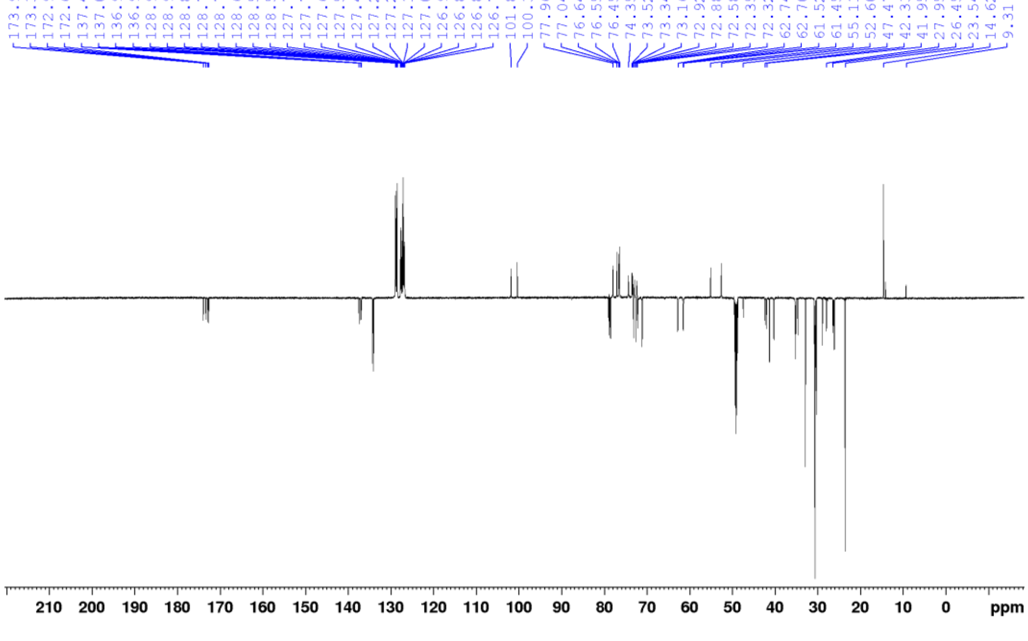

27, ^{1}H-^{13}C HSQC (600/151 MHz, CD_3OD - $CDCl_3$, 2:1)

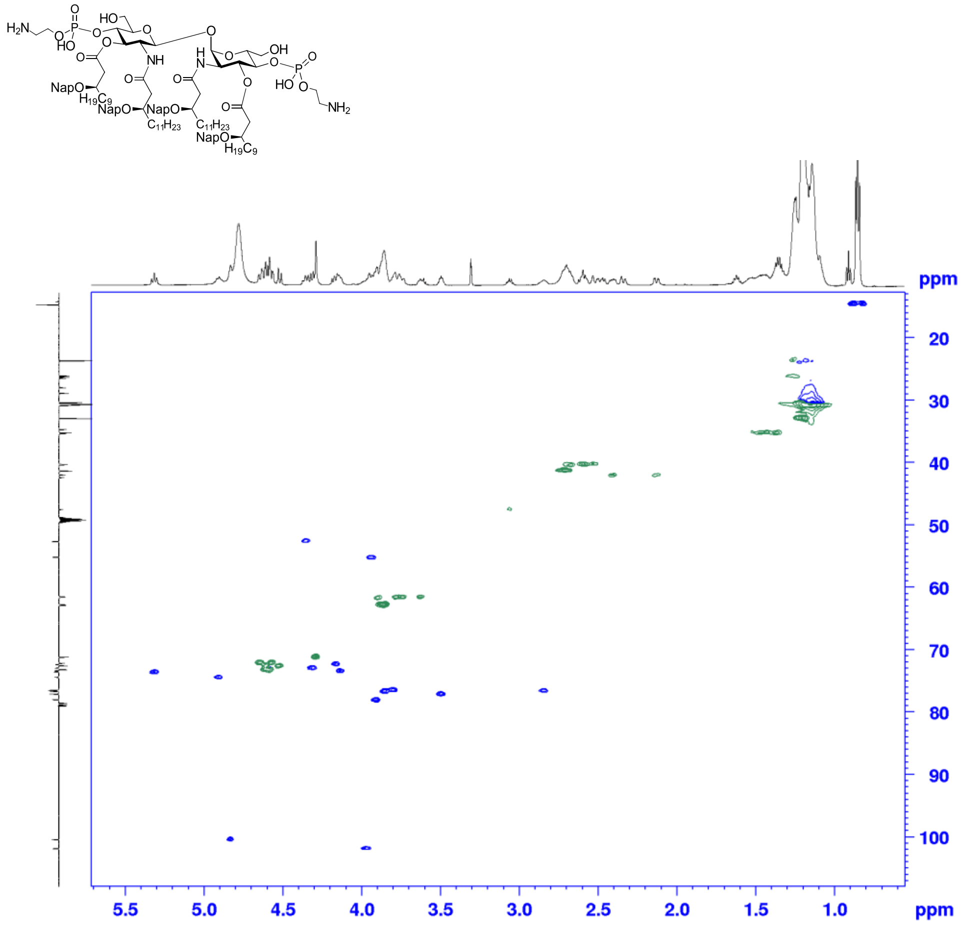

27, ^{31}P NMR (243 MHz, CD_3OD - $CDCl_3$, 2:1)

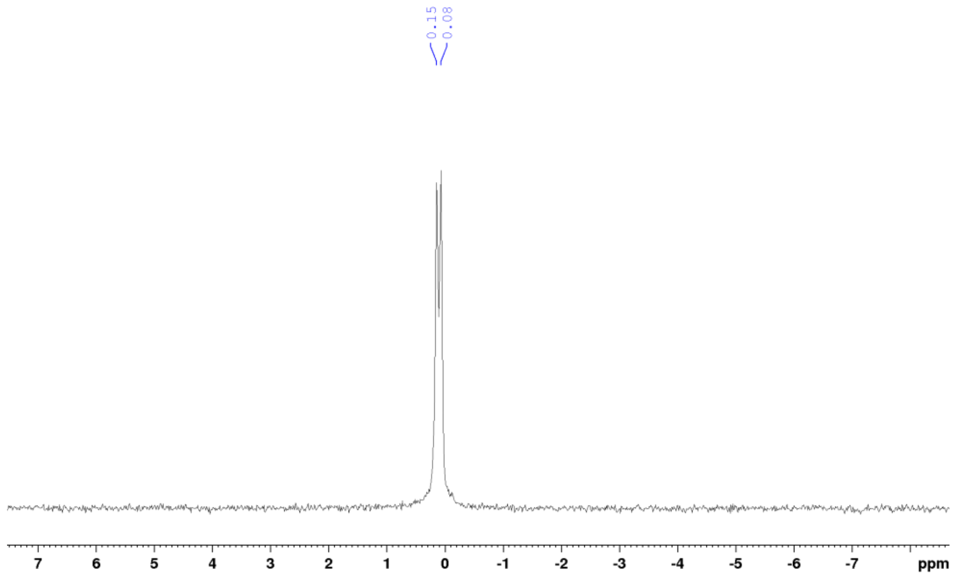

βα-DLAM44, ^{1}H NMR (600 MHz, $CDCl_3$ – CD_3OD, 1:3)

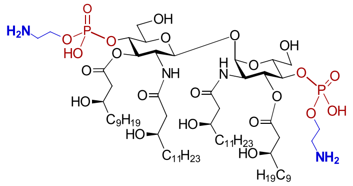

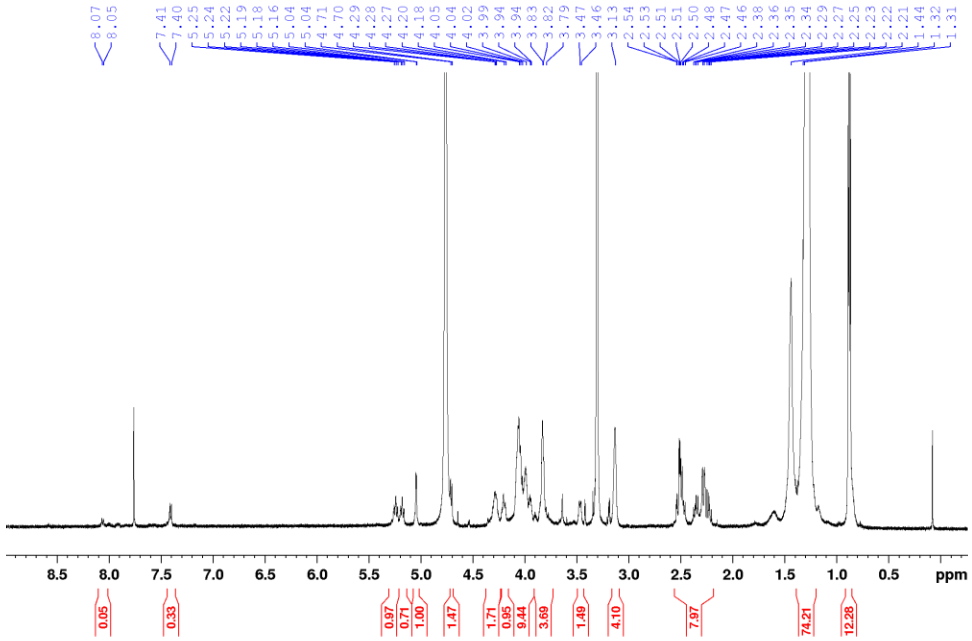

βα-DLAM44,^{13}C NMR ^{1}H-^{13}C HSQC (600/151 MHz, $CDCl_3$ – CD_3OD, 1:3)

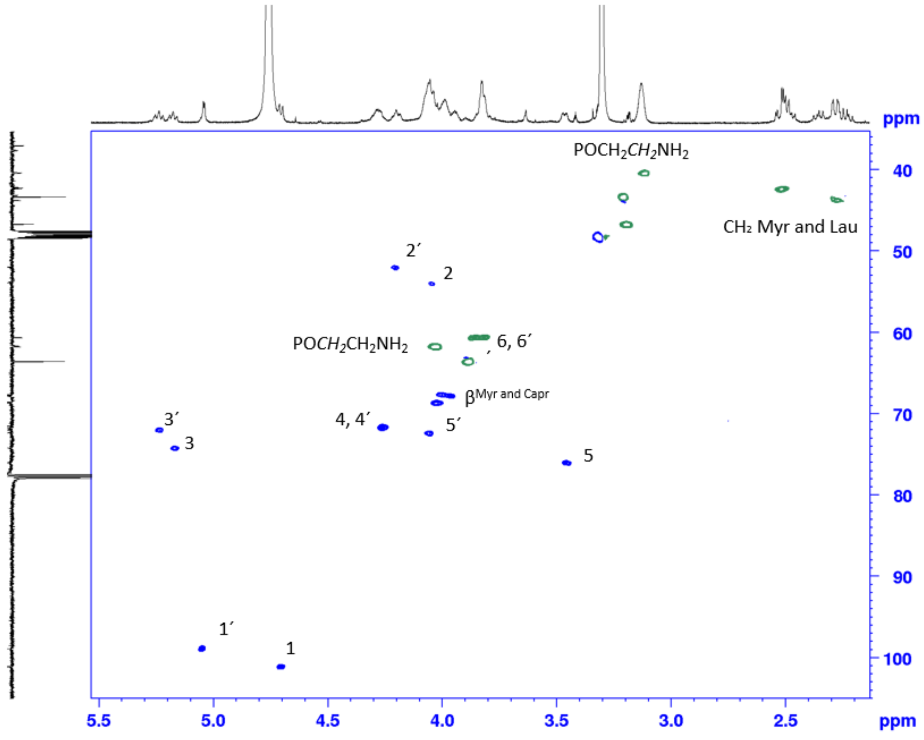

βα-DLAM44, ^{31}P NMR (243 MHz, $CDCl_3$ – CD_3OD, 1:3)

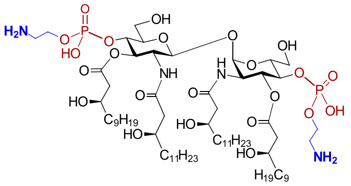

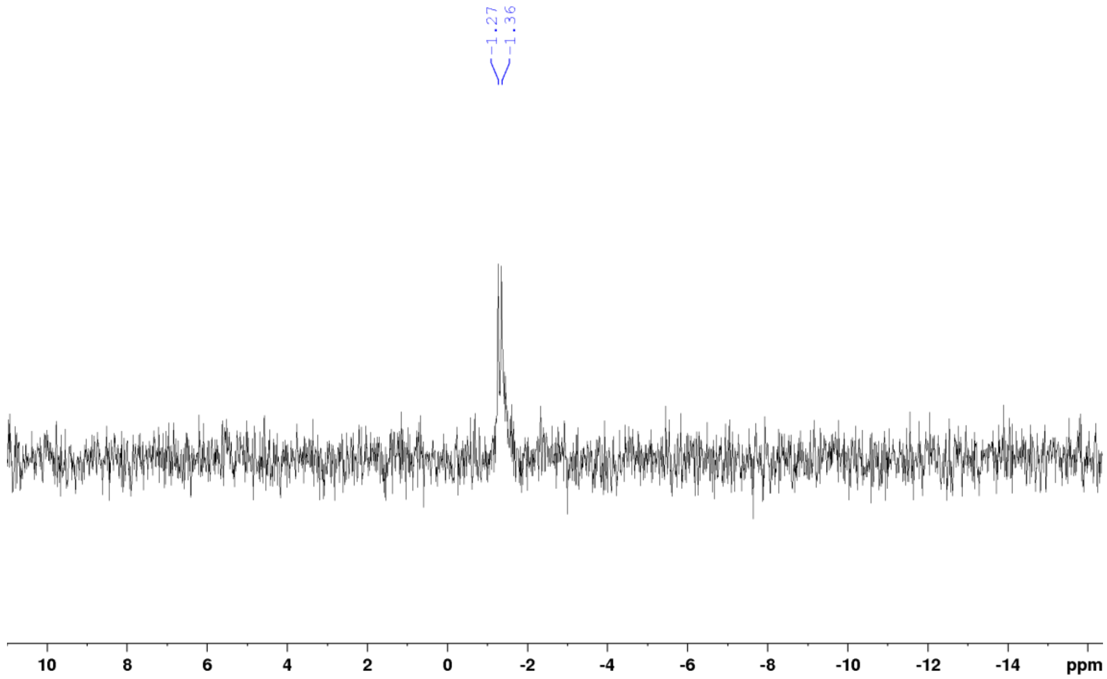

βα-DLAM44, MALDI-TOF MS: DHB, negative mode

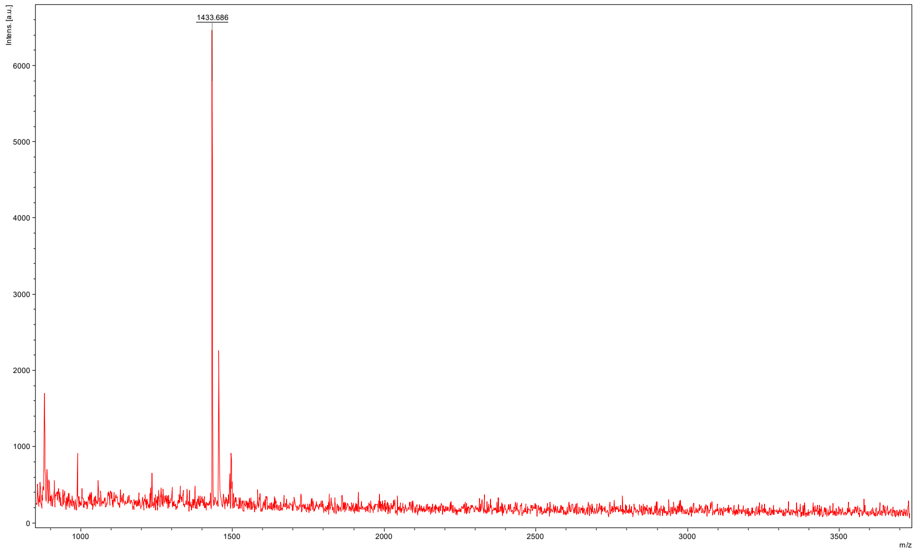

βα-DLAM17, ^{1}H NMR (600 MHz, $CDCl_3$ – CD_3OD, 1:3)

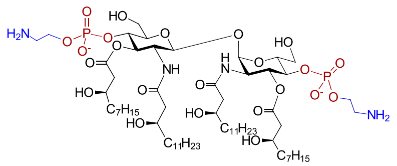

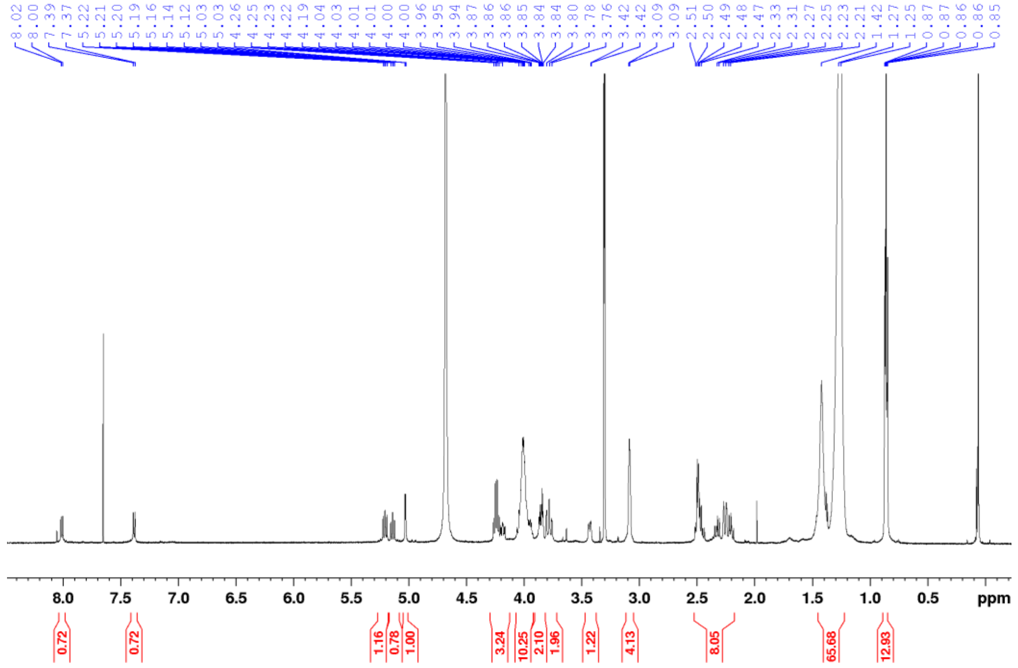

βα-DLAM17, ^{13}C NMR (151 MHz, $CDCl_3$ – CD_3OD, 1:3)

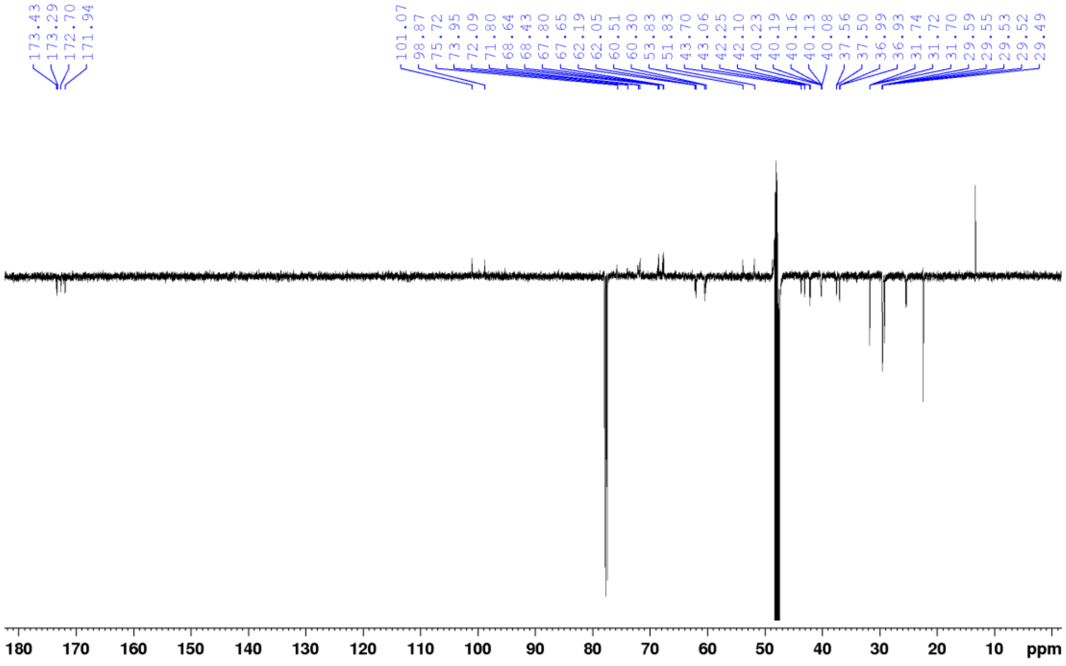

βα-DLAM17, ^{1}H-^{13}C HSQC (600/151 MHz, $CDCl_3$ – CD_3OD, 1:3)

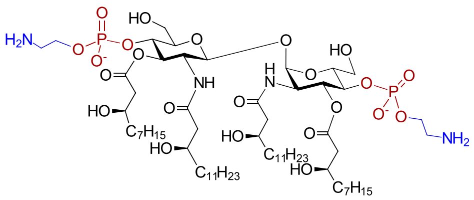

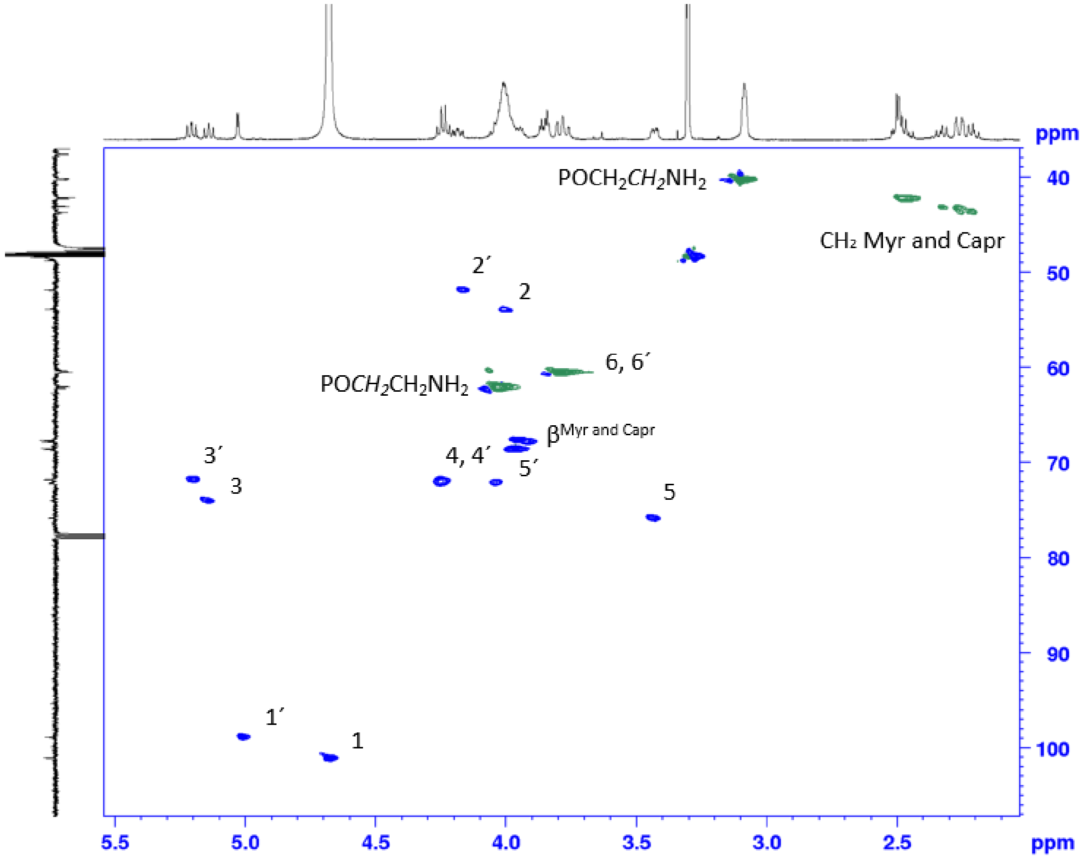

βα-DLAM17, ^{31}P NMR (243 MHz, $CDCl_3 - CD_3OD$, 1:3)

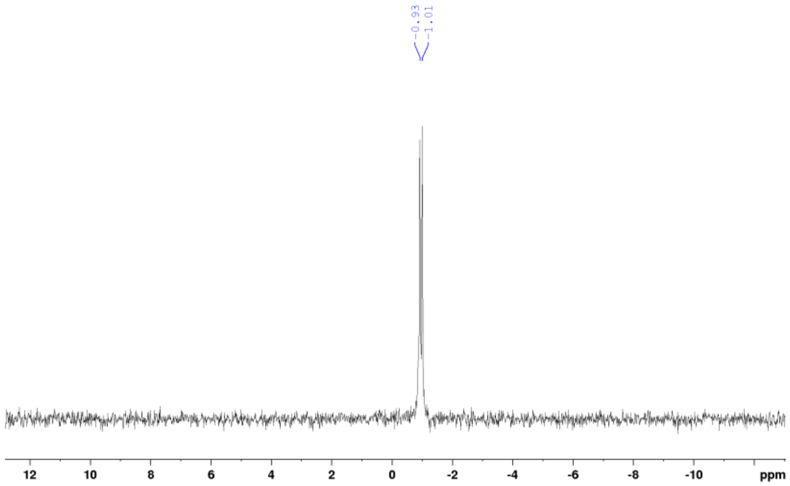

βα-DLAM17, MALDI-TOF MS, ATT, negative mode

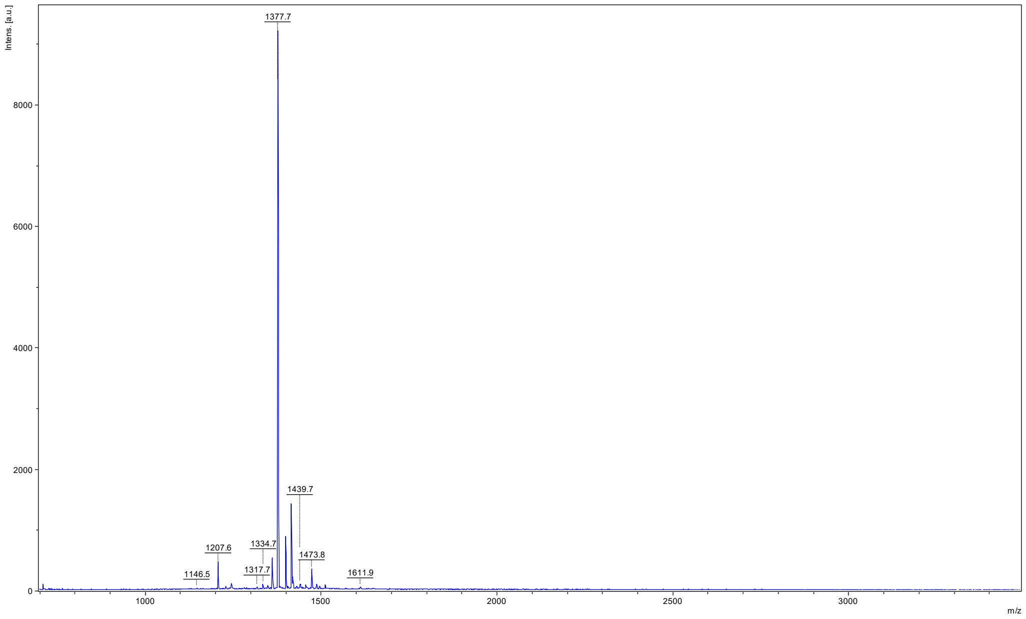

Supplement: CB-007-D5CB00324E-s001 [file CB-007-D5CB00324E-s001.pdf]
